# Tailoring thermally activated delayed fluorescence emitters for efficient electrochemiluminescence with tripropylamine as coreactant†

**DOI:** 10.1039/d3ra06863c

**Published:** 2023-11-24

**Authors:** Luca Morgan, Giulio Pavan, Nicola Demitri, Chiara Alberoni, Thomas Scattolin, Marco Roverso, Sara Bogialli, Alessandro Aliprandi

**Affiliations:** a Dipartimento di Scienze Chimiche, Università degli Studi di Padova via Marzolo 1 35131 Padova Italy alessandro.aliprandi@unipd.it; b Elettra-Sincrotrone Trieste S.C.p.A 34149 Basovizza Trieste Italy

## Abstract

Using a unified metal-free procedure, a selection of Thermally Activated Delayed Fluorescence (TADF) emitters has been synthesized and characterized. Different acceptor and donor moieties have been explored in order to develop red emitting dyes with reduction potentials suitable for the application in ECL using tri-propylamine as coreactant. The most promising compound shows terephthalonitrile as the acceptor and diphenylamines as donors, and it displayed an ECL efficiency that is double the one of the standard [Ru(bpy)_3_](PF_6_)_2_. Based on such findings, a novel water-soluble TADF emitter (Na_4_[4DPASO_3_TPN]) has been synthesized and characterized to enable electrochemiluminescence in an aqueous medium.

## Abstract 2

Bringing thermally activated delayed fluorescence (TADF) emitters from organic solvents to water to enable electrochemiluminescence applications.

## Graphical abstract

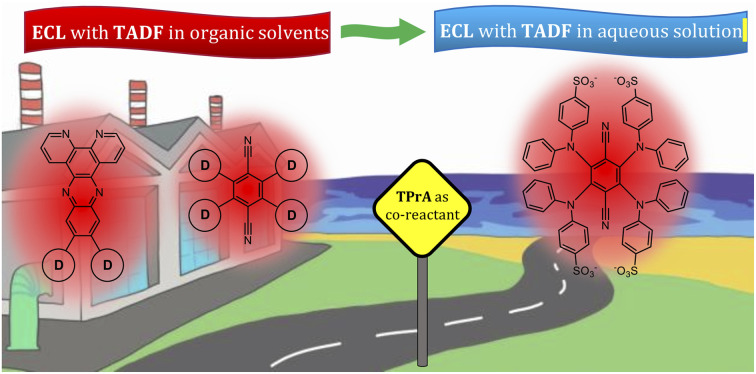

Starting from Adachi's pioneering work,^1^ the development of Thermally Activated Delayed Fluorescence (TADF) emitters has received growing interest,^1,2^ owing to their outstanding photophysical properties. In particular, such compounds have been studied as photocatalysts,^3^ biomarkers^4^ and key components of modern organic light emitting diodes (OLEDs).^5^

One of the main features of organic TADF emitters is the presence of a twisted geometry between donor (D) and acceptor (A) moieties. This characteristic ensures a small energy gap (Δ*E*_ST_) between triplet (T_1_) and singlet (S_1_) excited states, thus favouring the thermally-promoted reverse intersystem crossing (RISC).

Despite the huge number of contributions concerning the use of TADF emitters in the various research areas listed above, only a limited number of recent studies have considered their application in electrochemiluminescence (ECL).^6–8^

In our recent work on the topic we demonstrated that the ECL efficiency of *para*-monoimidazole benzonitrile derivatives is mainly dictated by the electrochemical reversibility of both cathodic and anodic processes rather than the photoluminescence quantum yields (PLQY).^9^

In more detail, the screening of donor moieties showed that diphenylamine and *tert*-butyl carbazole derivatives are the most promising candidates, with ECL efficiencies one order of magnitude higher than the standard [Ru(bpy)_3_]Cl_2_.

However, such ECL efficiencies have been obtained using benzoyl peroxide (BPO) as coreactant at −2 V (*vs.* Ag/AgCl) and no satisfactory ECL emission was observed at anodic potentials with tripropylamine (TPrA) as coreactant.

We hypothesized that such behaviour was due to the too negative reduction potentials (*ca.* −1.7 V *vs.* Ag/AgCl) of the tested compounds.

Since in TADF emitters the reduction potential is mainly dictated by the acceptor moiety, to verify our hypothesis, we have tested terephthalonitrile (TPN) and dipyridophenazine (DPPZ) as acceptor moieties while keeping diphenylamine (DPA) and *tert*-butyl carbazole (^*t*Bu^Cz) as donors as they gave the best performances in our previous study.^9^ In addition, we have also tested di-*para*-tolyl amine (DpTA) with the aim to improve the solubility of terephthalonitrile derivatives and to evaluate the effect of the DPA substitution on the ECL performance.

The target TADF emitters were obtained using a unified and straightforward synthetic procedure, by reacting TPN or difluoro DPPZ with the amine of interest using dry THF or DMF in the presence of NaH as the base (Scheme 1). All compounds were obtained in good to excellent yields and fully characterized by means of NMR, HRMS and XRD analyses (see ESI†).

**Scheme 1:**
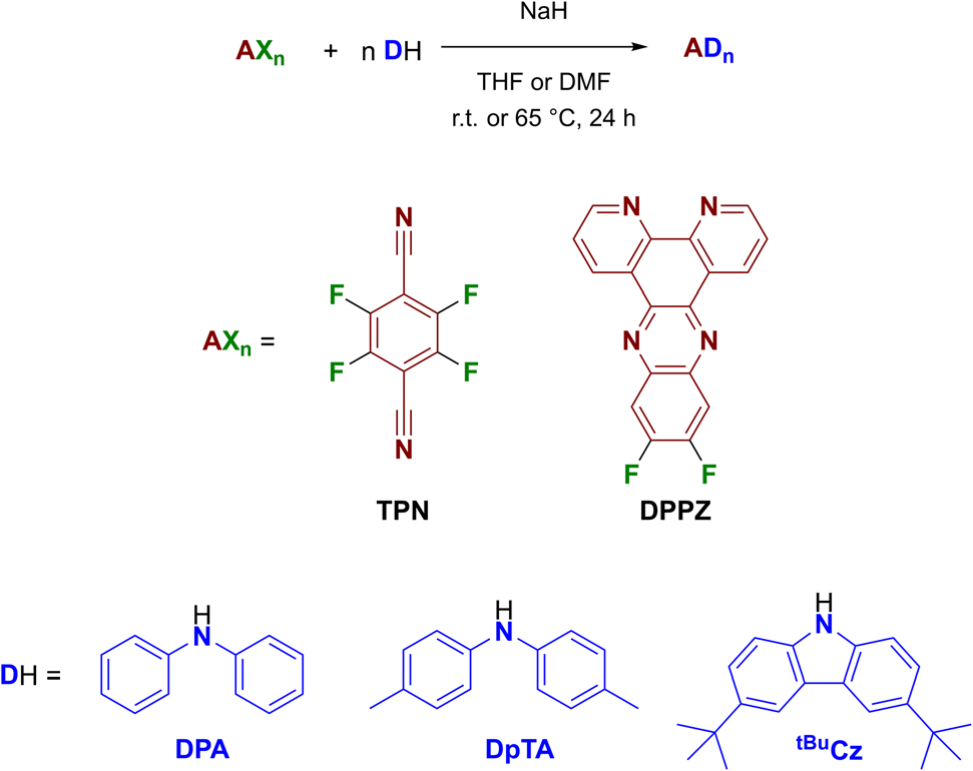
Synthetic procedure of compounds under investigation.

The electrochemical properties of all compounds were studied by cyclic voltammetry in anhydrous dichloromethane with a scan rate of 100 mV s^−1^ and all data are summarized in Table 1, while cyclovoltammetries are reported in ESI (Fig. S1–S5†). Compounds bearing a TPN moiety show a single reversible process in the cathodic region between −1.5 and −1.0 V as observed in their carbazole congener (4CzTPN) by Ishimatsu *et al.*,^6^ while 2DPADPPZ and 2^*t*Bu^CzDPPZ shows a reversible reduction at *ca.* −1.2 V and −1.1 V, respectively that can be ascribed to the reversible reduction of the phenazine core.^10^ As anticipated, the electrochemical process observed in the anodic region is dictated by the DPA and 2^*t*Bu^Cz moieties. In particular, DPA derivatives are characterized by two oxidation processes whose reversibility decrease going from TPN to DPPZ, while 2^*t*Bu^Cz derivatives by a single oxidation process that also becomes more irreversible going from TPN to DPPZ.

**Table 1:** Redox potential of investigated compounds^a^

Compound	*E*_1/2red_ (V)	*E*_1/2ox_ (V)
4DPATPN	−1.39	1.03; 1.26
4DpTATPN	−1.46	0.86; 1.04
4^*t*Bu^CzTPN	−1.09	1.44
2DPADPPZ	−1.22	0.97^b^; 1.30^b^
2^*t*Bu^CzDPPZ	−1.09	1.44^c^
Na_4_[4DPASO_3_TPN]^c^	−1.32	1.22^b^^,^^d^

a Setup and conditions: WE: glassy carbon electrode (3 mm); RE: Ag/AgCl (satd KCl) electrode (in agarose); CE: Pt wire. [compound] = 1 mM. [TBAPF_6_] = 0.1 M. Scan rate: 100 mV s^−1^. Solvent: DCM.

b Irreversible peak.

c Quasi reversible peak; reported potential is peak potential.

d Solvent: DMF.

To assess the effect of the different acceptors and donors on the photophysical properties of the synthesized compounds, absorption and emission spectra were collected and photoluminescence quantum yields (PLQY) and lifetimes of the aerated and degassed solutions were determined (Table 2).

**Table 2:** Photoluminescent properties of investigated compounds in solution^a^

Compound	*λ*_max_ b (nm)	*τ*_p_ c (ns)	*τ*_d_ c (μs)	PLQY^d^ (%)	PLQY^e^ (%)
4DPATPN	597	2.08 ± 0.01	16.53 ± 0.19	8.9 ± 0.3	49.2 ± 1.4
4DpTATPN	625	2.32 ± 0.02	7.19 ± 0.11	6.8 ± 0.3	15.0 ± 0.6
4^*t*Bu^CzTPN	593	3.66 ± 0.01	1.91 ± 0.11	6.2 ± 0.2	6.6 ± 0.3
2DPADPPZ	649	4.21 ± 0.01	—	9.6 ± 0.8	10.3 ± 0.9
2^*t*Bu^CzDPPZ	663	5.77 ± 0.04	—	8.2 ± 0.3	9.2 ± 0.2
Na_4_[4DPASO_3_TPN]^f^	597	6.08 ± 0.04	0.31 ± 0.02	2.7 ± 1.0	2.9 ± 1.1

a Solvent: DCM.

b *λ*_exc_ = 460–584 nm.

c Lifetimes were evaluated on Ar-purged solutions, *λ*_exc_ = 402.3 nm.

d Non-degassed solution; average scan *λ*_exc_ = 300–550 nm.

e Ar-purged solution; average scan *λ*_exc_ = 300–550 nm.

f Solvent: H_2_O.

All compounds exhibit a broad absorption band in the visible region, which is ascribed to a charge transfer transition, and broad emission bands which are typical of organic donor–acceptor compounds (Fig. S7 and S9†).

As far as the concerned TPN derivatives, a remarkable increase in PLQYs moving from aerated to degassed solutions was observed. In particular, for 4DPATPN the PLQY increases from 9% to 49%, thus suggesting a TADF character.

This hypothesis was confirmed by time resolved fluorescence spectroscopy, since the recorded spectra show the typical bi-exponential decay of TADF emitters (Fig. S17–S19†), *i.e.* a short component of 2–3.7 ns ascribed to the prompt fluorescence and a second component of 1–17 μs ascribed to the delayed fluorescence.

Conversely, for DPPZ derivatives no significant increase in PLQYs was observed between aerated and degassed samples. Moreover, mono-exponential decays in the excited state life-times analysis can be observed for these compounds (Fig. S20 and S21†), highlighting the presence only of short components.

It is worth noting that carbazole substituted DPPZ are reported to be TADF emitters into 4,4′-di(9*H*-carbazol-9-yl)-1,1′-biphenyl CBP film suggesting an important effect of the media in the photoluminescent properties of such class of compounds.^11^

Indeed in order to be used as dye for ECL under “oxidative-reduction” conditions, a luminophore has to satisfy two main requirements: (i) its reduction potential has to be higher (more anodic) than the one of TPrA radical anion 
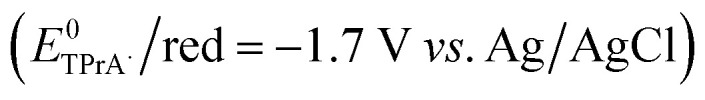
,^12^ and (ii) the luminophore has to display electrochemical reversibility towards oxidation (since in ECL with TPrA a cathodic potential is applied at the electrode^12^). Those luminophores that satisfy both these requirements should perform better.

Table 3 summarizes the results obtained by testing the different compounds as ECL dyes with TPrA as coreactant. No significant differences were observed from the comparison of the ECL emission and photoluminescence emission profiles of the luminophores (Fig. S28–S32†), suggesting that no excimers or exciplexes are involved in light generation.

**Table 3:** ECL efficiencies with coreactant in solution^a^

Compound	ECL efficiency^b^ (*φ*_ECL_)	*λ*_max_ ECL
4DPATPN	1.72 ± 0.37	595
4DpTATPN	0.33 ± 0.08	621
4^*t*Bu^CzTPN	0.30 ± 0.08	601
2DPADPPZ	0.019 ± 0.004	650
2^*t*Bu^CzDPPZ	0.011 ± 0.002	672
Na_4_[4DPASO_3_TPN]^c^	0.02 ± 0.01	597

a Setup and conditions: WE: GCE (3 mm); RE: Ag/AgCl (satd KCl) electrode (in agarose); CE: Pt wire. [Compound] = 50 μM; [TPrA] = 1 mM; [TBAPF_6_] = 0.1 M. Solvent: DCM. Potential sequence (repeated three times): 0 V for 3 s, 1.5 V for 3 s, 0 V for 3 s. All experiments were performed three times each.

b Calculated with eqn (S1) assuming ECL efficiency of [Ru(bpy)_3_](PF_6_)_2_ equal to 1.

c [Compound] = 2 μM. Solvent: ProCell solution.

Furthermore, the potential onset for light emission is localized around the oxidation potential of the luminophores, indicating that the determining step in ECL light production is the formation of their radical cation species (Fig. S23–S27†).

The ECL efficiency (*φ*_ECL_) of the emitters was evaluated by performing a chronoamperometry applying a sequence of pulsed electric potentials (1.5 V, 0.3 Hz). *φ*_ECL_ of the compounds was determined according to eqn (S1),† by comparing their total ECL intensities with the one of [Ru(bpy)_3_](PF_6_)_2_, used as standard and considered with unitary ECL efficiency.^2^

It is not surprising that 4DPATPN, which possess the best PLQY among the presented compounds and displays electrochemical reversibility, outperforms all the other luminophores.

Interestingly, the ECL efficiency of 4DPATPN is about double that of the benchmark [Ru(bpy)_3_](PF_6_)_2_.

Even though 4DpTATPN, 4^*t*Bu^CzTPN, 2DPADPPZ and 2^*t*Bu^CzDPPZ possess comparable PLQYs, TPN derivatives display ECL performances one order of magnitude higher than DPPZ based emitters. This behavior can be ascribed to the fact that TPN derivatives show higher electrochemical reversibility of the redox processes and the capacity to harvest triplet excited states by the RISC process. In addition, the presence of methyl groups on the DPA, 4DpTATPN, results in a significant quenching of the PLQY leading to a lower ECL efficiency.

We carried out annihilation experiments only on TPN derivatives since they are the most ECL active and possess reversible electrochemical processes both in oxidation and reduction. The potential was stepped between the first reduction and the first oxidation process. For all the investigated compounds (Fig. S39–S41†), interestingly, almost the same trend of ECL efficiencies reported with the coreactant mode was observed for the annihilation experiments (Table 4). In particular, 4DPATPN outperforms the standard [Ru(bpy)_3_](PF_6_)_2_ more than 6 times.

**Table 4:** ECL annihilation efficiencies in DCM solution^a^

Compound	Applied potentials	ECL efficiency^b^ (*φ*_ECL_)
[Ru(bpy)_3_](PF_6_)_2_	−1.5 V and +1.5 V	1.00 ± 0.67
	+1.5 V and −1.5 V	1.00 ± 0.48
4DPATPN	−1.6 V and +1.1 V	6.50 ± 3.38
	+1.1 V and −1.6 V	6.90 ± 2.74
4DpTATPN	−1.6 V and +1.0 V	5.27 ± 2.50
	+1.0 V and −1.6 V	5.18 ± 1.76
4^*t*Bu^CzTPN	−1.2 V and +1.6 V	1.33 ± 0.65
	+1.6 V and −1.2 V	1.62 ± 0.73

a Setup and conditions: WE: GCE (3 mm); RE: Ag/AgCl (satd KCl) electrode (in agarose); CE: Pt wire. [Compound] = 0.2 mM; [TBAPF_6_] = 0.1 M. Solvent: DCM. Potentials were stepped with a frequency of 10 Hz and their values are reported near the respective compound.

b Calculated with eqn (S1) assuming ECL efficiency of [Ru(bpy)_3_](PF_6_)_2_ equal to 1.

Considering that ECL in aqueous media is fundamental for bioanalytical applications,^13^ we wondered if it was possible to develop a TADF emitter that can be adopted as chromophore for ECL in aqueous media. To this end, recently Zeng *et al.* reported ECL emission of 4CzIPN in aqueous PBS buffer solution, thanks to nanoencapsulation of the TADF emitter in micelles.^14^ Concomitantly, Zhang *et al.* reported the first example of aggregation-induced delayed fluorescence (AIDF) luminophores applied to ECL in aqueous media.^15^ Nevertheless, a molecularly dissolved, water-soluble and purely organic TADF emitter for aqueous ECL applications is still unreported.

Based on the promising ECL results obtained with 4DPATPN, we designed a water-soluble TADF emitter by replacing DPA with sodium diphenylamine-4-sulfonate (Na(DPASO_3_)) as donor moiety. The target Na_4_[4DPASO_3_TPN] compound was isolated in excellent yields following the standard protocol illustrated in Scheme 2.

**Scheme 2:**
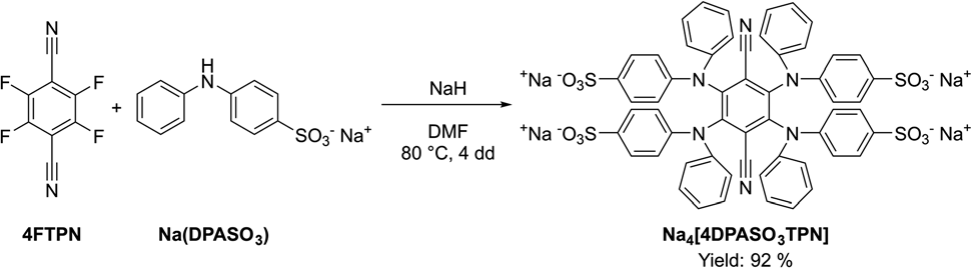
Synthetic procedure of Na_4_[4DPASO_3_TPN].

Cyclic voltammetry of Na_4_[4DPASO_3_TPN] in anhydrous DMF was studied, showing one reversible reduction process and irreversible oxidation processes (Fig. S6†).

Room-temperature ultraviolet-visible (UV-vis) absorption and PL spectra in dilute water solution were recorded and they are shown in Fig. S8 and S10.† The absorption and emission profiles are similar to those of 4DPATPN, with the same maximum value of emission (597 nm) but with a slight broadening of the spectrum.

In contrast to the other reported TPN derivatives (see Table 2), no relevant increase in PLQY moving from aerated to degassed water solutions was observed (2.63 ± 0.01% *vs.* 2.92 ± 0.01%). Moreover, the time resolved photoluminescent decays were measured in water recording lifetimes of 7.25 ns and 0.3 μs, attributed to prompt and delayed fluorescence, respectively (Fig. S22†). Typically, the PLQY decreases as the solvent polarity increases due to the stabilization of the excited state,^16^ however the delayed fluorescence component is still observed, albeit shorter, indicating that the RISC process is still present in aqueous medium. To the best of our knowledge, this is the first example of a water-soluble, purely organic compound with TADF character.

Na_4_[4DPASO_3_TPN] was tested as dye in ECL application using the ProCell® as reaction medium. Procell is a 0.3 M phosphate buffer (pH 6.8) containing 180 mM TPrA as co-reactant and an undisclosed surfactant mixture.

Despite the low PLQY, Na_4_[4DPASO_3_TPN] exhibited a good ECL efficiency compared to the standard [Ru(bpy)_3_]Cl_2_ (0.02 ± 0.01, assuming ECL efficiency of [Ru(bpy)_3_]Cl_2_ equal to 1). Despite the low ECL efficiency, this represents the first example of aqueous ECL emission by a molecularly dissolved TADF emitter.

In conclusion, a selection of thermally activated delayed fluorescence luminophores have been synthesized through a unified synthetic procedure involving different acceptor precursors and amines using dry THF or DMF in the presence of NaH as base. All compounds have been structurally characterized and their electrochemical and photophysical properties have been investigated. The luminophores have been tested as emitters in ECL applications using TPrA as coreactant and 4DPATPN displayed almost a double ECL efficiency compared to the standard [Ru(bpy)_3_](PF_6_)_2_, thanks to its high PLQY and electrochemical reversibility. Furthermore, it has been confirmed that increasing the acceptor strength anodically shifts the reduction potential allowing efficient ECL in the presence of TPA as coreactant.

The synthesis of novel water-soluble TADF emitter (Na_4_[4DPASO_3_TPN]) allowed us to determine the ECL efficiency of an organic dye using ProCell as the reaction medium. We strongly believe that the promising results of this work can pave the way for the development of water-soluble dyes with performances comparable or even better than the standard Ru(bpy)_3_^2+^.

## Conflicts of interest

There are no conflicts to declare.

## Supplementary Material

RA-013-D3RA06863C-s001

RA-013-D3RA06863C-s002
