# Supplementary material for: Tailoring thermally activated delayed fluorescence emitters for efficient electrochemiluminescence with tripropylamine as coreactant
Source: RSC Adv. 2023 Nov 24;13(49):34520–3. doi: 10.1039/d3ra06863c (PMC10668078; doi:10.1039/d3ra06863c)
Supplement: RA-013-D3RA06863C-s001 [file RA-013-D3RA06863C-s001.pdf]

Electronic Supplementary Material (ESI) for RSC Advances.
This journal is © The Royal Society of Chemistry 2023

Electronic Supplementary Information

Tailoring Thermally Activated Delayed Fluorescence Emitters for Efficient Electrochemiluminescence with Tripropylamine as Coreactant

Luca Morgan[a], Giulio Pavan[a], Nicola Demitri[b], Chiara Alberoni[a], Thomas Scattolin[a]*, Marco Roverso[a], Sara Bogialli[a] and Alessandro Aliprandi[a]*

[a]Dipartimento di Scienze Chimiche, Università degli Studi di Padova, via Marzolo 1, 35131 Padova, Italy.
[b]Elettra-Sincrotrone Trieste S.C.p.A, 34149 Basovizza, Trieste, Italy.

Table of contents

1. General methods **S2**
2. Synthesis route and procedures **S5**
3. Electrochemical characterization **S9**
4. Photophysical characterization **S15**
5. Electrochemiluminescence characterization **S23**
6. Single crystal X-ray diffraction analysis **S34**
7. Supplementary references **S36**
8. ^{1}H and {^{1}H}^{13}C NMR spectra of compounds **S37**

1. General Methods

All reagents, except 3,6-di-*tert*-butyl-carbazole and 11,12-difluorodipyridophenazine (synthesized according to literature[1,2]), were used as received without further purification and they were obtained from Sigma-Aldrich and TCI.

All solvents, except those for photophysical measurements, were dried according to literature procedure[3] using 3Å molecular sieves purchased from Thermoscientific.

^{1}H and {^{1}H}^{13}C NMR spectra were recorded on a Bruker AVANCE III spectrometer equipped with a BBO probe; ^{1}H and {^{1}H}^{13}C NMR chemical shifts were referenced relative to residual solvents peaks[4].

NMR solvents were purchased from Sigma-Aldrich or EurisoTOP and dried with 3Å molecular sieves purchased from Merck.

Multiplicities of signals are reported as s (singlet), d (doublet), t (triplet), m (multiplet) and combinations of these multiplicities.

HR-MS spectra were recorded on a Thermo Q-Exactive™ equipped with an ESI source. Mass spectra were recorded in positive (or negative) ionization mode, resolution 70000 and mass range 300-4000 amu. Ionization was performed by setting the following source parameters: capillary voltage 4.0 kV, S-Lens RF Level 55, capillary temperature 300 °C and probe temperature 320 °C; nitrogen was used as sheath and auxiliary gas at 40 psi and 20 arbitrary units, respectively. Samples were prepared using dichloromethane as solvent and injected for analysis at a flow rate of 10 L/min. Calibration was performed with a standard solution purchased from Thermo FisherScientific (Pierce®ESI positive Ion Calibration Solution). The software for analysis of MS data was Xcalibur 4.2 (Thermo Fisher Scientific).

All reactions were monitored by thin-layer chromatography using Merck silica gel plates 60 F_{254} (plastic sheets) and spots were visualized with UV light.

Flash chromatography was performed on a Combiflash Nextgen 300+ using Redisep Bronze column (12 grams) filled with NP silica gel and using Redisep Rf cartridge (4 and 24 grams).

1.1 Electrochemical and electrochemiluminescence measurements.

Electrochemical measurements were performed with a Autolab PGstat302N as potentiostat using NOVA 2.1.5 as interface software.

The configuration of electrochemical cell was the three-electrode configuration, which consists in a glassy carbon electrode (diameter 3 mm) as working electrode (WE), a silver/silver chloride reference electrode (RE) built according to a literature procedure[5] and a platinum wire as counter electrode (CE).

The glassy electrochemical cell was specially made with a Teflon cap at the head and a flat glass window at the bottom for the detection of the generated ECL signal.

Before every experiment the solution was Ar-bubbled for 5 minutes to remove oxygen and every experiment was performed under a protective atmosphere of argon; finally, after every measurement the working electrode was polished with alumina slurry (0.05 mm).

Cyclic voltammetry measurements were performed with dried HPLC-grade dichloromethane solution containing 1 mM of investigated compounds and 0.1 M tetrabutylammonium hexafluorophosphate as supporting electrolyte with a scan rate equal to 100 mV/s.

ECL-voltage curve measurements were performed with dried HPLC-grade dichloromethane solution containing 0.05 mM of investigated compounds, 1 mM of tri-propyl amine as co-reactant and 0.1 M tetrabutylammonium hexafluorophosphate as supporting electrolyte. The scan rate was 20 mV/s and to detect ECL signals the electrochemical cell was put over an Ocean Insight 1 mm optical fiber linked to a Thorlabs 1001/M photomultiplier tube.

PMT 2100 control was used as software interface for photomultiplier tube (PMT gain: 40 for $Na_4[4DPASO_3TPN]$ and 80 for other compounds, Bandwidth 80 MHz).

ECL spectra were performed with dried HPLC-grade dichloromethane solution containing 0.05 mM of investigated compounds, 1 mM of tripropylamine as coreactant and 0.1 M tetrabutylammonium hexafluorophosphate as supporting electrolyte. The ECL signal was obtained by applying a potential of -2 V to the solution and to detect ECL signals (integration time: 3s) the electrochemical cell was put over a 1 mm optical fiber linked to a CCD QEPro camera.

ECL measurements were performed with dried HPLC-grade dichloromethane solution containing 0.05 mM of investigated compounds, 1 mM of tripropylamine as coreactant and 0.1 M tetrabutylammonium hexafluorophosphate as supporting electrolyte; the following scheme was repeated three times: 0 V for 3 seconds, 1.5 V for 3 seconds and 0 V for 3 seconds.

ECL measurements of $Na_4[4DPASO_3TPN]$ was performed with ProCell solution containing 2 μM of investigated compounds and 0.1 M tetrabutylammonium hexafluorophosphate as supporting electrolyte; the following scheme was repeated three times: 0 V for 3 seconds, 1.5 V for 3 seconds and 0 V for 3 seconds.

Every ECL experiment was repeated three times for every compound in order to calculate average values, and relative efficiencies were determined by comparison with standard $[Ru(bpy)_3](PF_6)_2$ by taking its efficiency as 1.

ECL efficiencies are calculated using **Equation (S1)**[6].

$$\Phi_{x,ECL} = \frac{\left(\frac{\int_a^b ECL\, dt}{\int_a^b i\, dt}\right)_x}{\left(\frac{\int_a^b ECL\, dt}{\int_a^b i\, dt}\right)_{St}} \quad \text{(S1)}$$

where *x* represents the investigated compound and *St* represents the standard $[Ru(bpy)_3](PF_6)_2$.

1.2 Photophysical measurements

All samples were prepared in spectroscopic-grade Uvasol® dichloromethane, that were used as received, with a $5 \cdot 10^{-6}$ M concentration.

Absorption spectra were recorded with a Varian Cary 100bio UV-VIS spectrophotometer in 10 mm quartz cuvettes filled with non-degassed solution of investigated compounds.

Emission and excitation spectra were recorded with a Photoluminescence Spectrometer (Edinburgh Instruments, FLS1000) equipped with a 450 W Xe lamp and an air-cooled single-photon counting photomultiplier (Hamamatsu R13456). The solutions of investigated compounds, contained in a10 mm quartz cuvette, were bubbled with argon for 15 minutes prior measurements.

Absolute PhotoLuminescence Quantum Yields of solution were obtained with a Hamamatsu Absolute PL quantum yield spectrometer C11347 Quantaurus QY using a quartz tube filled with non-degassed or Ar-purged solutions of investigated compounds.

Lifetimes were obtained on a FLS1000 in Ar-purged or non-degassed solutions.

Time-resolved lifetime measurements were acquired on the FLS1000 spectrometer equipped with a picosecond laser diode at 402.3 nm. Luminescence decays were recorded using the Time correlated single photon counting (TCSPC) for lifetime in the nanosecond range, or the Multi-channel scaling (MCS) technique for lifetime in the microsecond range. The frequency of the laser was changed according to the lifetime of each sample (from 100 kHz to 30 MHz).

Decay curves were analyzed with the Fluoracle Software using the IRF convolution fitting (for lifetime in ns range) or the tail-fitting for longer lifetimes.

1.3 Single crystal X-Ray Diffraction analysis

Suitable single crystals for X-Rays Diffractometry were obtained by slow diffusion or by slow evaporation of solvents. Solvent mixtures that were employed are reported in **Synthesis route and procedures** part.

Crystallographic data have been deposited at the Cambridge Crystallographic Data Centre and allocated the deposition numbers CCDC 2284117 (for 4DpTATPN), 2284118 (for 4DPATPN) and 2284119 (for 2DPADPPZ). These data can be obtained free of charge via https://www.ccdc.cam.ac.uk/structures.

Data collections were performed at the XRD2 beamline of the Elettra Synchrotron, Trieste (Italy).[7] The crystals were dipped in NHV oil (Jena Bioscience, Jena, Germany) and mounted on the goniometer head with kapton loops (MiTeGen, Ithaca, USA). Complete datasets were collected at 100 K (nitrogen stream supplied through an Oxford Cryostream 700) through the rotating crystal method. Data were acquired using monochromatic wavelength of 0.620 Å on a Pilatus 6M hybrid-pixel area detector (DECTRIS Ltd., Baden-Daettwil, Switzerland). Diffraction data were indexed, integrated and scaled using XDS.[8] Structures were solved by the dual space algorithm implemented in the SHELXT code.[9] Fourier analysis and refinement were performed by the full-matrix least-squares methods based on F2 implemented in SHELXL (Version 2019/3).[10] The Coot program was used for modeling.[11] Anisotropic thermal motion refinement was used for all atoms with occupancies higher than 50%. Geometry and thermal motion parameters restrains (SIMU, DFIX and DANG) were used on disordered solvent molecules. Hydrogen atoms were included at calculated positions with

isotropic $U_{factors} = 1.2 \cdot U_{eq}$ or $U_{factors} = 1.5 \cdot Ueq$ for methyl groups (U_{eq} being the equivalent isotropic thermal factor of the bonded non hydrogen atom). All the crystal showed solvent molecules trapped into crystal voids. Electron density contributions that couldn't be modelled in 2DPADPPZ were removed with Platon SQUEEZE[12] routine. Estimated contributions of these regions are included in the reported crystal properties (Table S1). Pictures were prepared using Ortep-3[13] and Pymol[14] software.

Compounds crystallize in centrosymmetric space groups with half molecule in the asymmetric units (ASU) for diciano derivatives 4DpTATPN and 4DPATPN, while multiple copies have been found for 2DPADPPZ (Figure S42). 4DpTATPN and 4DPATPN molecules lies on crystallographic inversion centres perfectly matching molecular symmetry. Crystal packing contacts essentially involves $\pi \cdots \pi$ stacking and $CH \cdots \pi$ contacts through phenyls of sidechains.

Not equivalent molecules adopt very similar conformations with minor lateral phenyl rearrangements, as shown in Figure S43 (R.M.S.D. < 0.5 Å among common atoms).

2. Synthesis routes and procedures

2.1 Synthesis of 2,3,5,6-tetrasubstituted-terephthalonitrile derivatives

The 2,3,5,6-tetrasubstituted-terephthalonitrile derivatives were synthesized by slight modification of a reported procedure[15] with the aim to uniform the synthetic route for all samples under investigation.

In a flame dried and argon-purged round-bottomed flask equipped with a magnetic stir bar 6 equivalents of donor compound were put in dry THF. Then 10 equivalents of sodium hydride were added (60% in mineral oil), and the suspension was stirred for 1 h at 65 °C. Finally, 1 equivalent of 2,3,5,6-tetrafluorine-terephthalonitrile as acceptor was added and the resulting mixture was stirred at 65 °C for 16 h.

A precipitate was formed, the reaction was quenched by addition of water and the precipitate was filtered on Gooch (porosity 4) and washed with water and diethyl ether. The products were bright red powders and they were obtained with a good yield.

2.1.1 Synthesis of 2,3,5,6-diphenyl-amine-terephthalonitrile (4DPATPN)

According to the general procedure, di-phenyl-amine (DPA) as donor was used and a bright red precipitate appeared during the reaction. The product was obtained with a good yield (86%).

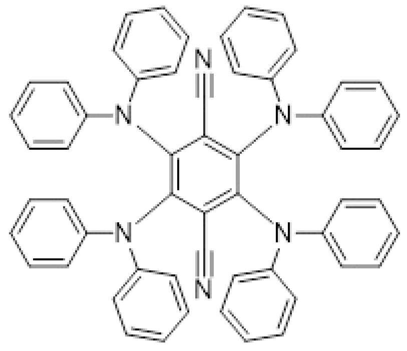

^{1}H NMR (300 MHz, DMF-d_7) δ 7.15 (t, 2H), 7.01 (d, 2H), 6.87 (t, 1H) ppm.

^{13}C NMR (75 MHz, DMF-d_7) δ 145.8, 129.6, 124.0, 123.0, 55.6 ppm.

HR-MS (*m/z*) Calculated: 819.3207 ([M+Na]$^+$); Found: 819.3217 ([M+Na]$^+$).

Suitable crystals for SC-XRD were obtained by slow diffusion of acetone vapors into 1,2-dichloroethane.

2.1.2 Synthesis of 2,3,5,6-di-*p*-tolyl-amine-terephthalonitrile (4DpTATPN)

According to the general procedure, di-*p*-tolyl-amine (DpTA) as donor was used and a bright red precipitate appeared during the reaction. The product was obtained with a high yield (86%).

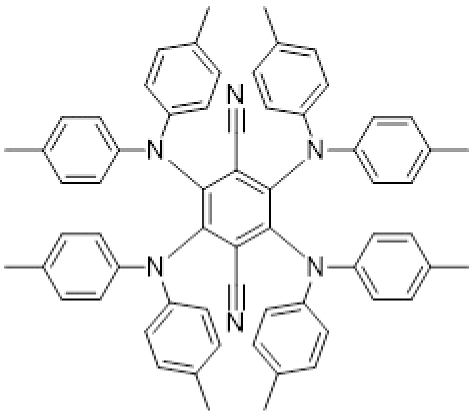

^{1}H NMR (300 MHz, CD_2Cl_2) δ 6.93 (d, 2H), 6.63 (d, 2H), 2.20 (s, 3H) ppm.

^{13}C NMR (75 MHz, CD_2Cl_2) δ 143.3, 133.3, 129.8, 122.4, 21.0 ppm.

HR-MS (*m/z*) Calculated: 909.4639 ([M+H]$^+$); Found: 909.4636 ([M+H]$^+$).

Suitable crystals for SC-XRD were obtained by slow diffusion of hexane vapors into chloroform.

2.1.3 Synthesis of 2,3,5,6-di-*tert*-butyl-carbazole-terephthalonitrile (4^{tBu}CzTPN)

According to the general procedure, di-*tert*-butyl-carbazole (^{tBu}Cz) as donor was used and a bright red precipitate appeared during the reaction. The product was obtained with a good yield (75%).

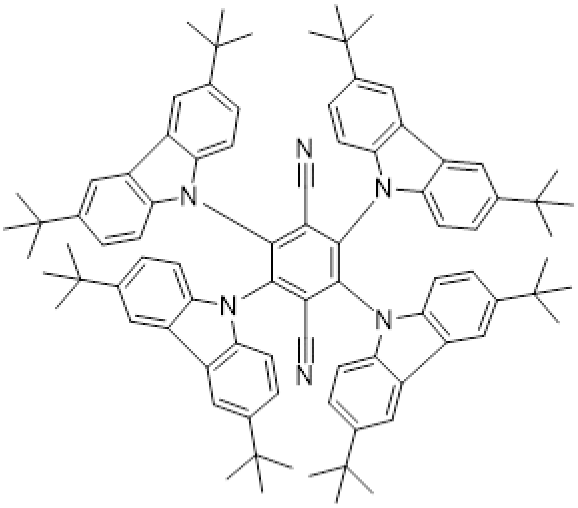

^{1}H NMR (300 MHz, CD_2Cl_2) δ 7.66 (d, 1H), 7.11 (dd, 1H), 7.03 (d, 1H), 1.39 (s, 9H) ppm.

^{13}C NMR (75 MHz, CD_2Cl_2) δ 145.3, 139.3, 137.4, 125.2, 123.8, 116.7, 109.9, 35.1, 32.3 ppm.

HR-MS (m/z) Calculated: 1237.7769 ([M]); Found: 1237.7802 ($[M+H]^+$) .

2.2 Synthesis of 2,3,5,6- diphenilamine-4-sulfonate-terephthalonitrile ($Na_4[4DPASO_3TPN]$)

In a flame dried and argon-purged round-bottomed flask equipped with a magnetic stir bar 4.5 equivalents of diphenilamine-4-sulfonate ($Na(DPASO_3)$) compound were put in dry DMF. Then 9 equivalents of sodium hydride were added (60% in mineral oil), and the solution was stirred for 1 h at 80 °C. Finally, 1 equivalent of 2,3,5,6-tetrafluorine-terephthalonitrile as acceptor was added and the resulting mixture was stirred at 80 °C for 4 days.

The reaction was quenched by addition of water, the mixture was concentrated at reduced pressure and the precipitation of the product was achieved upon addition of acetonitrile. The precipitate was filtered on Gooch (porosity 4) and washed with acetonitrile. The product was obtained in high yield (92 %) as a red powder.

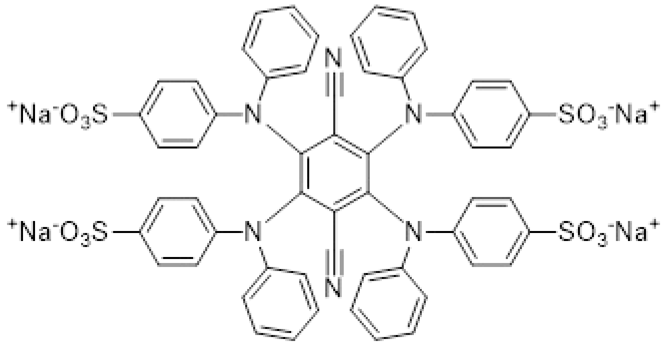

^{1}H NMR (300 MHz, DMSO-d_6) δ 7.35 (d, 2H), 7.15 (t, 1H), 6.90-6.75 (m, 6H) ppm.

^{13}C NMR (75 MHz, D_2O) δ 171.9, 147.2, 145.8, 144.4, 138.1, 130.2, 127.4, 125.5, 123.3, 121.8 ppm.

HR-MS (m/z) Calculated: 278.0324 ($[M]^{4-}$); Found: 278.0303 ($[M]^{4-}$).

2.3 Synthesis of di-pyridyl-phenazil-disubstituted derivatives

In a flame dried and argon-purged round-bottomed flask equipped with a magnetic stir bar 3 equivalents of donor compound were put in dry THF. Then 6 equivalents of sodium hydride were added (60% in mineral oil), and the suspension was stirred for 1 h. Finally, 1 equivalent of 11,12-difluorodipyridophenazine as acceptor was added and the resulting mixture was stirred for 4 h.

2.3.1 Synthesis of diphenylamine-pyridyl-phenazine (2DPADPPZ)

According to the general procedure, diphenylamine (DPA) as donor was used, the reaction mixture was heated to 65 °C and a dark red powder was obtained in a good yield (68%).

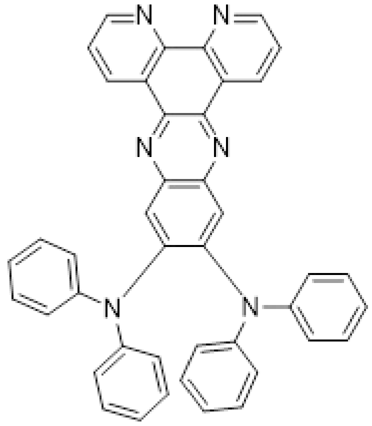

^{1}H NMR (300 MHz, CD_2Cl_2) δ 9.46 (dd, 2H), 9.15 (dd, 2H), 7.88 (s, 2H), 7.72 (dd, 2H), 7.25 (t, 8H), 7.08 (t, 4H), 6.87 (d, 8H) ppm.

^{13}C NMR (75 MHz, CD_2Cl_2) δ 152.2, 148.7, 148.2, 147.4, 141.4, 140.1, 133.5, 129.0, 127.8, 125.7, 124.3, 124.1 ppm.

HR-MS (m/z) Calculated: 617.2448 ($[M+H]^+$); Found: 617.2459 ($[M+H]^+$).

Suitable crystals for SC-XRD were obtained by slow diffusion of hexane vapors into chloroform.

2.3.2 Synthesis of di-*tert*-butyl-carbazole-pyridyl-phenazine (2^{tBu}CzDPPZ)

According to the general procedure, di-*tert*-butyl-carbazole (^{tBu}Cz) as donor was used the reaction mixture was stirred at room temperature and an orange powder was obtained in a good yield (66%).

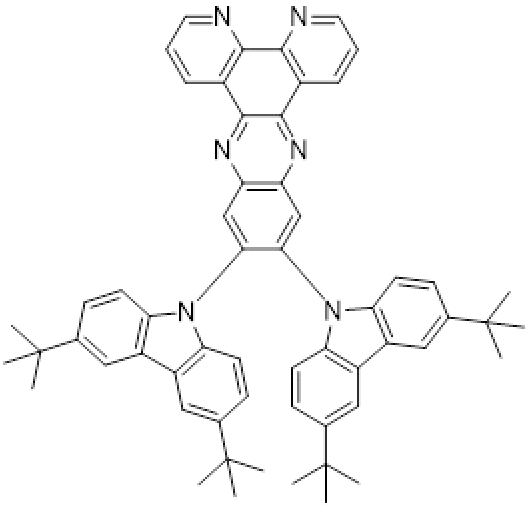

^{1}H NMR (300 MHz, $CDCl_3$) δ 9.68 (dd, 2H), 9.32 (dd, 2H), 8.82 (s, 2H), 7.83 (m, 2H), 7.64 (d, 4H), 6.94 (m, 8H), 1.35 (s, 36H) ppm.

^{13}C NMR (75 MHz, $CDCl_3$) δ 153.0, 148.8, 1413.4, 142.1, 141.8, 138.2, 137.5, 134.1, 130.1, 127.6, 124.5, 124.0, 123.0, 115.6, 109.0, 34.6, 32.0 ppm.

HR-MS (m/z) Calculated: 837.4639 ($[M+H]^{+}$); Found: 837.4651 ($[M+H]^{+}$).

3. Electrochemical characterization

a)

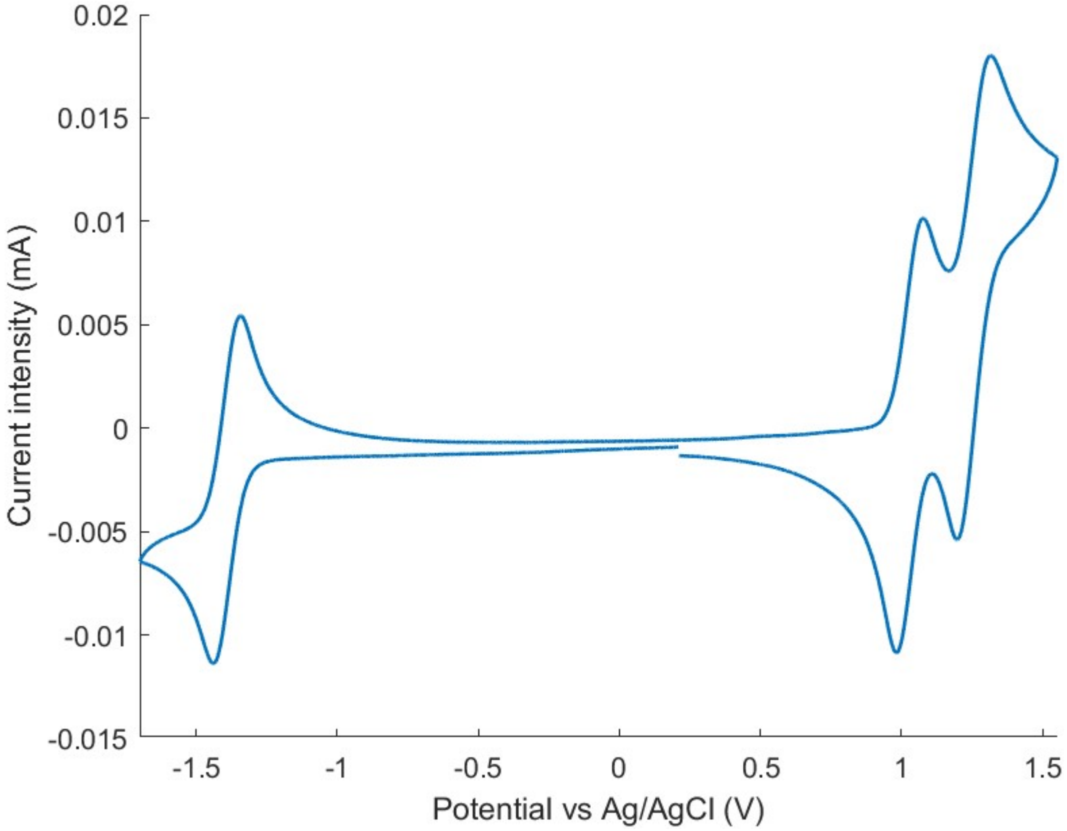

b)

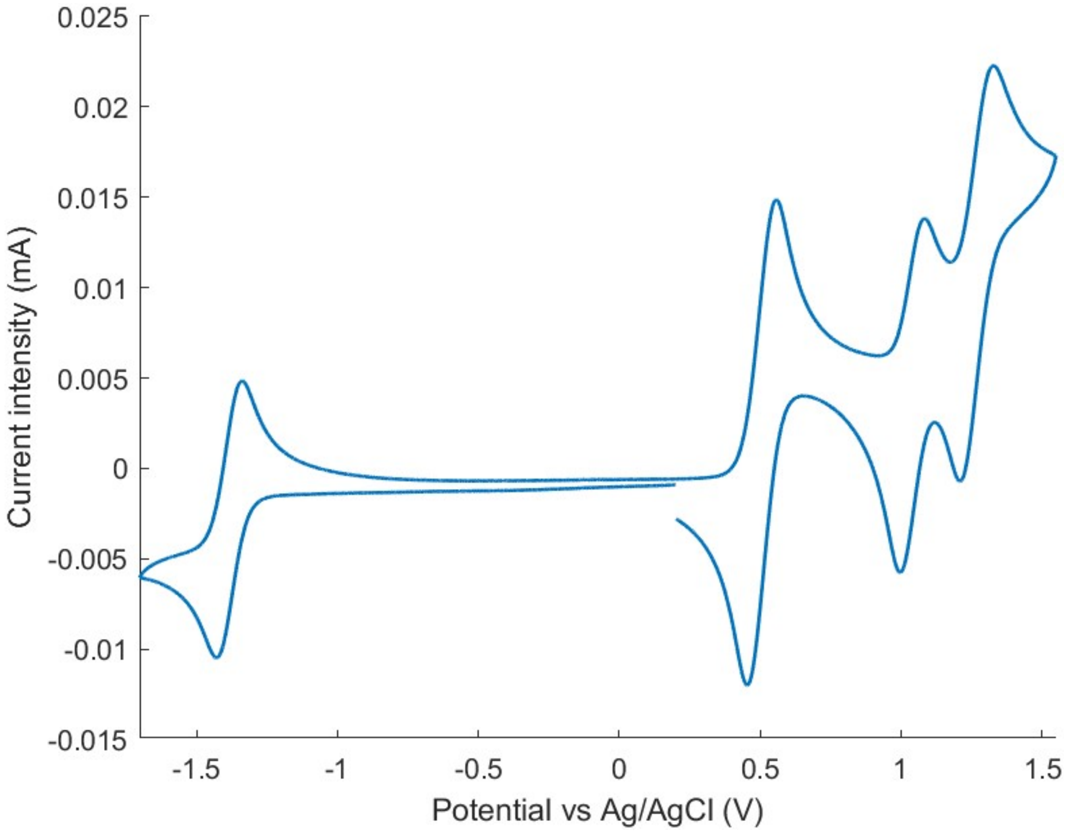

Figure S1: CV of 4DPATPN in dichloromethane (a) and of 4DPATPN in dichloromethane with Ferrocene as internal standard (b).

a)

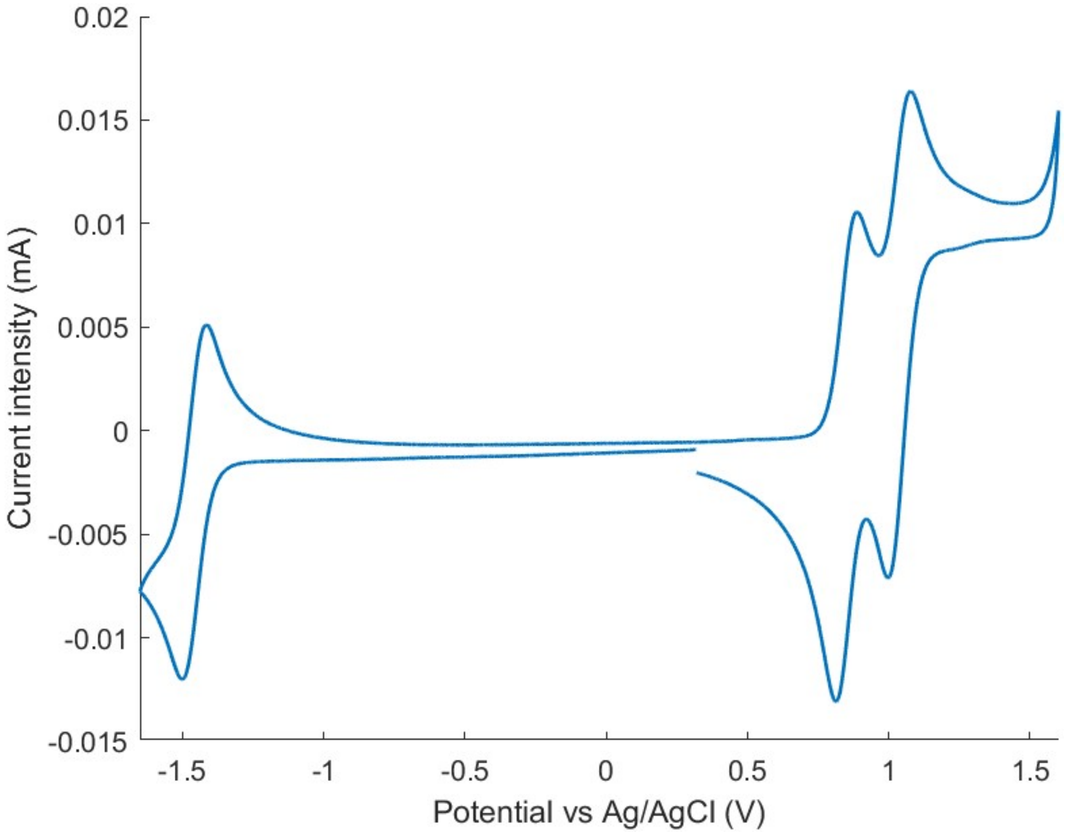

b)

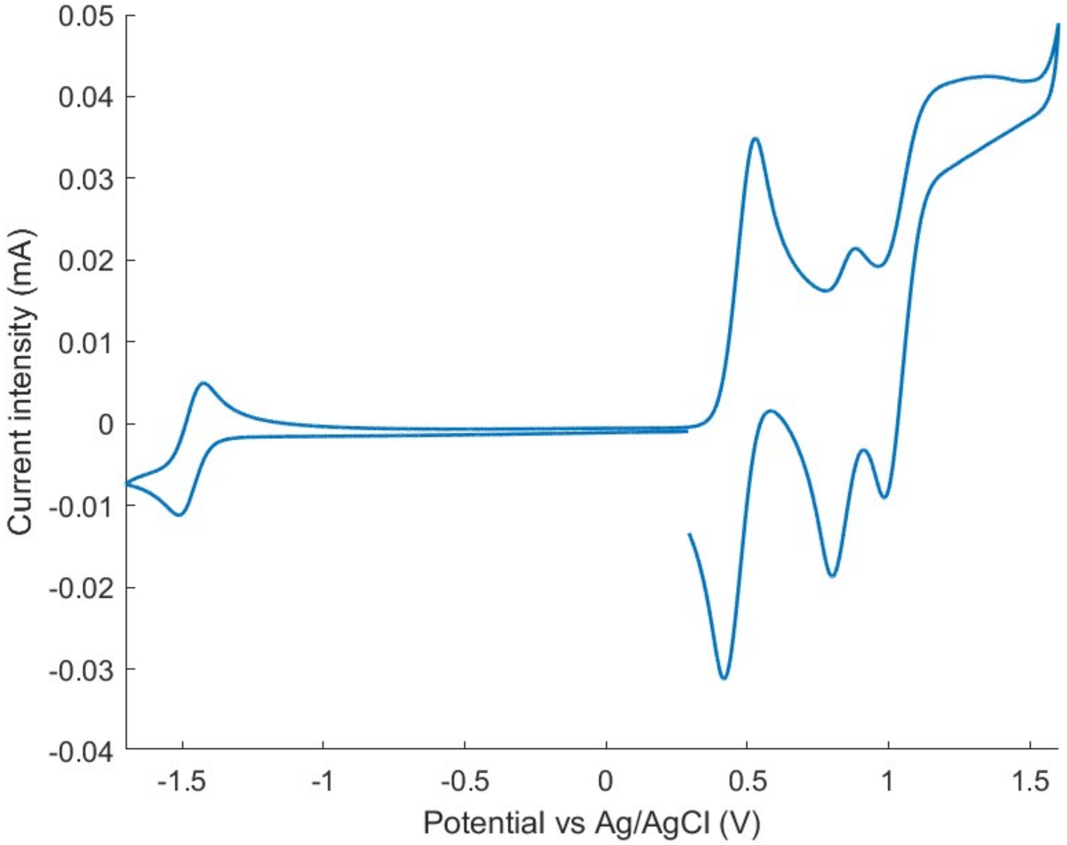

Figure S2: CV of 4DpTATPN in dichloromethane (a) and of 4DpTATPN in dichloromethane with Ferrocene as internal standard (b).

a)

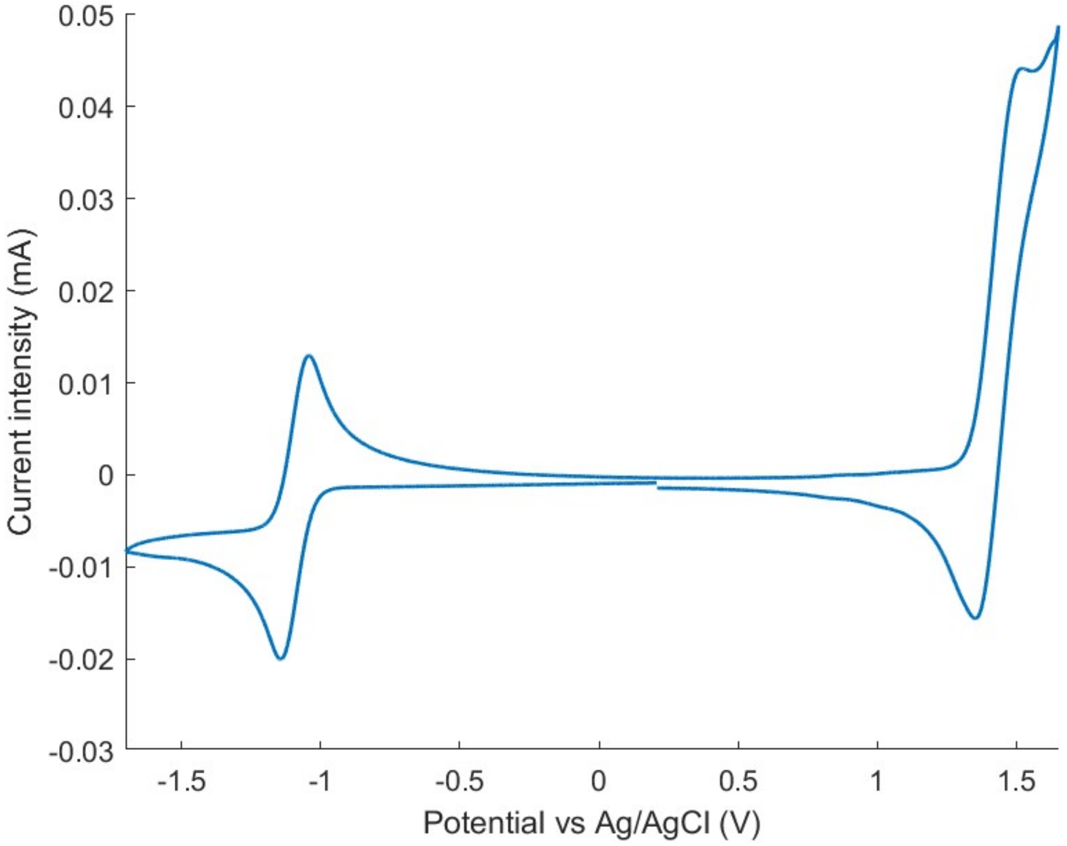

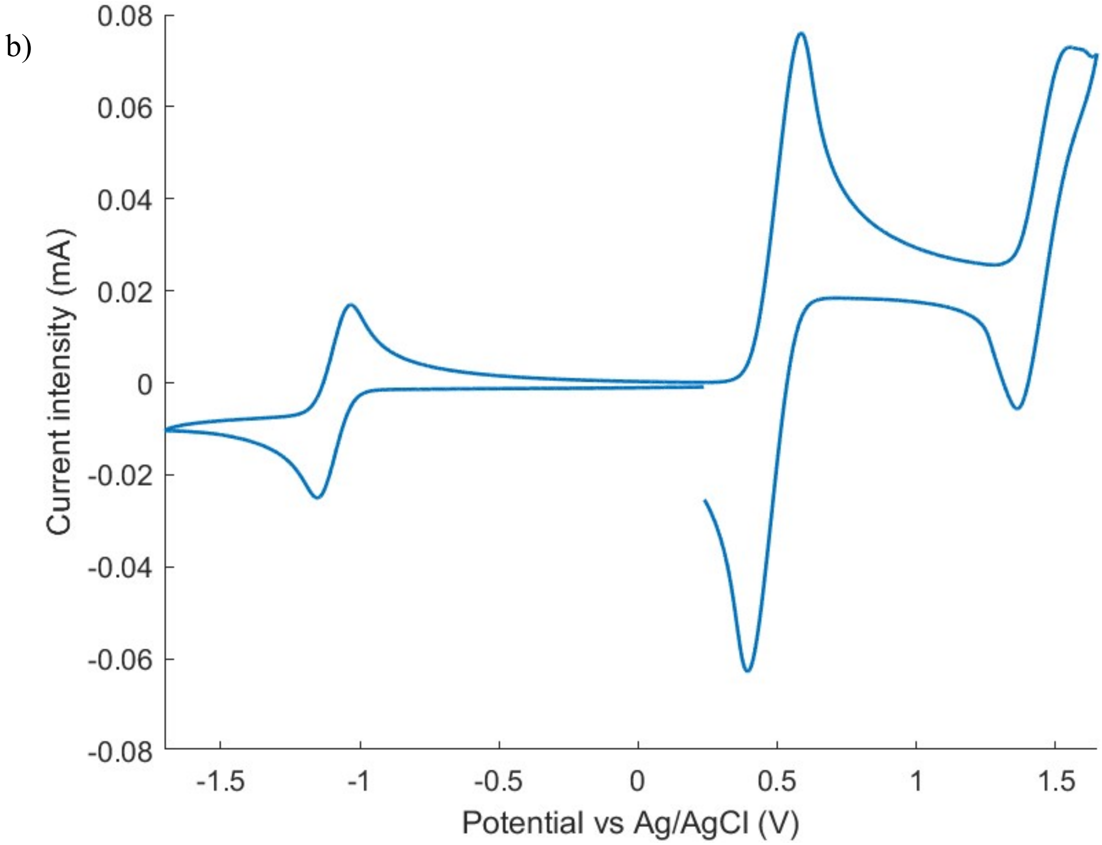

Figure S3: CV of 4^{tBu}CzTPN in dichloromethane (a) and of 4^{tBu}CzTPN in dichloromethane with Ferrocene as internal standard (b).

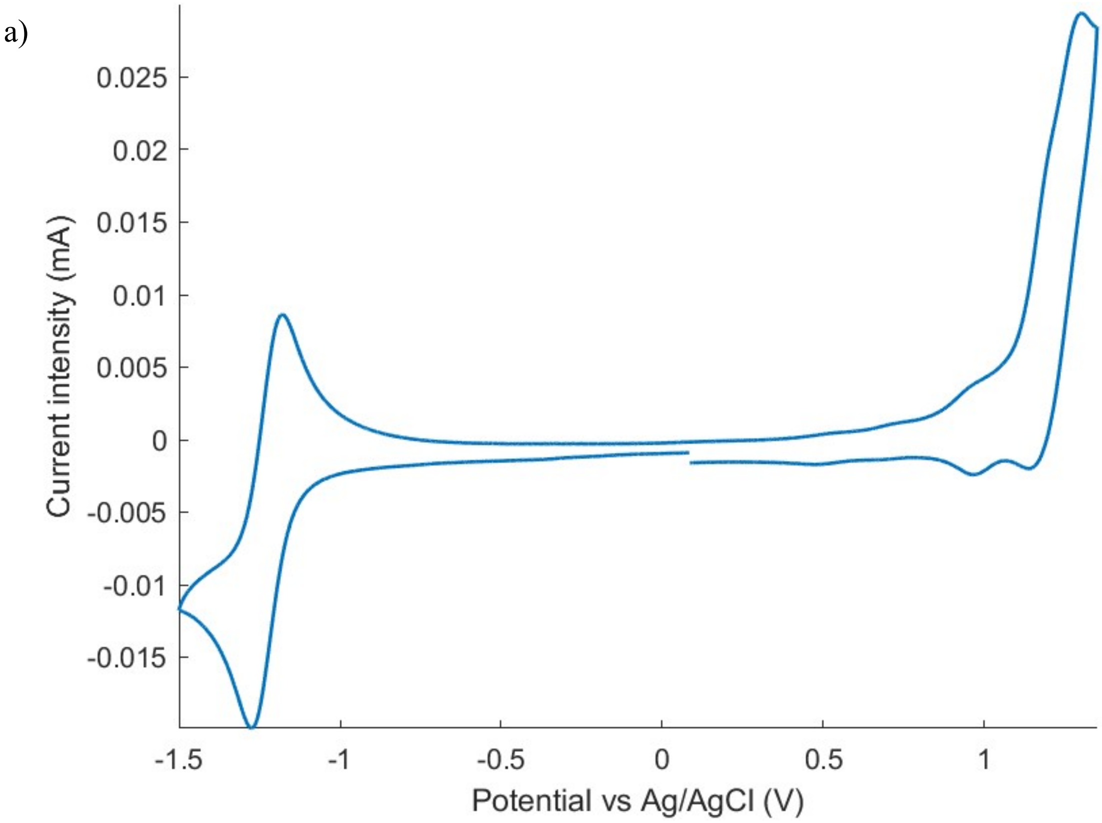

b)

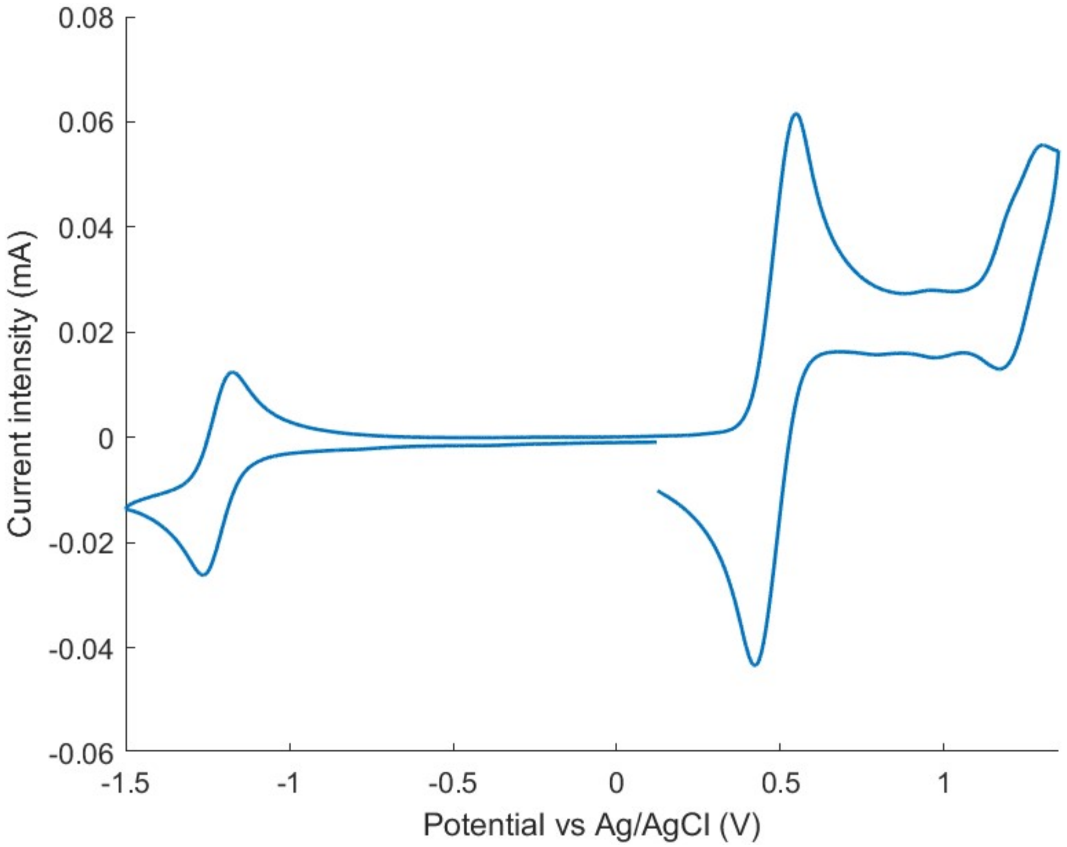

Figure S4: CV of 2DPADPPZ in dichloromethane (a) and of 2DPADPPZ in dichloromethane with Ferrocene as internal standard (b).

a)

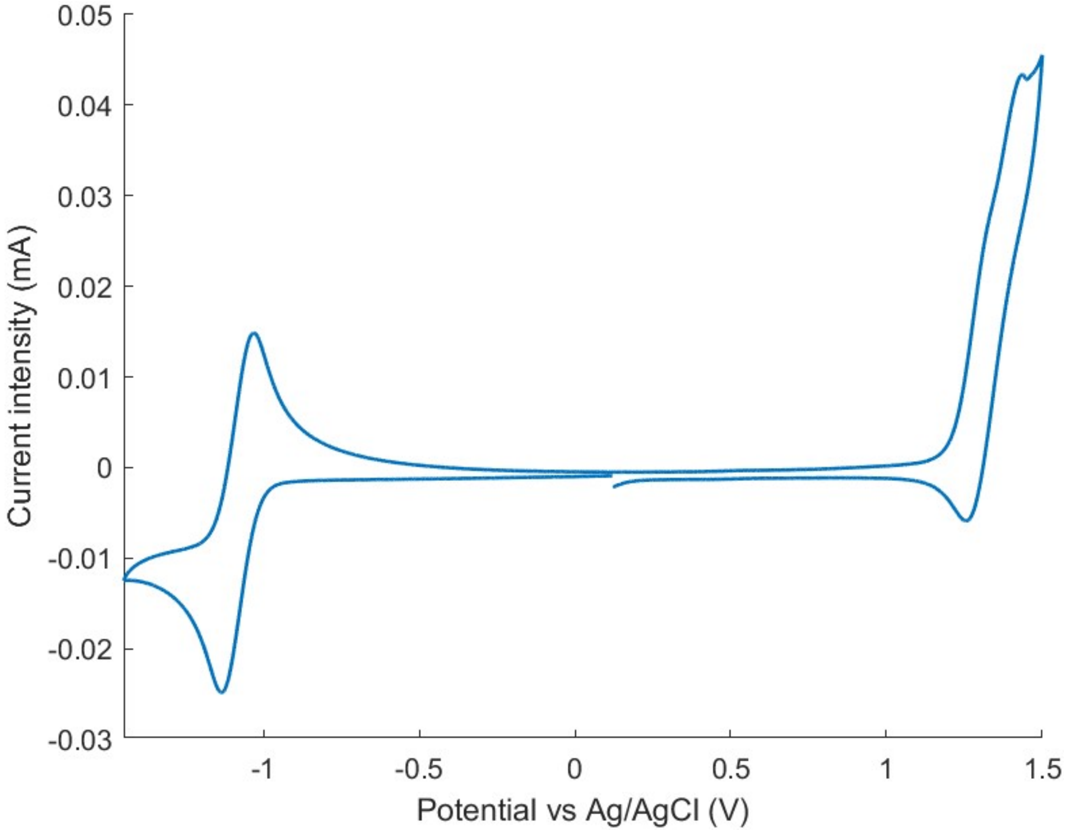

b)

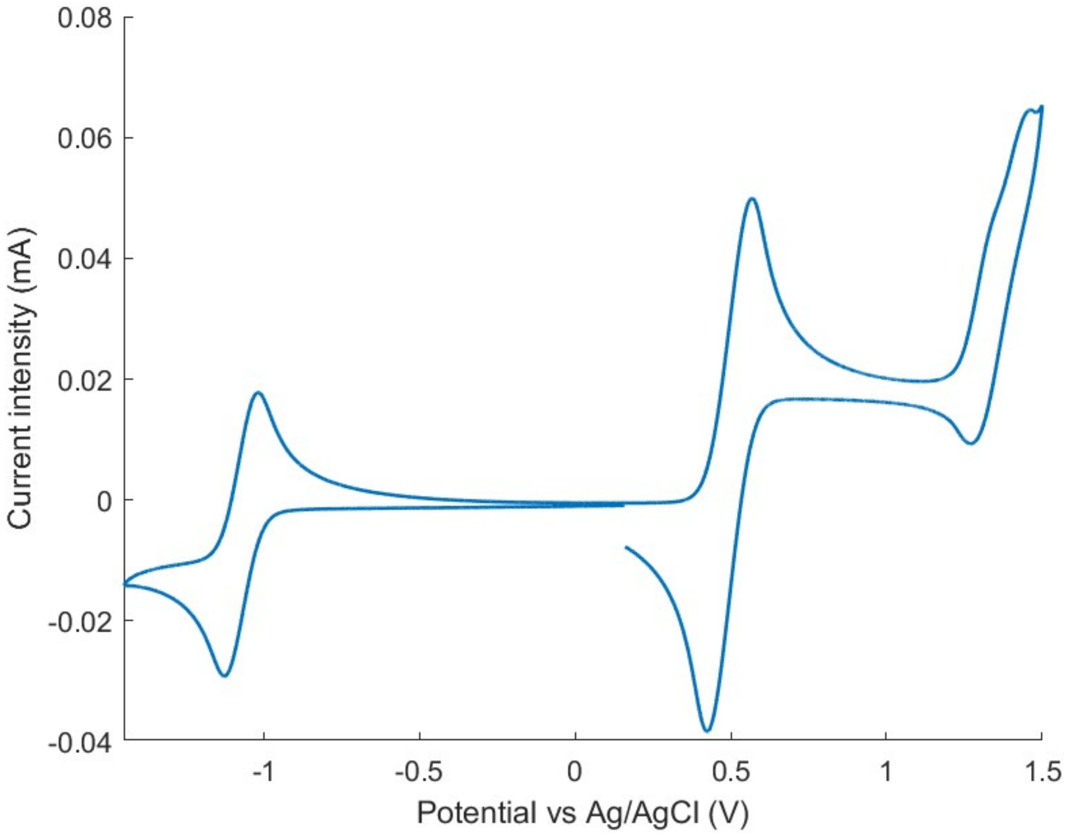

Figure S5: CV of 2^{tBu}CzDPPZ in dichloromethane (a) and of 2^{tBu}CzDPPZ in dichloromethane with Ferrocene as internal standard (b).

a)

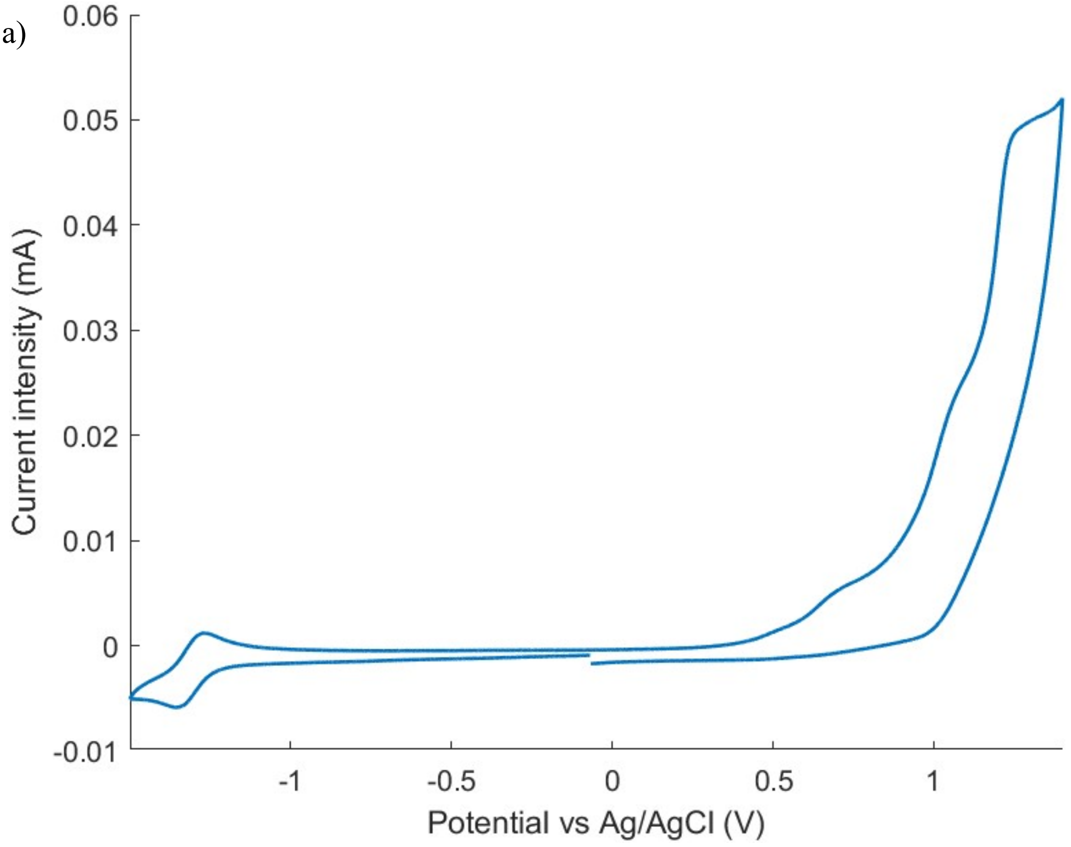

b)

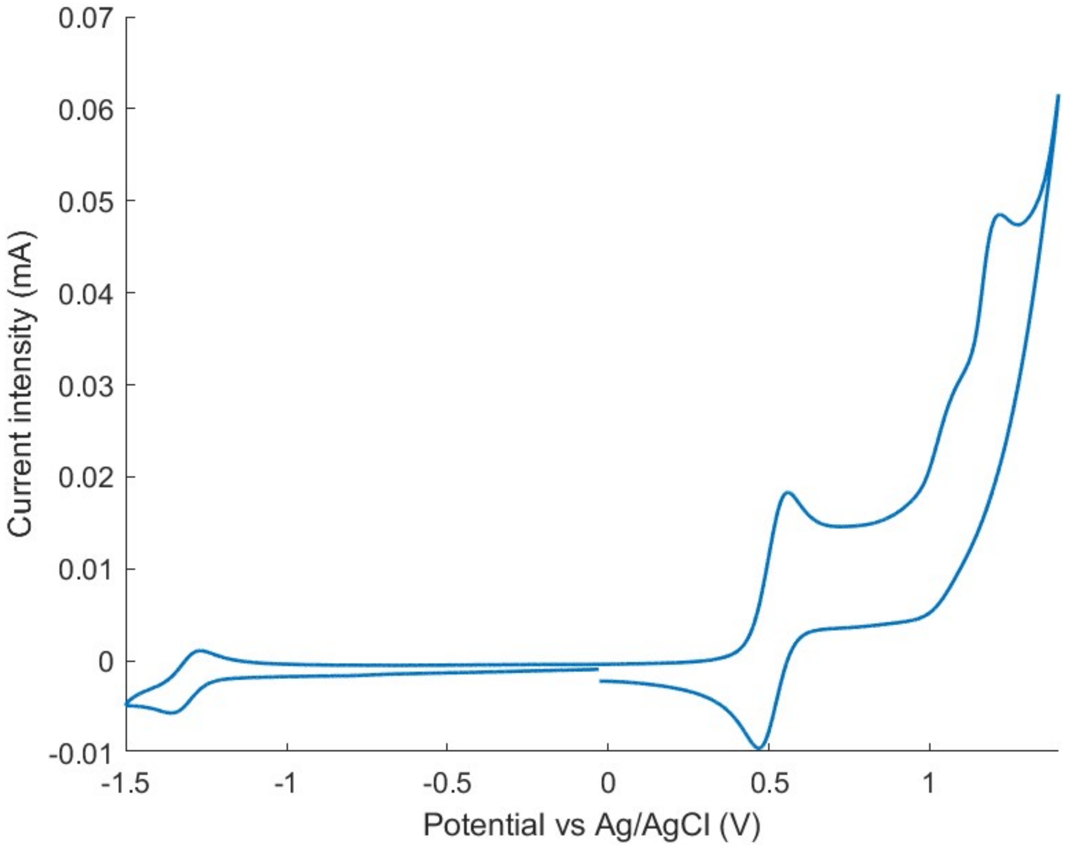

Figure S6: CV of Na_4[4DPASO_3TPN] in dimethylformamide (a) and of Na_4[4DPASO_3TPN] in dimethylformamide with Ferrocene as internal standard (b).

4. Photophysical characterization

4.1 UV-Vis absorption spectra of compounds

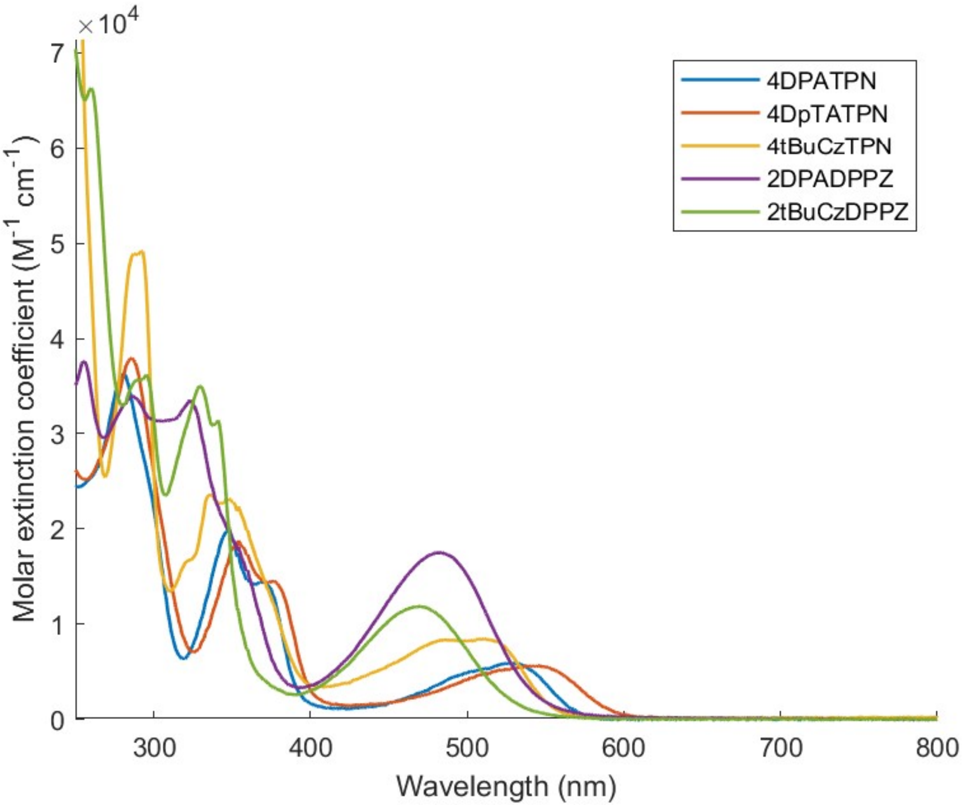

Figure S7: UV-Vis spectra of $5\cdot10^{-6}$ M dichloromethane solution.

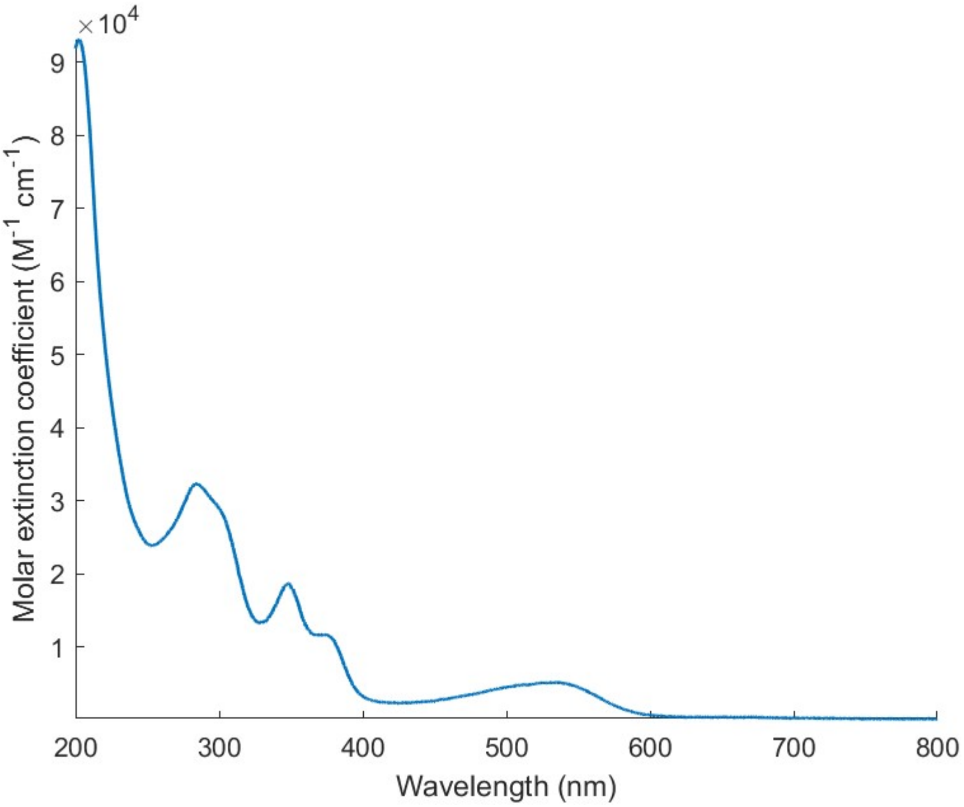

Figure S8: UV-Vis spectra of $5\cdot10^{-6}$ M of $Na_4[4DPASO_3TPN]$ in water solution.

4.2 Emission spectra in solution

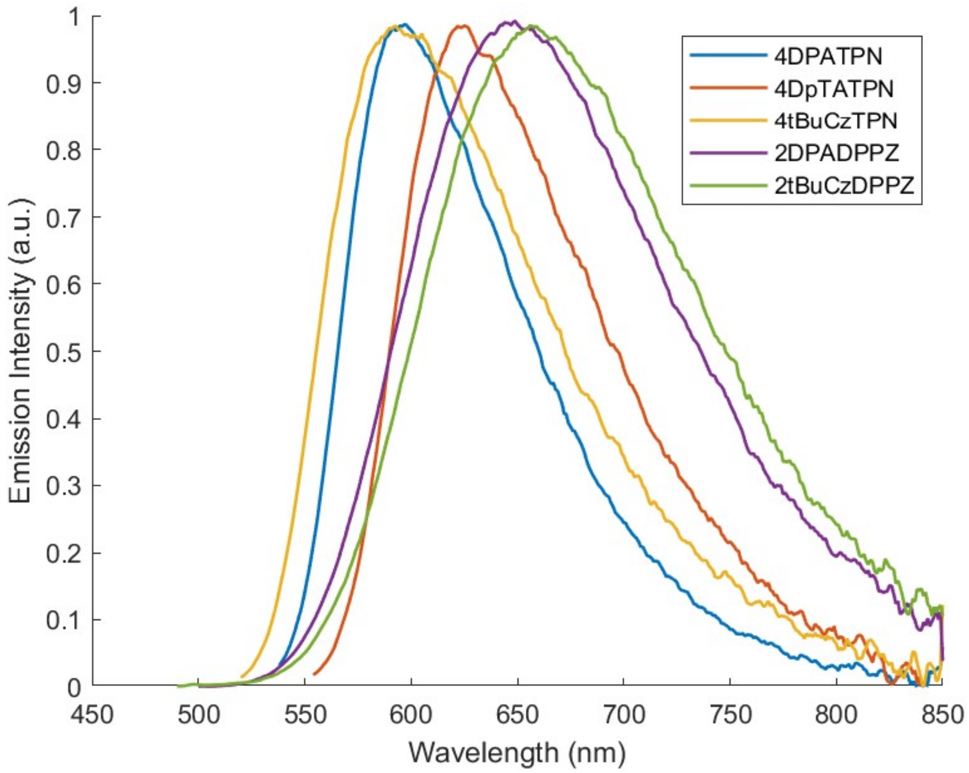

Figure S9: Normalized PL spectra of dichloromethane solutions.

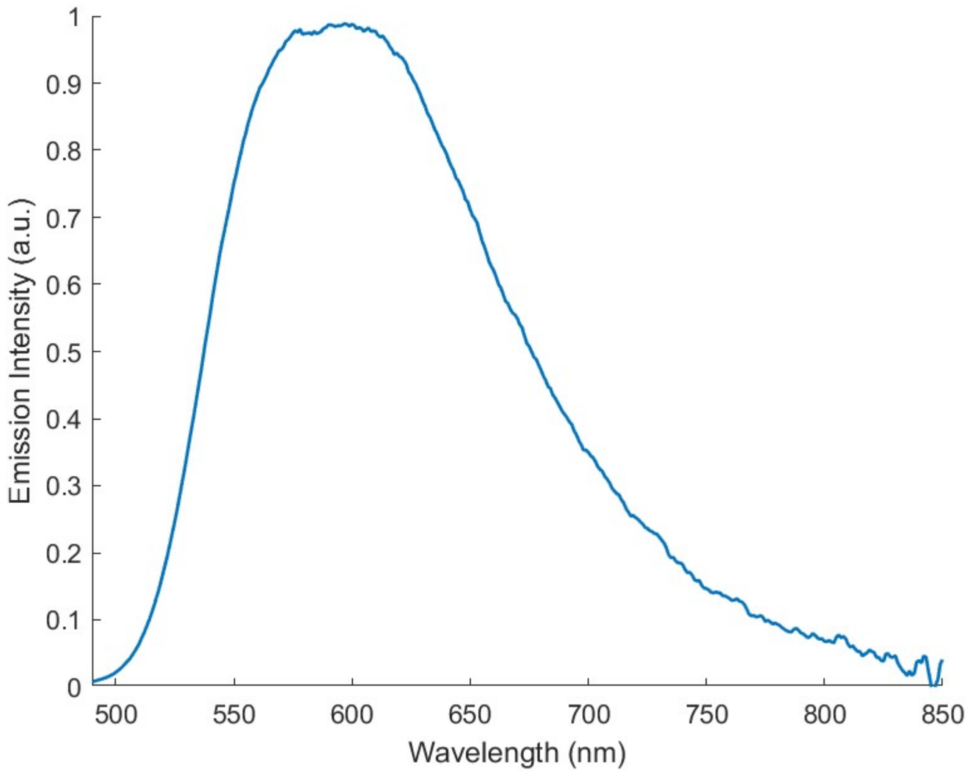

Figure S10: Normalized PL spectra of $Na_4[4DPASO_3TPN]$ in water solution.

4.3 Absorption-excitation spectra comparisons.

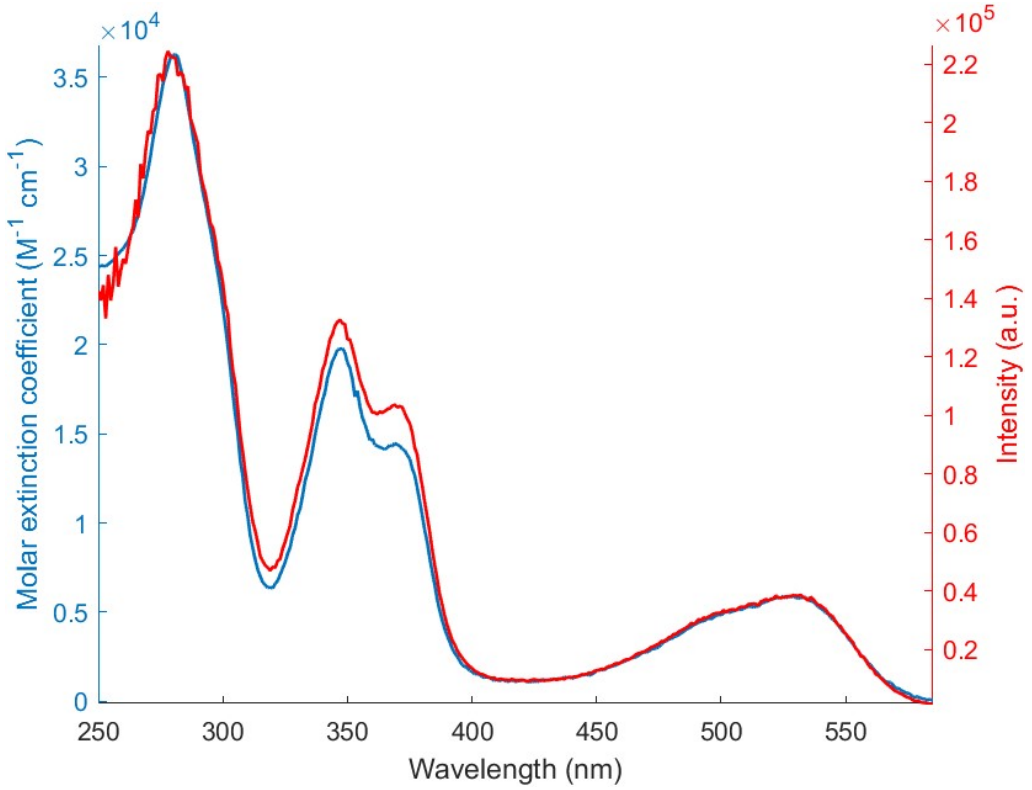

Figure S11: Absorption (blue curve) and excitation (red curve) spectra of 4DPATPN in dichloromethane.

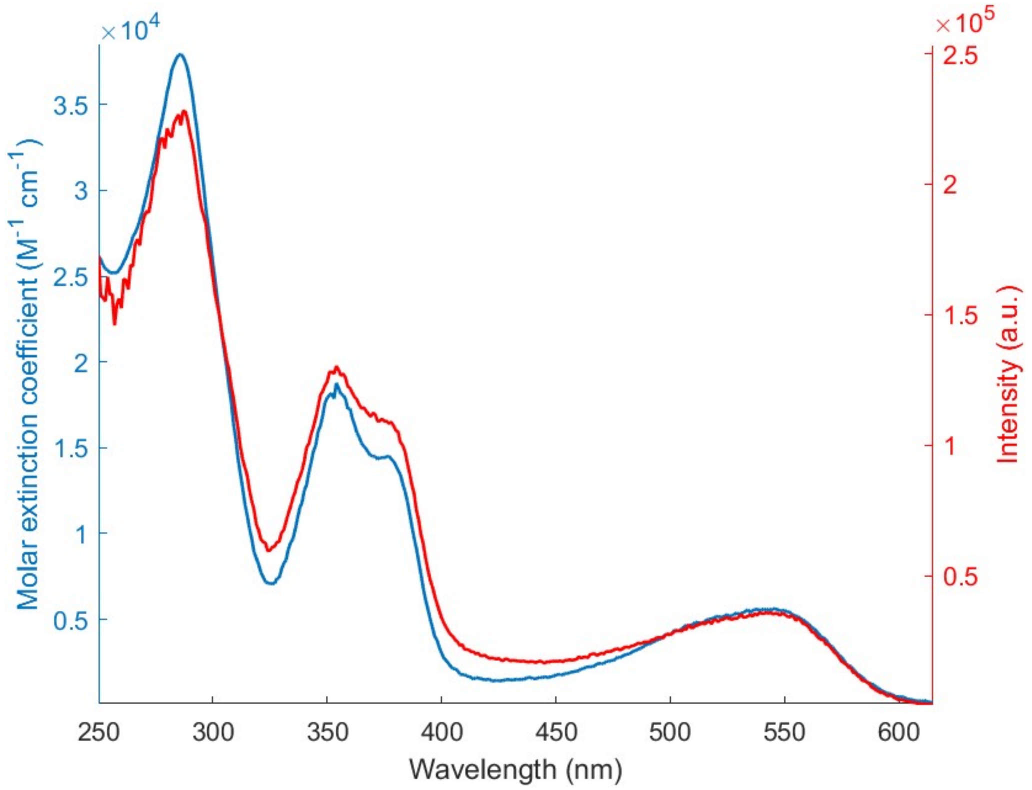

Figure S12: Absorption (blue curve) and excitation (red curve) spectra of 4DpTATPN in dichloromethane.

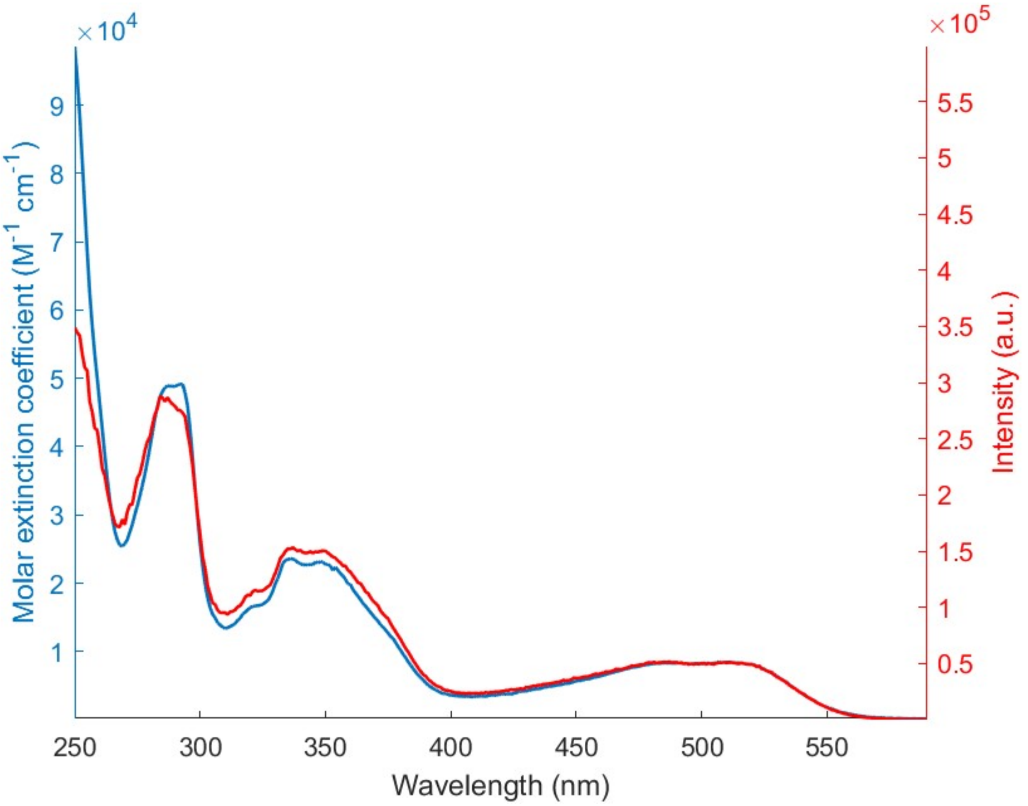

Figure S13: Absorption (blue curve) and excitation (red curve) spectra of 4^{tBu}CzTPN in dichloromethane.

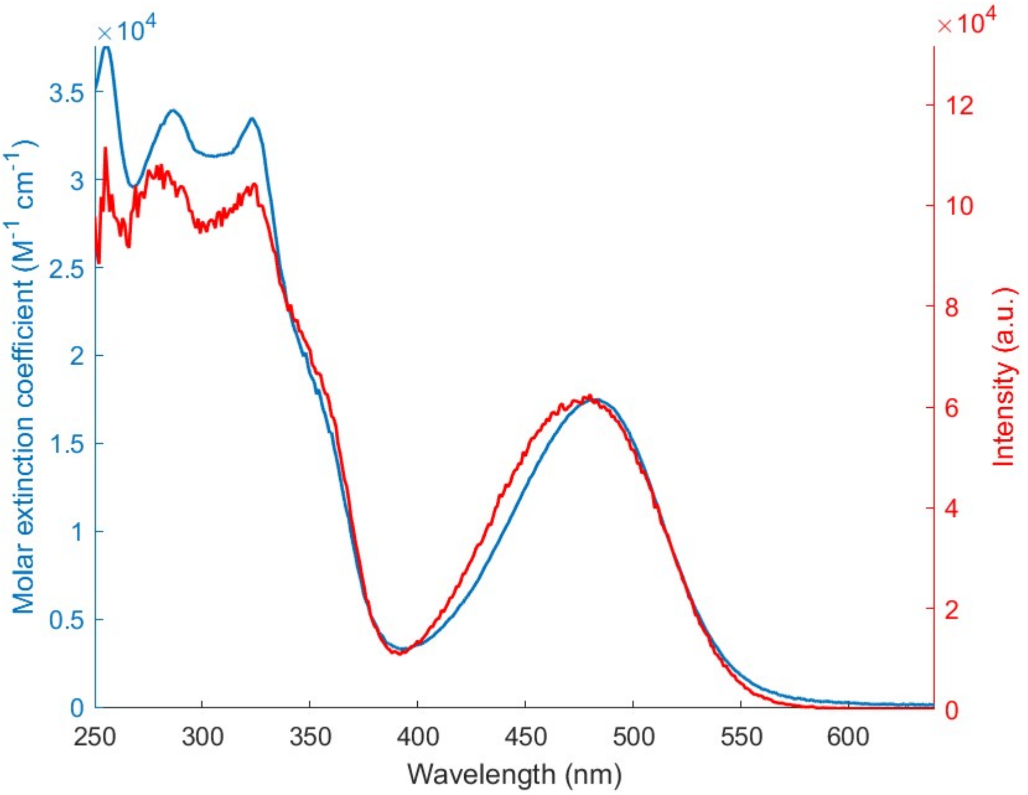

Figure S14: Absorption (blue curve) and excitation (red curve) spectra of 2DPADPPZ in dichloromethane.

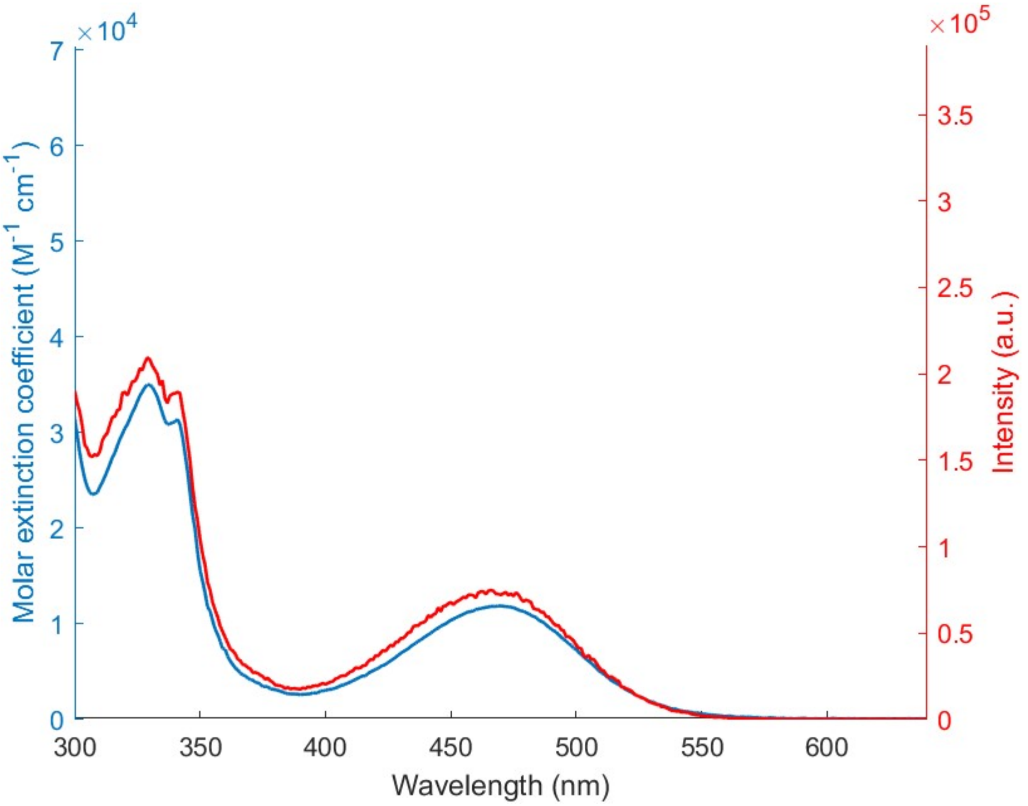

Figure S15: Absorption (blue curve) and excitation (red curve) spectra of 2^{tBu}CzDPPZ in dichloromethane.

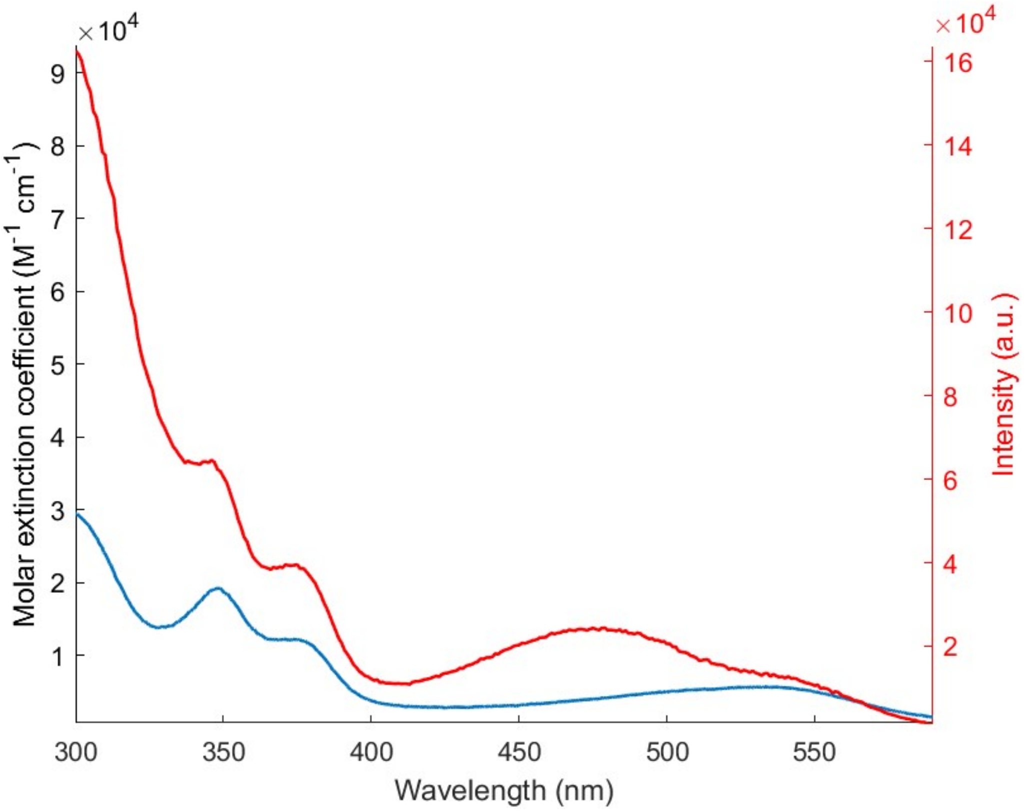

Figure S16: Absorption (blue curve) and excitation (red curve) spectra of Na_4[4DPASO_3TPN] in water.

4.4 Luminescent decay curves

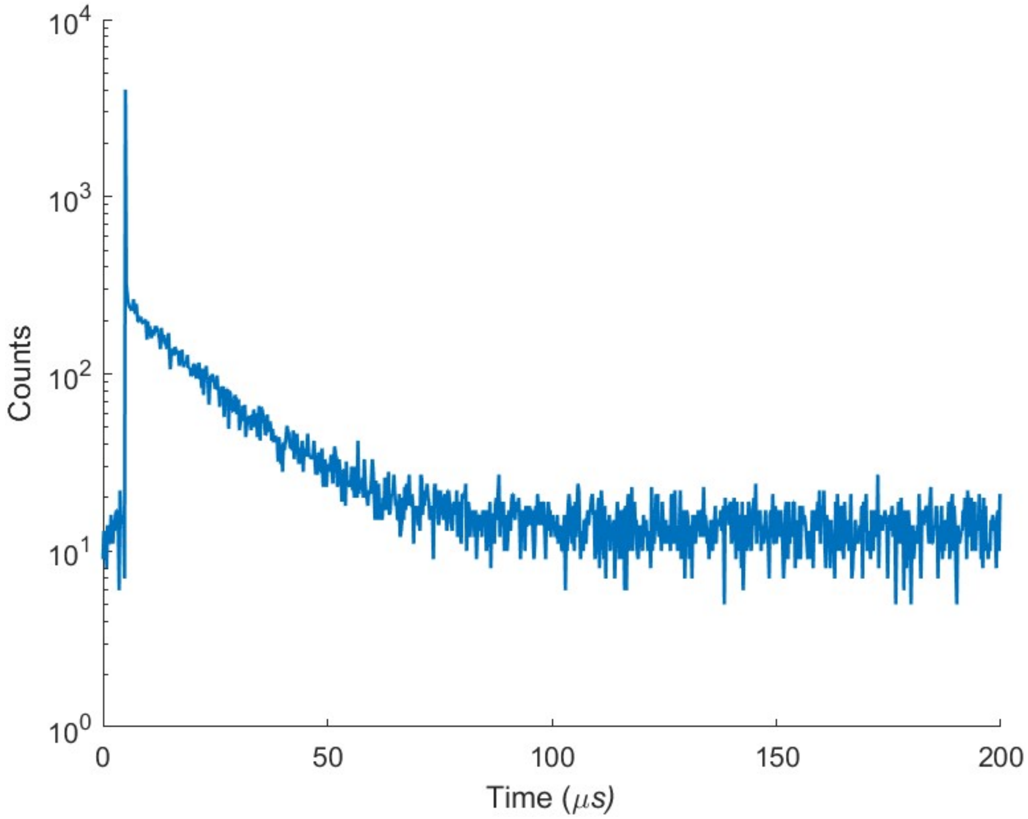

Figure S17: Luminescence decay curve of 4DPATPN in dichloromethane.

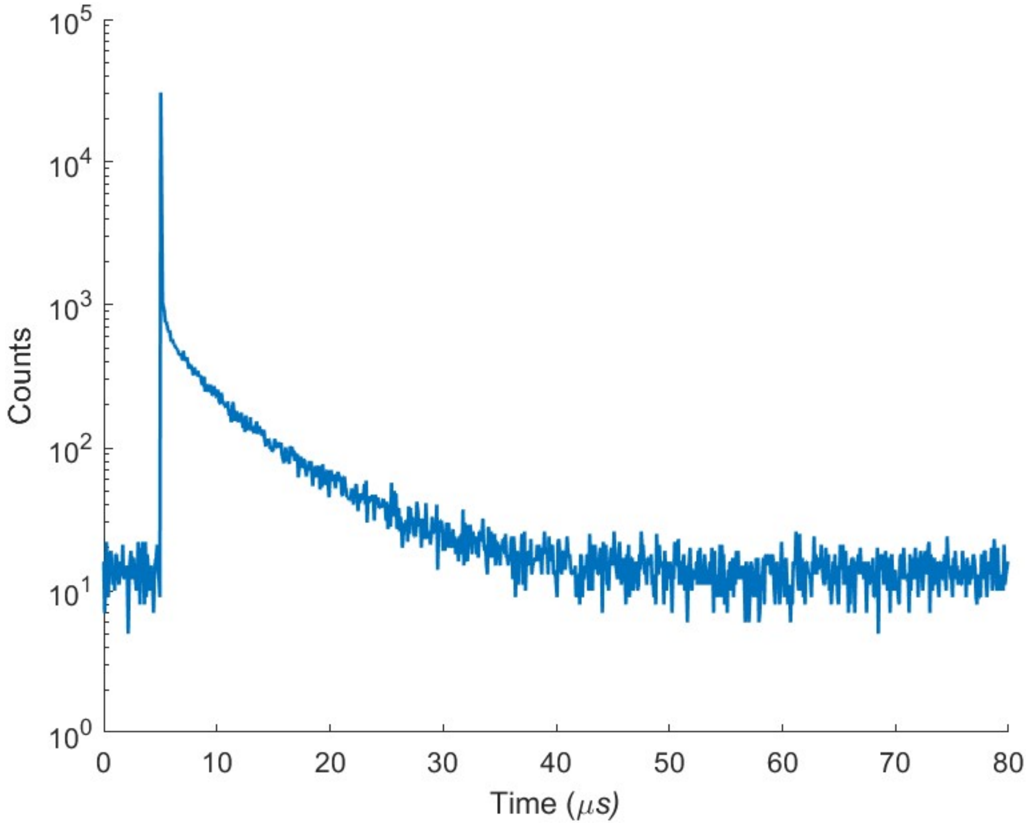

Figure S18: Luminescence decay curve of 4DpTATPN in dichloromethane.

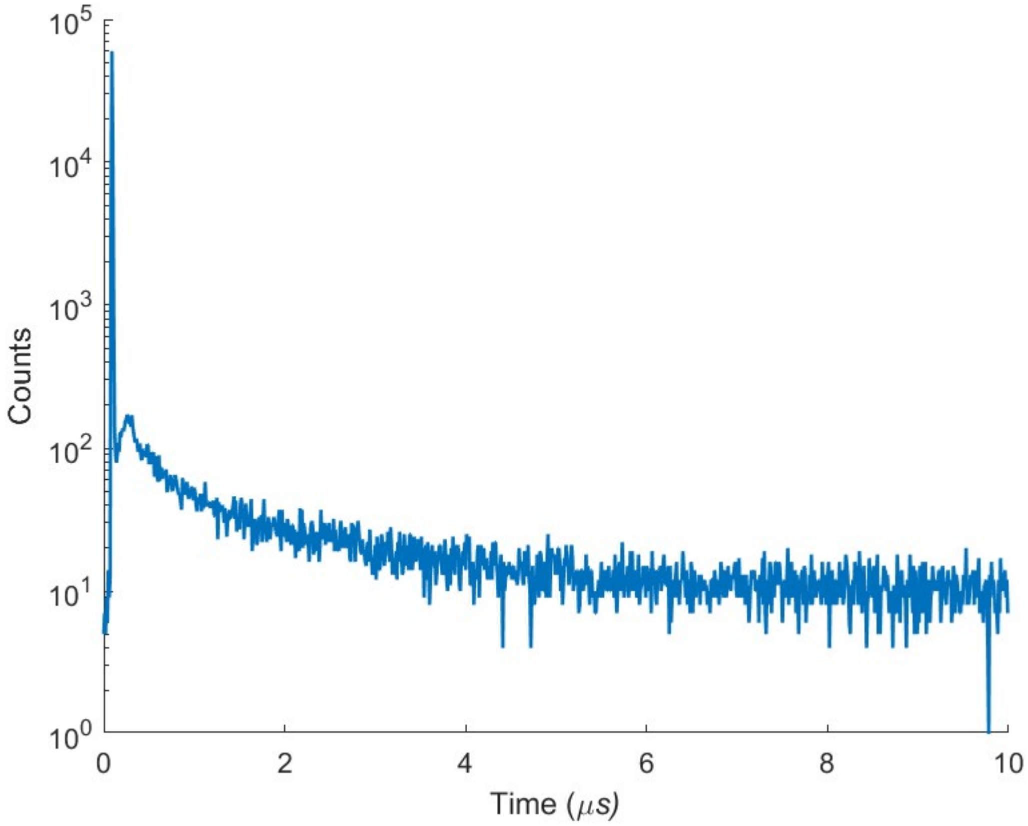

Figure S19: Luminescence decay curve of 4^{tBu}CzTPN in dichloromethane.

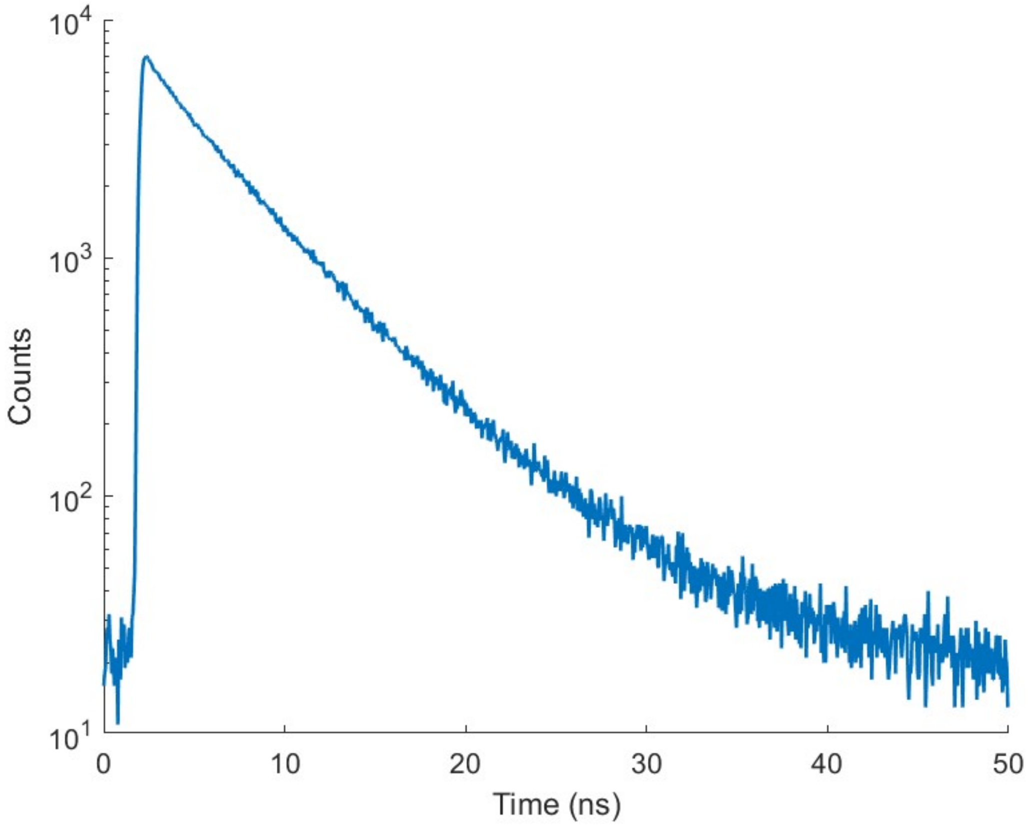

Figure S20: Luminescence decay curve of 2DPADPPZ in dichloromethane.

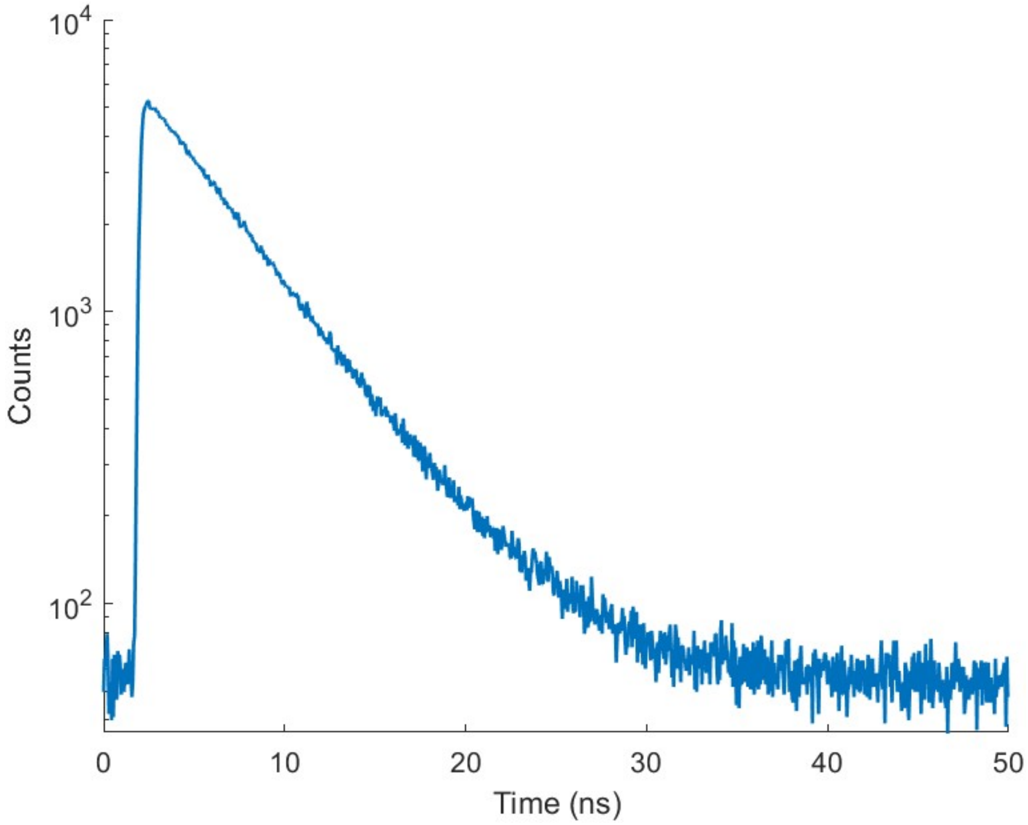

Figure S21: Luminescence decay curve of 2^{tBu}CzDPPZ in dichloromethane.

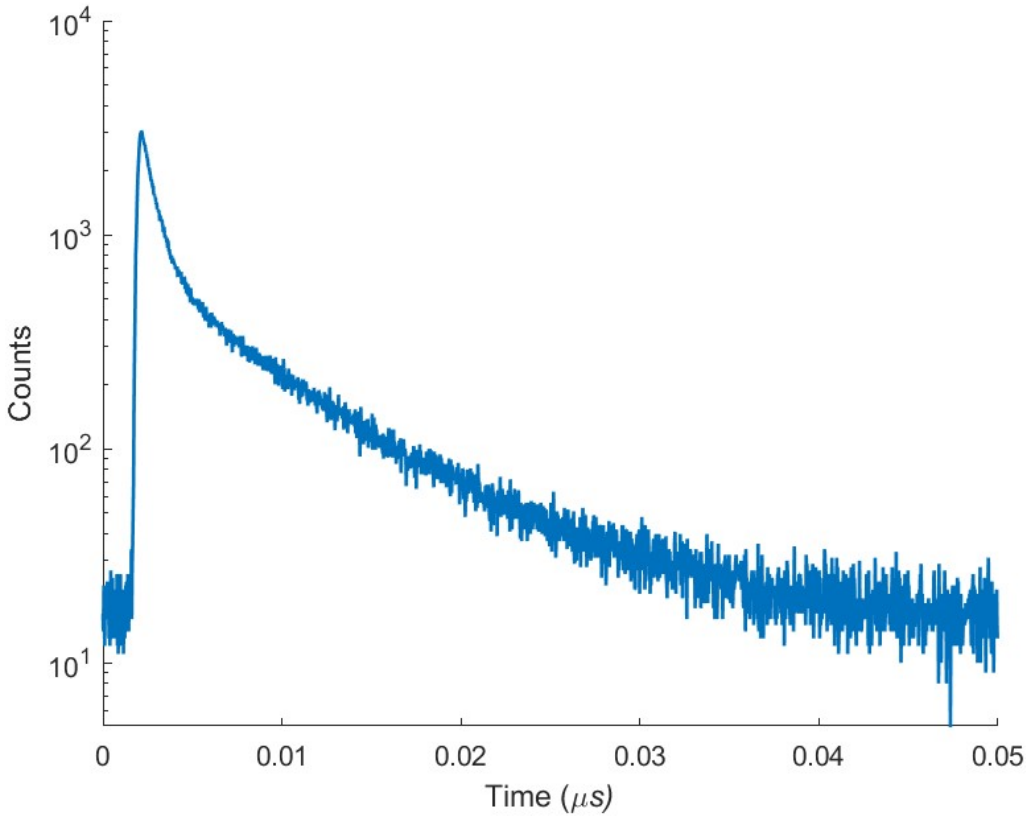

Figure S22: Luminescence decay curve of Na_4[4DPASO_3TPN] in water.

5. Electrochemiluminescence characterization

5.1 ECL-voltage curves.

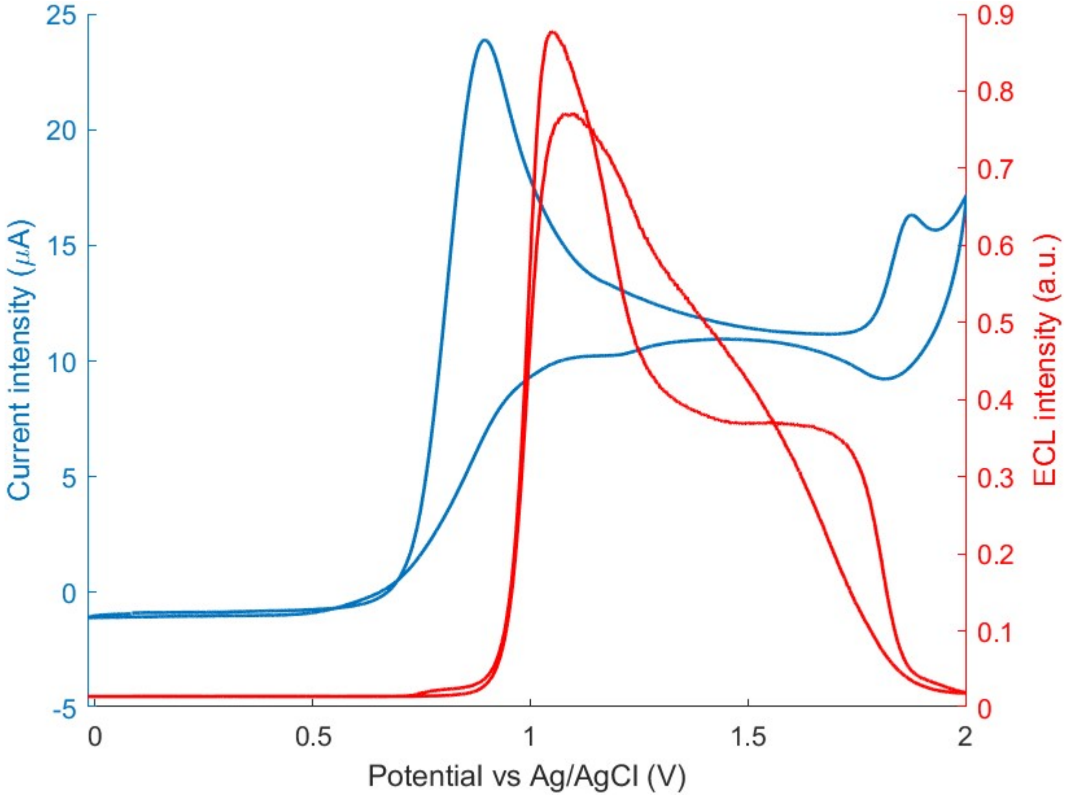

Figure S23: ECL-voltage curves of 4DPATPN in dichloromethane.

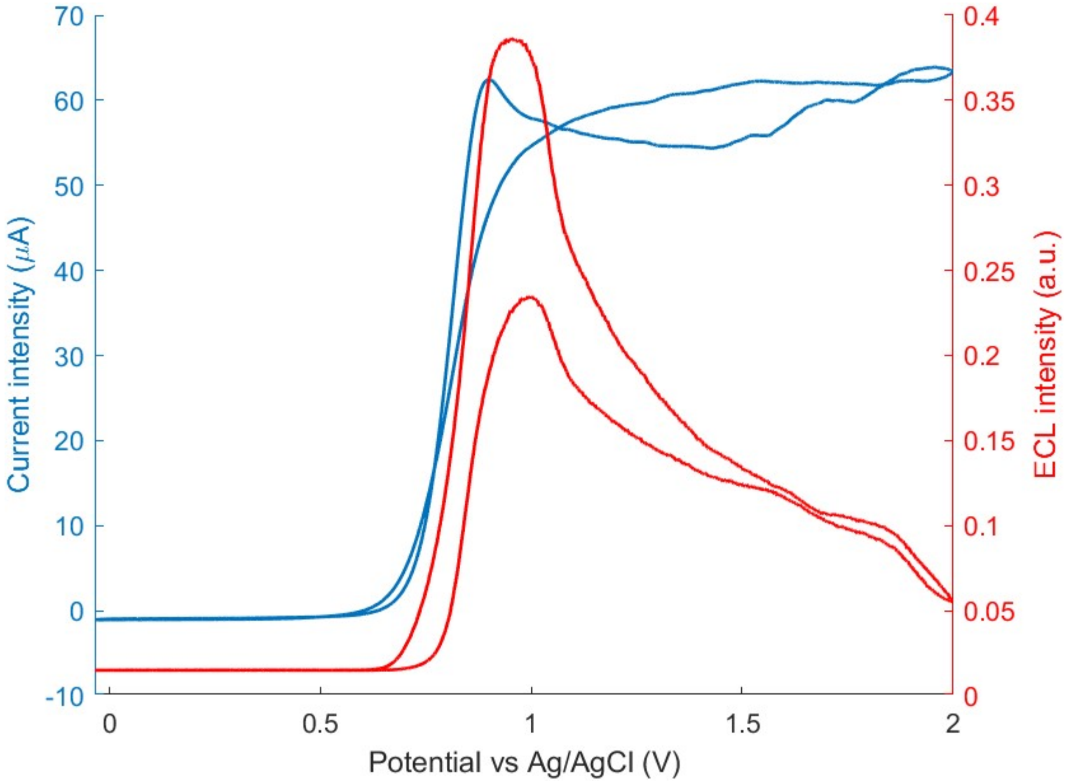

Figure S24: ECL-voltage curves of 4DpTATPN in dichloromethane.

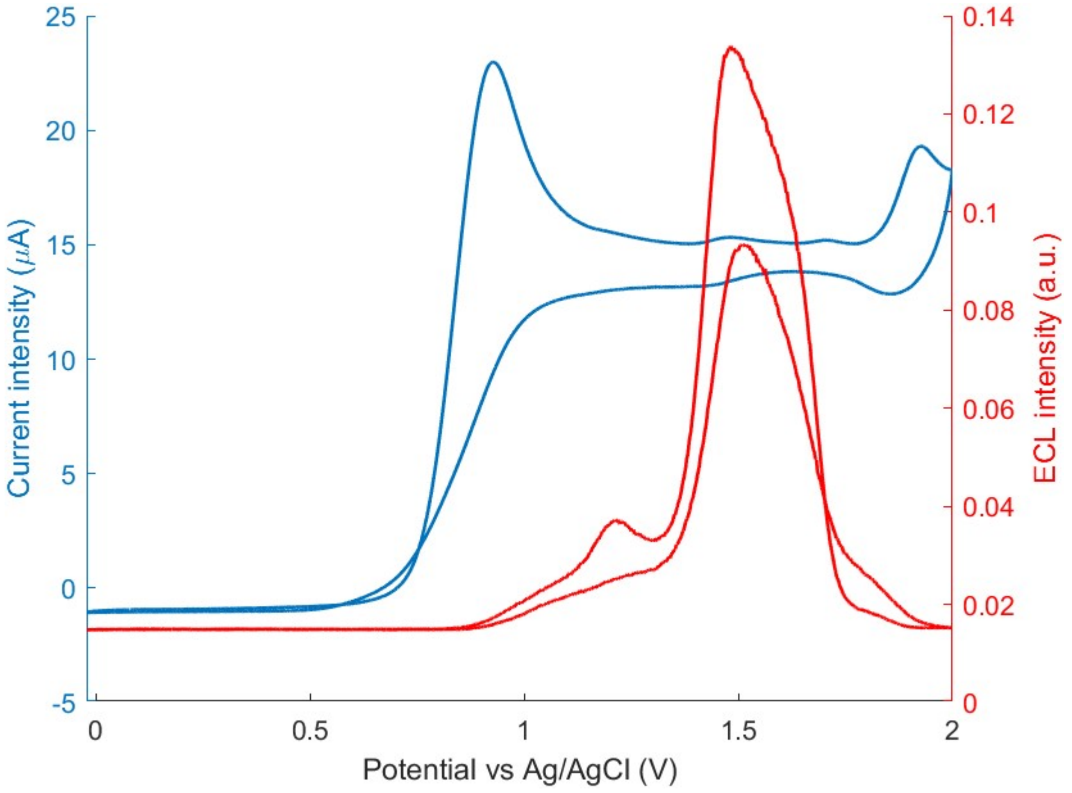

Figure S25: ECL-voltage curves of 4^{tBu}CzTPN in dichloromethane.

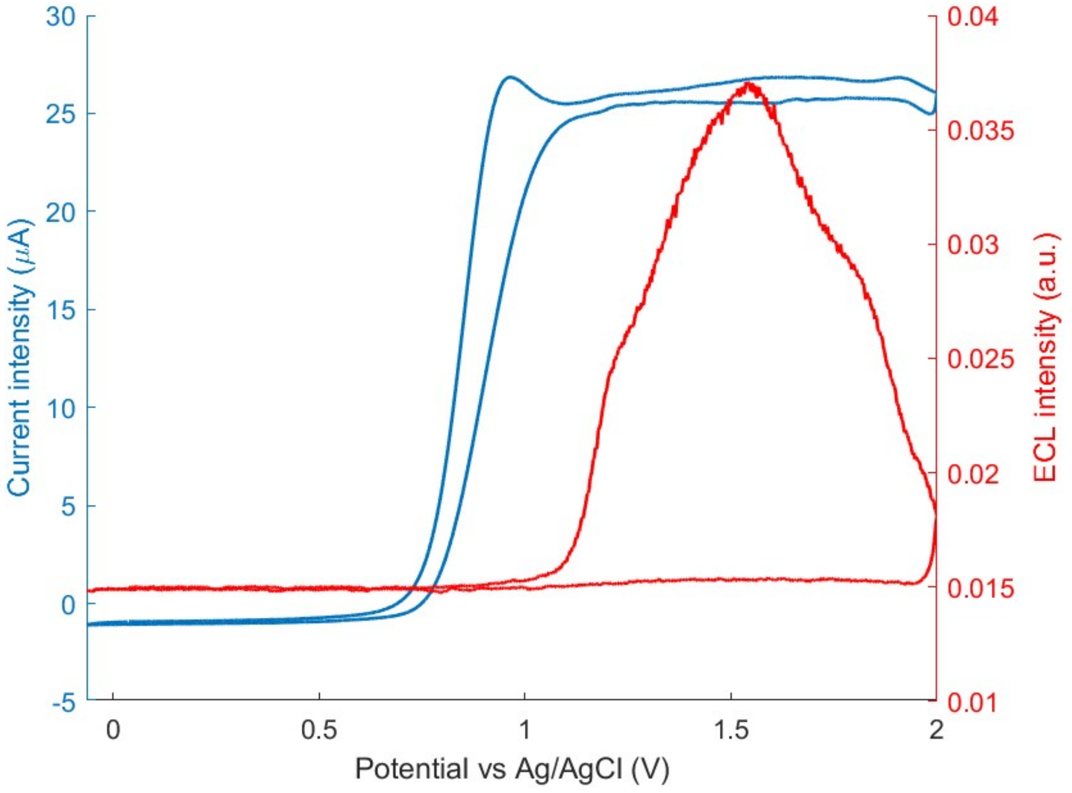

Figure S26: ECL-voltage curves of 2DPADPPZ in dichloromethane.

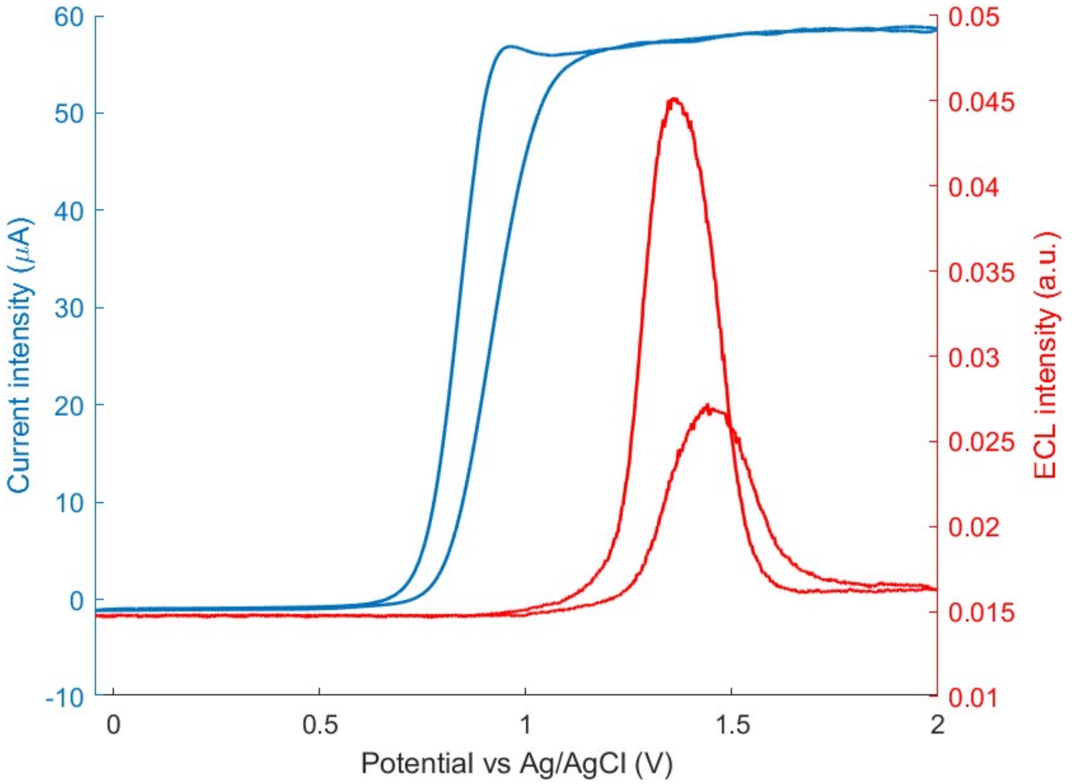

Figure S27: ECL-voltage curves of 2^{tBu}CzDPPZ in dichloromethane.

5.2 ECL-PL spectra comparisons

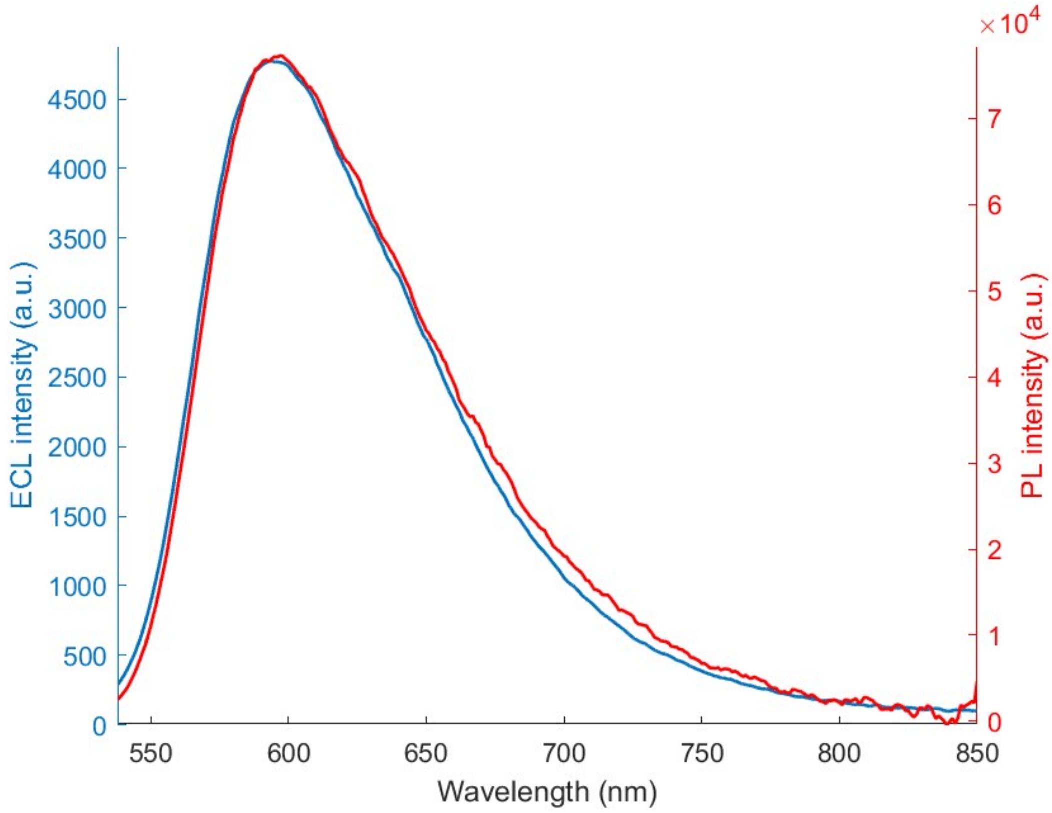

Figure S28: ECL (blue curve) and PL (red curve) spectra of 4DPATPN in dichloromethane.

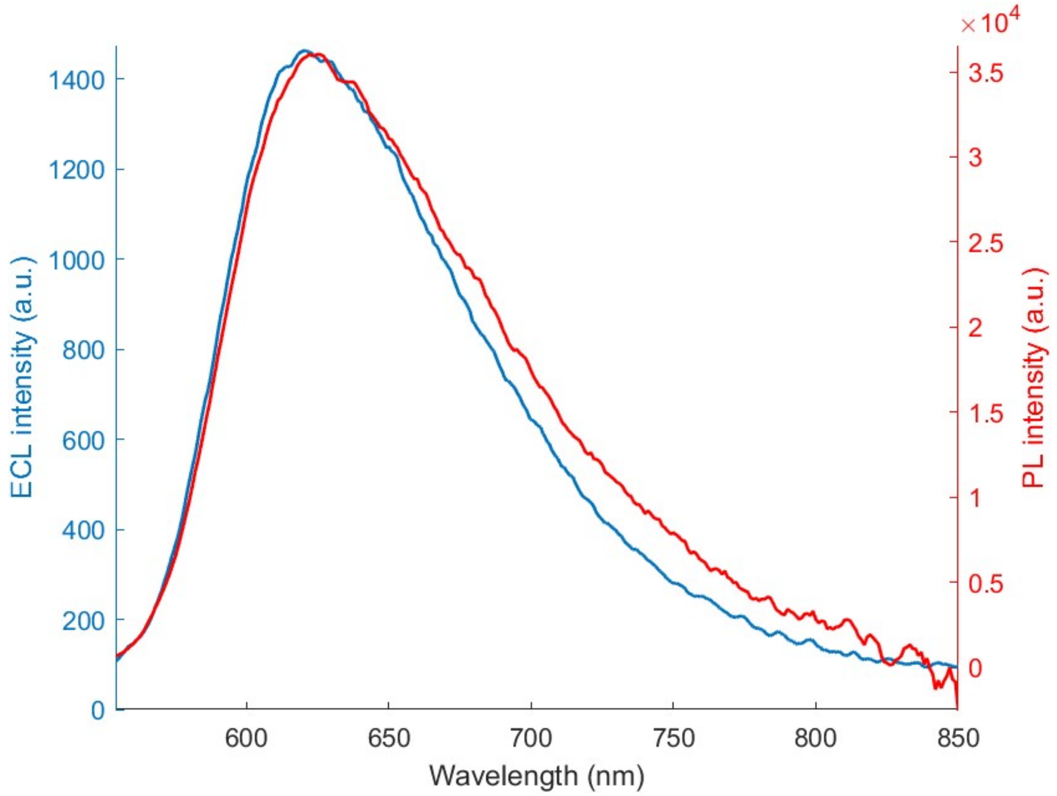

Figure S29: ECL (blue curve) and PL (red curve) spectra of 4DpTATPN in dichloromethane.

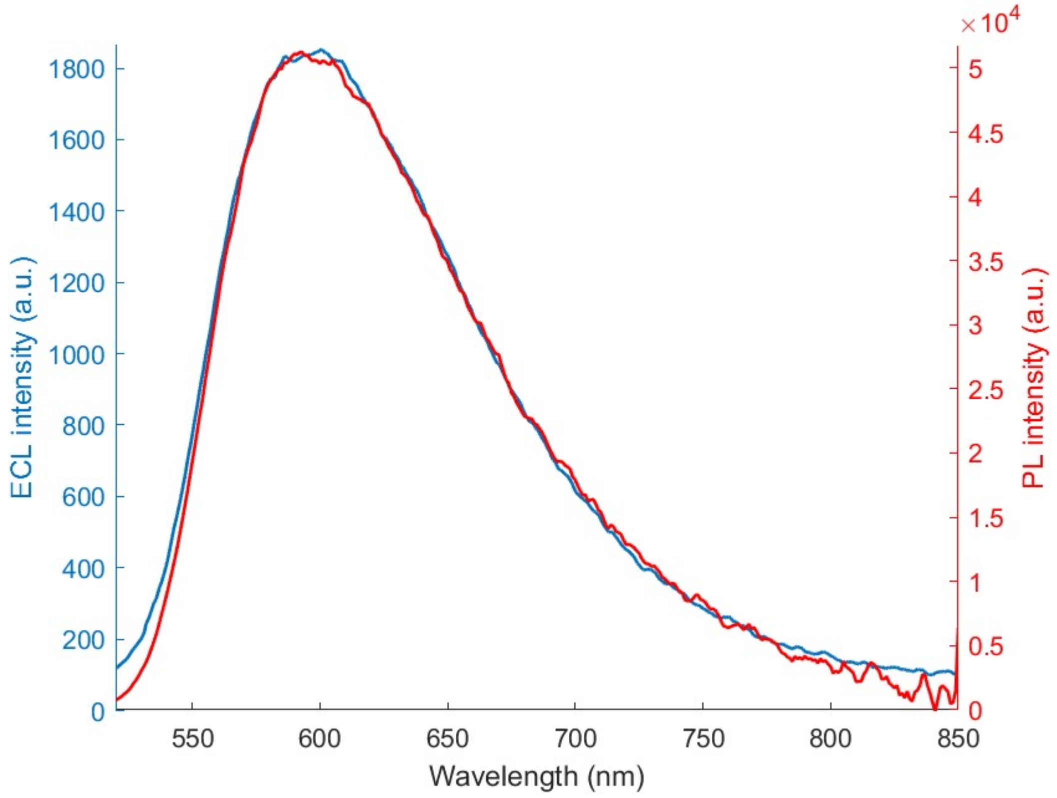

Figure S30: ECL (blue curve) and PL (red curve) spectra of 4^{tBu}CzTPN in dichloromethane.

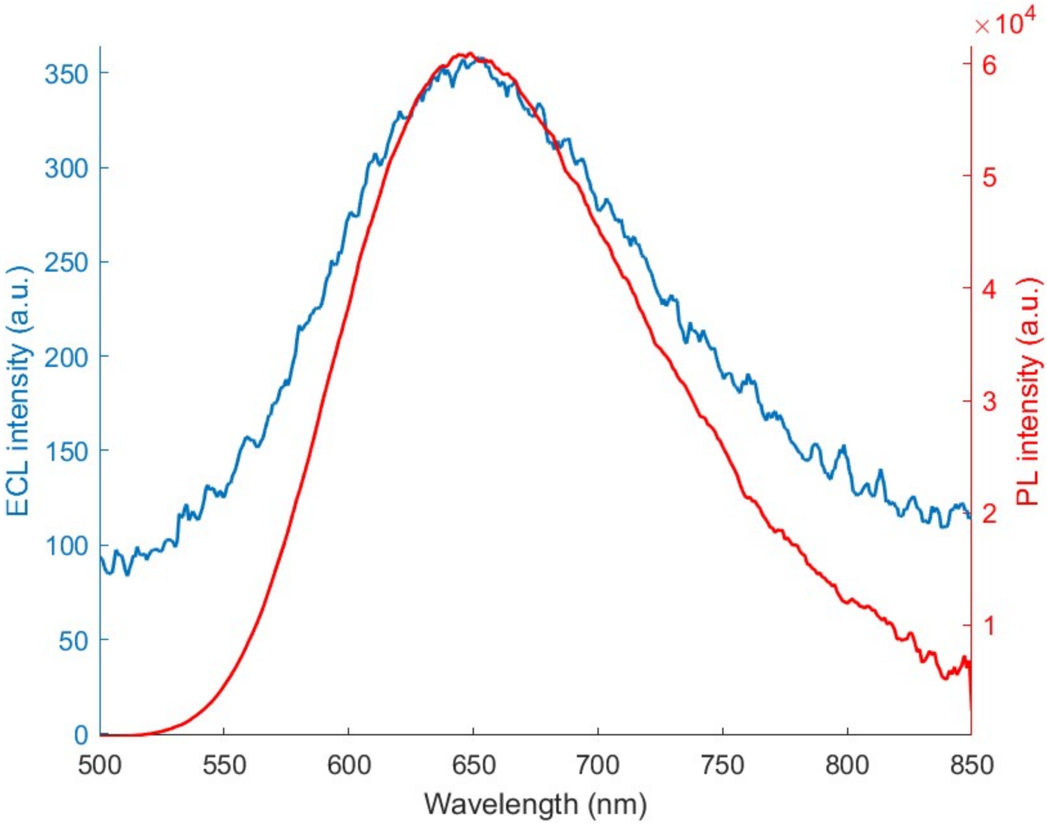

Figure S31: ECL (blue curve) and PL (red curve) spectra of 2DPADPPZ in dichloromethane.

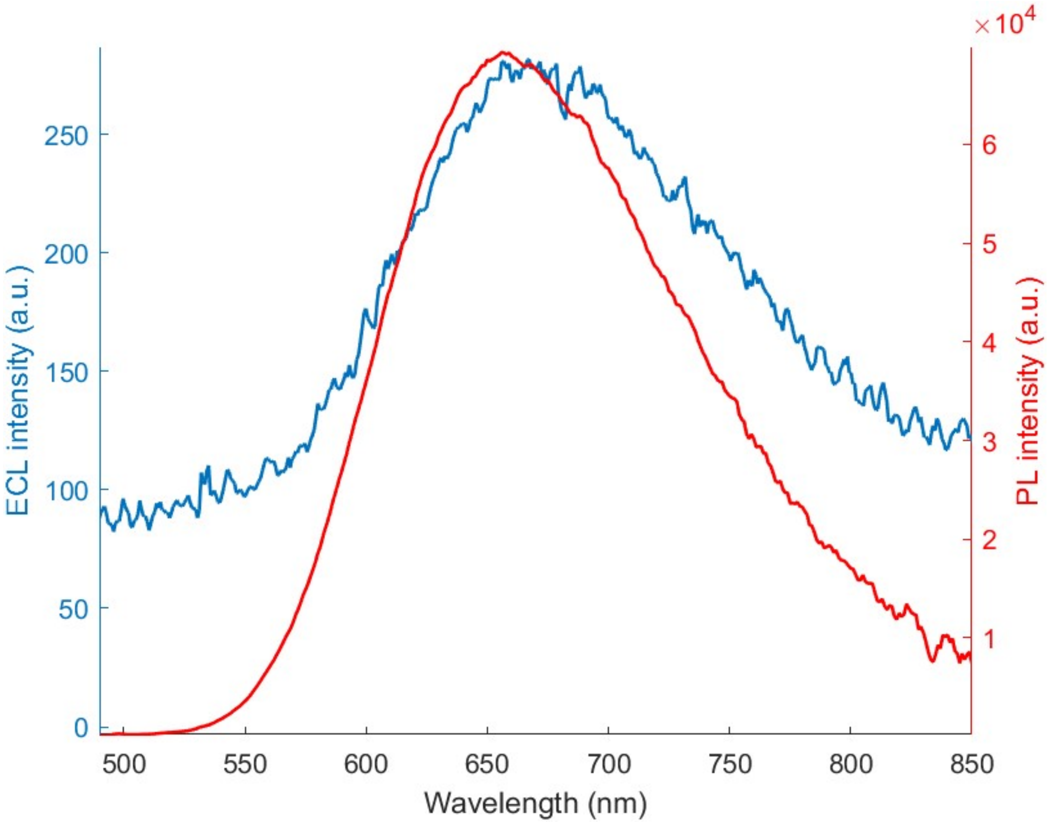

Figure S32: ECL (blue curve) and PL (red curve) spectra of 2^{tBu}CzDPPZ in dichloromethane.

NB: the comparison between ECL and PL spectra of $Na_4[4DPASO_3TPN]$ is not reported due to too low ECL emission of this compound.

5.3 Coreactant ECL-time and current-time profiles

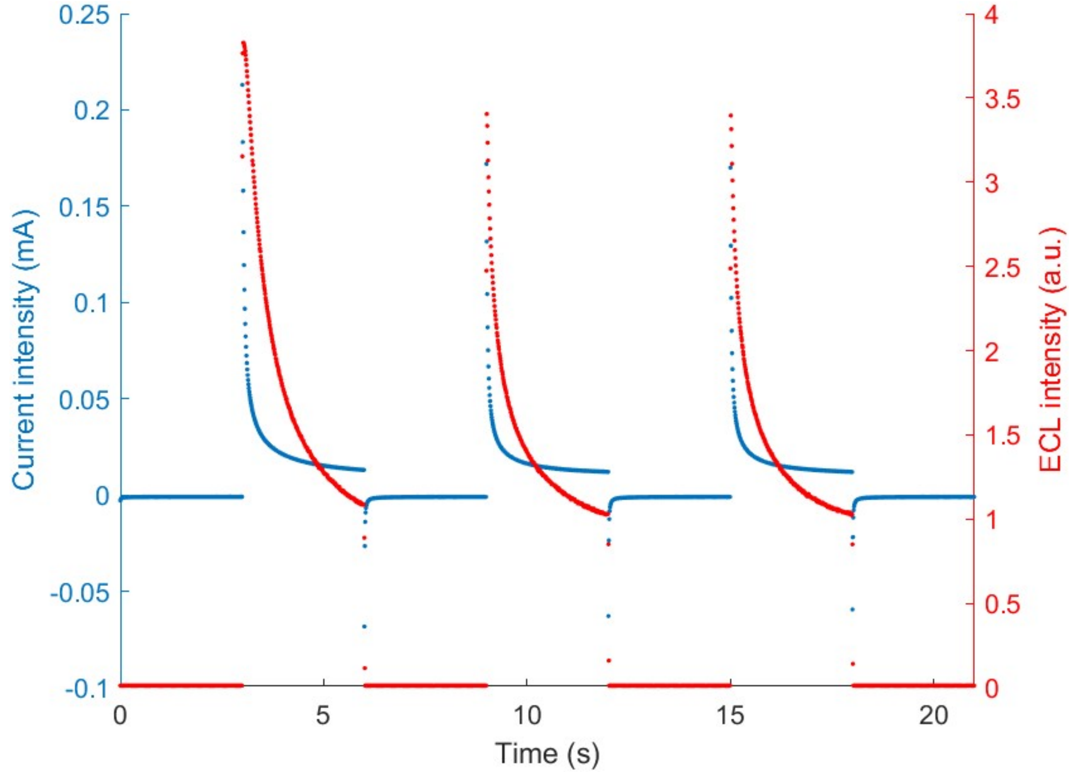

Figure S33: Coreactant ECL-time (red curve) and current-time (blue curve) profiles of 4DPATPN in dichloromethane.

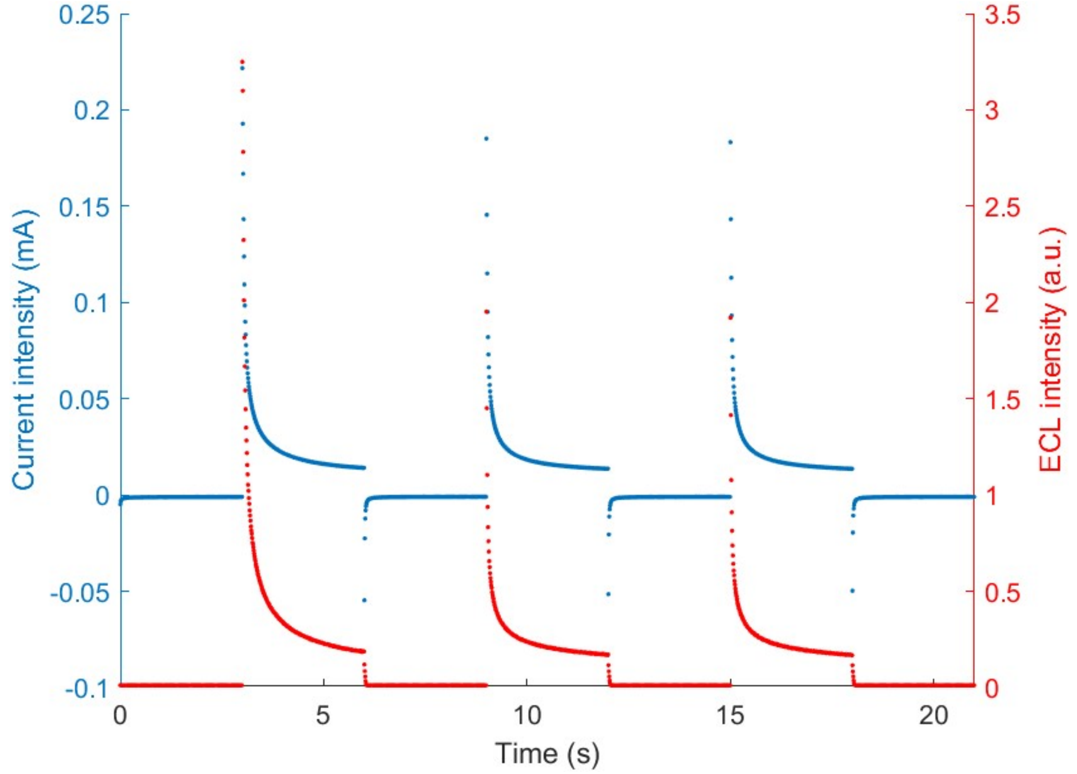

Figure S34: Coreactant ECL-time (red curve) and current-time (blue curve) profiles of 4DpTATPN in dichloromethane.

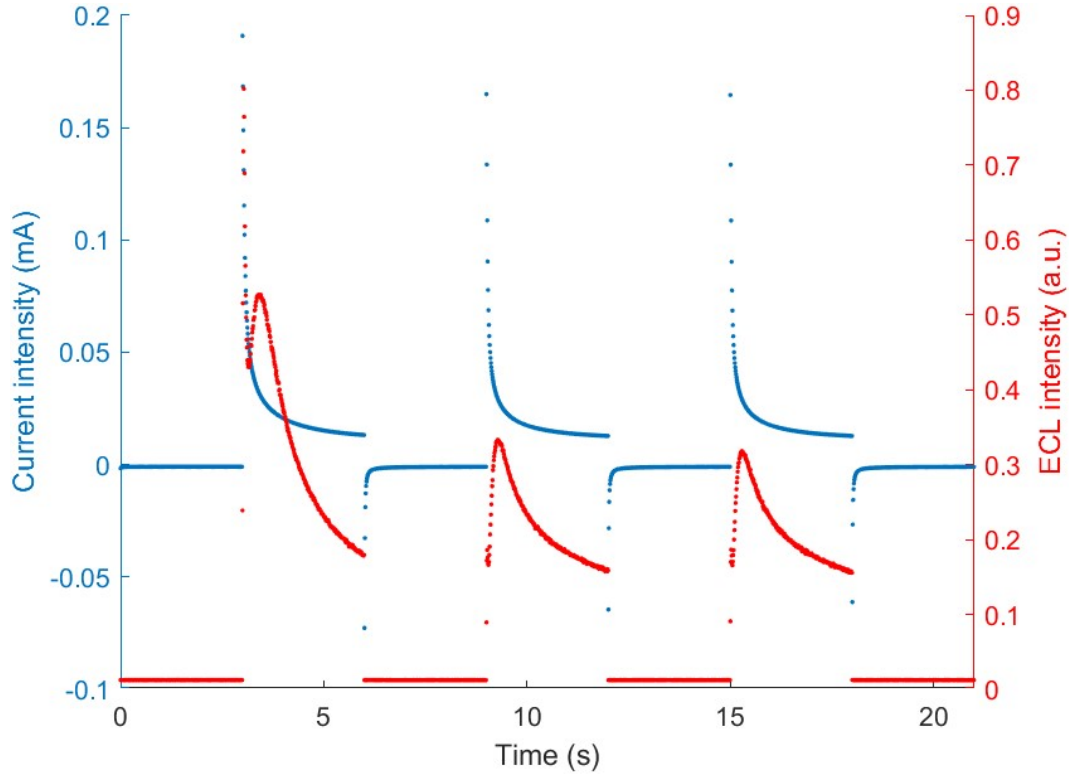

Figure S35: Coreactant ECL-time (red curve) and current-time (blue curve) profiles of 4^{tBu}CzTPN in dichloromethane.

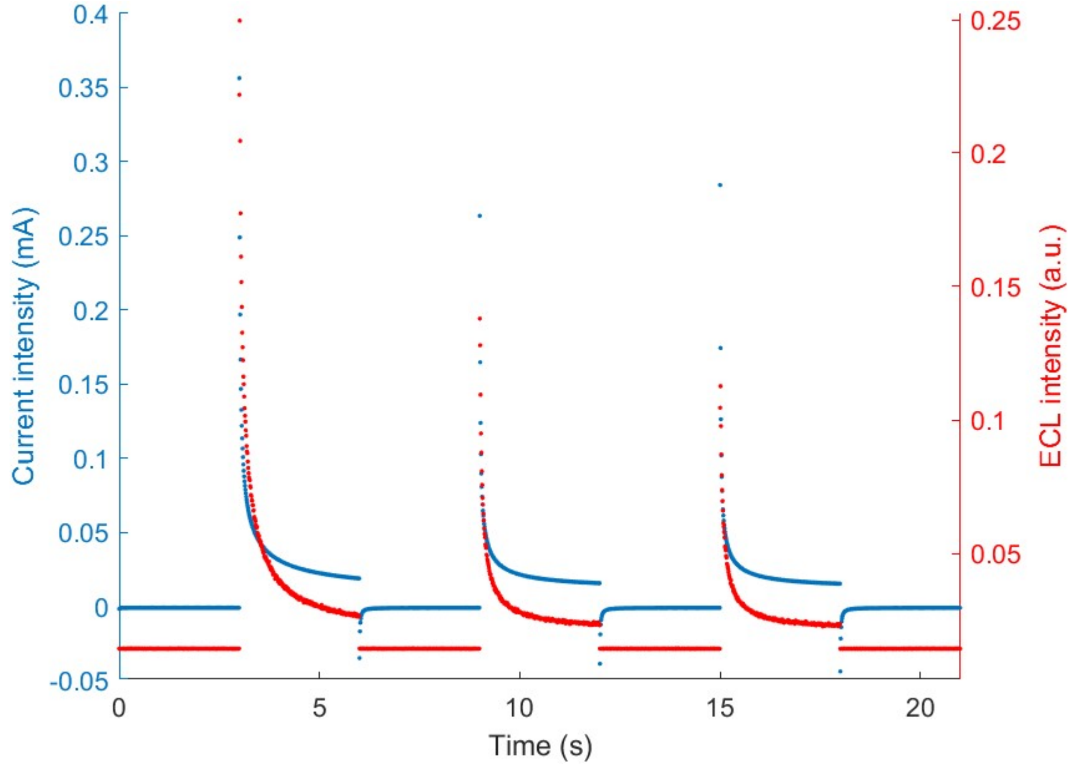

Figure S36: Coreactant ECL-time (red curve) and current-time (blue curve) profiles of 2DPADPPZ in dichloromethane.

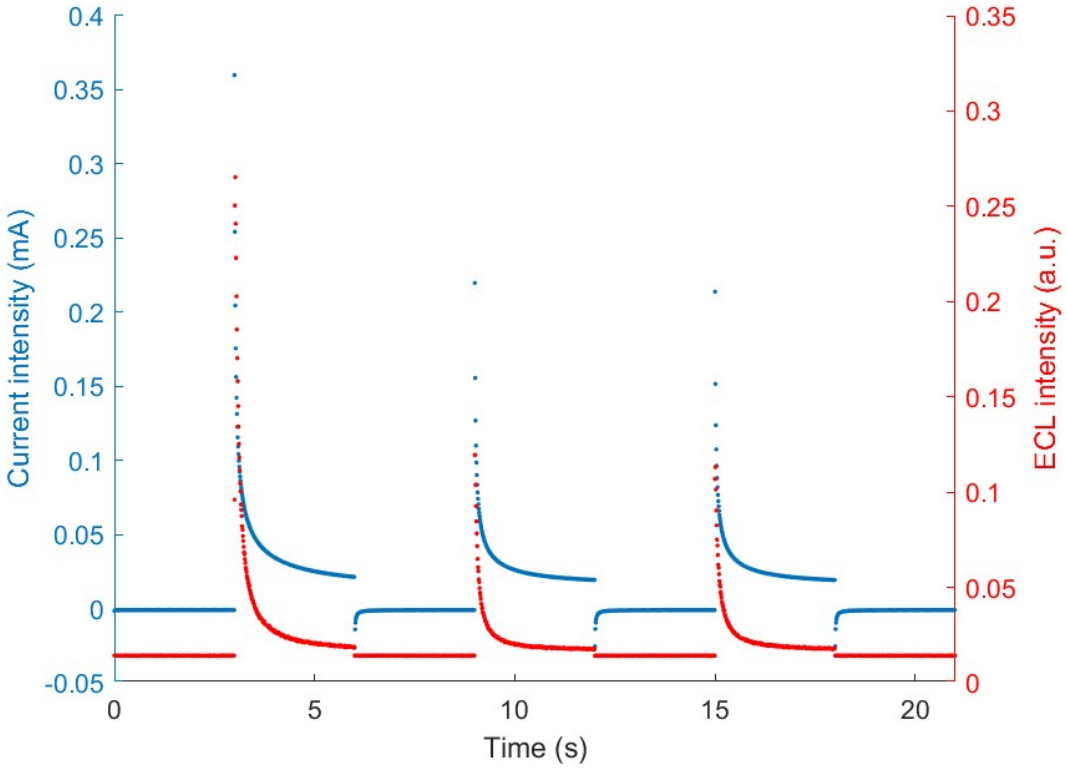

Figure S37: Coreactant ECL-time (red curve) and current-time (blue curve) profiles of 2^{tBu}CzDPPZ in dichloromethane.

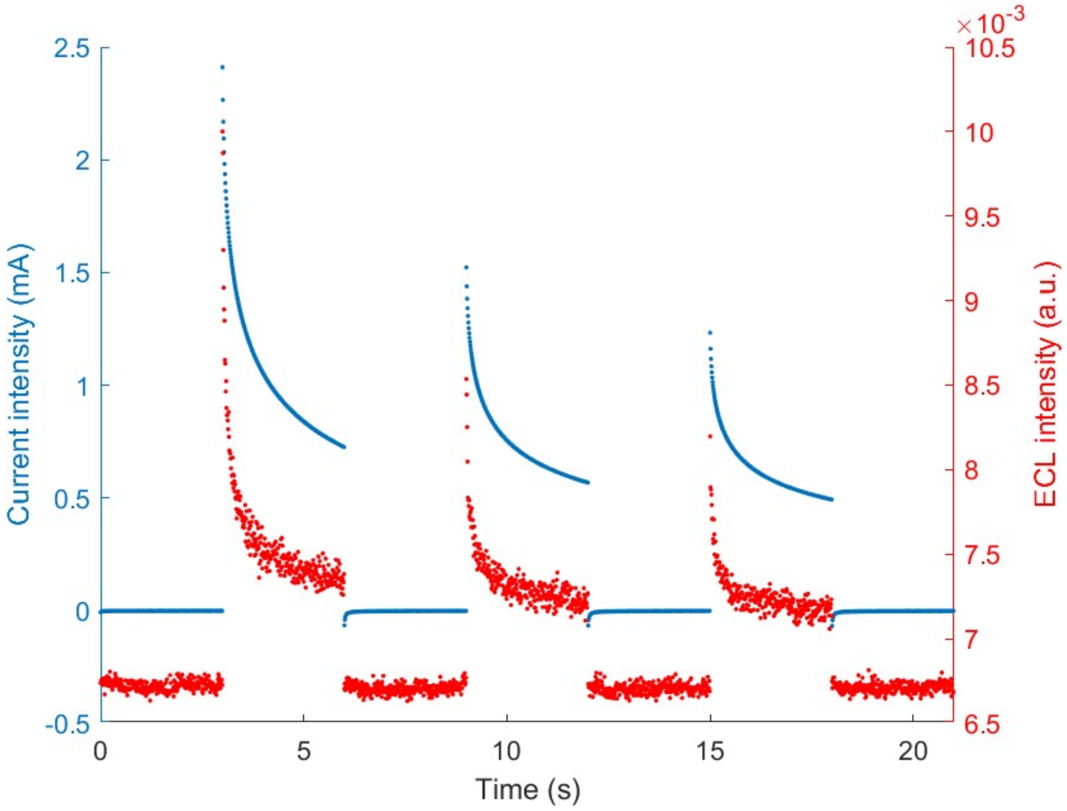

Figure S38: Coreactant ECL-time (red curve) and current-time (blue curve) profiles of 4DPASO$_3$TPN in ProCell.

5.4 Annihilation ECL-time and current-time profiles

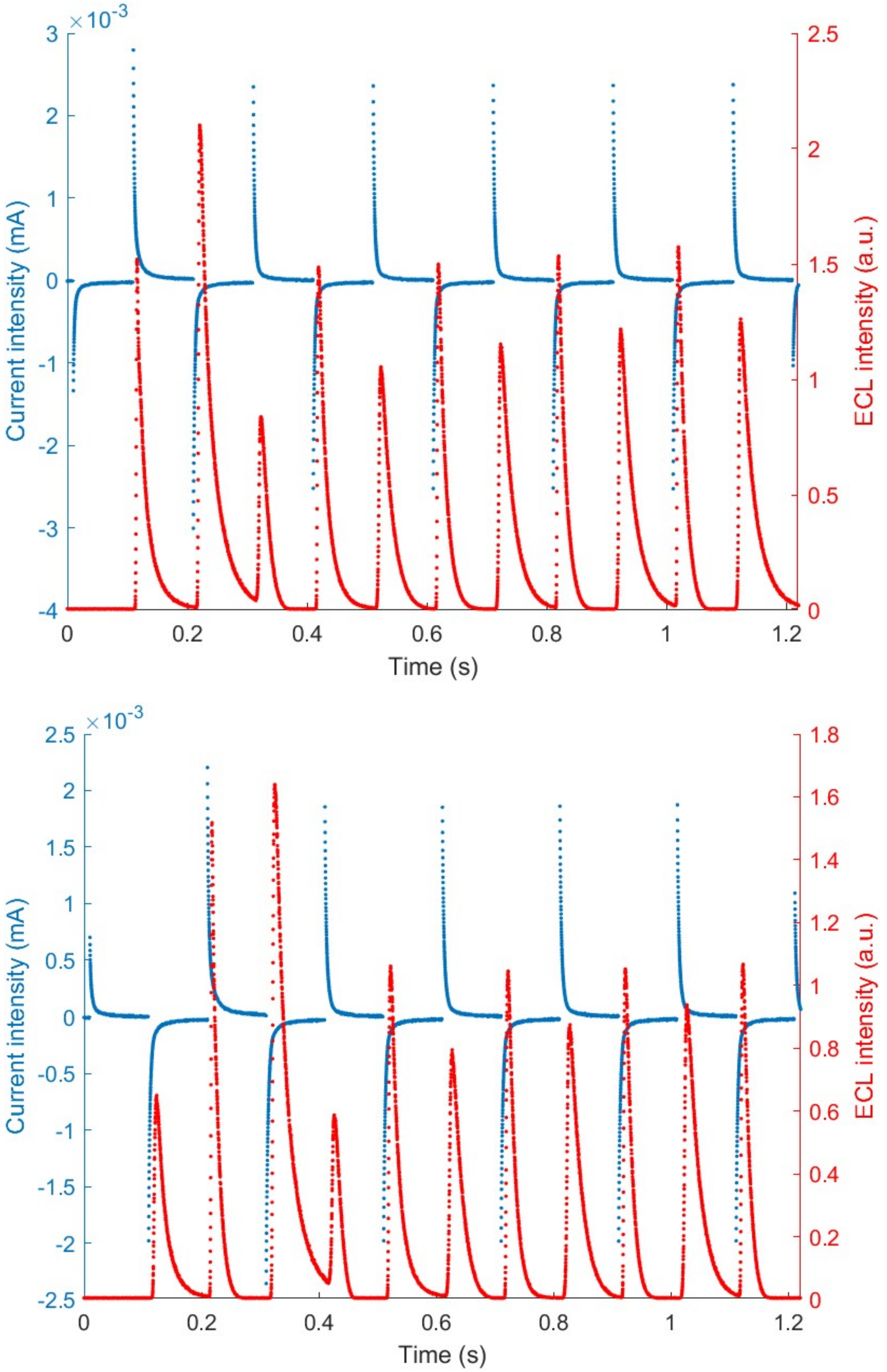

Figure S39: Annihilation ECL-time (red curve) and current-time (blue curve) profiles of 4DPATPN, –1.6 V to +1.1 V (top); +1.1 V to –1.6 V (bottom).

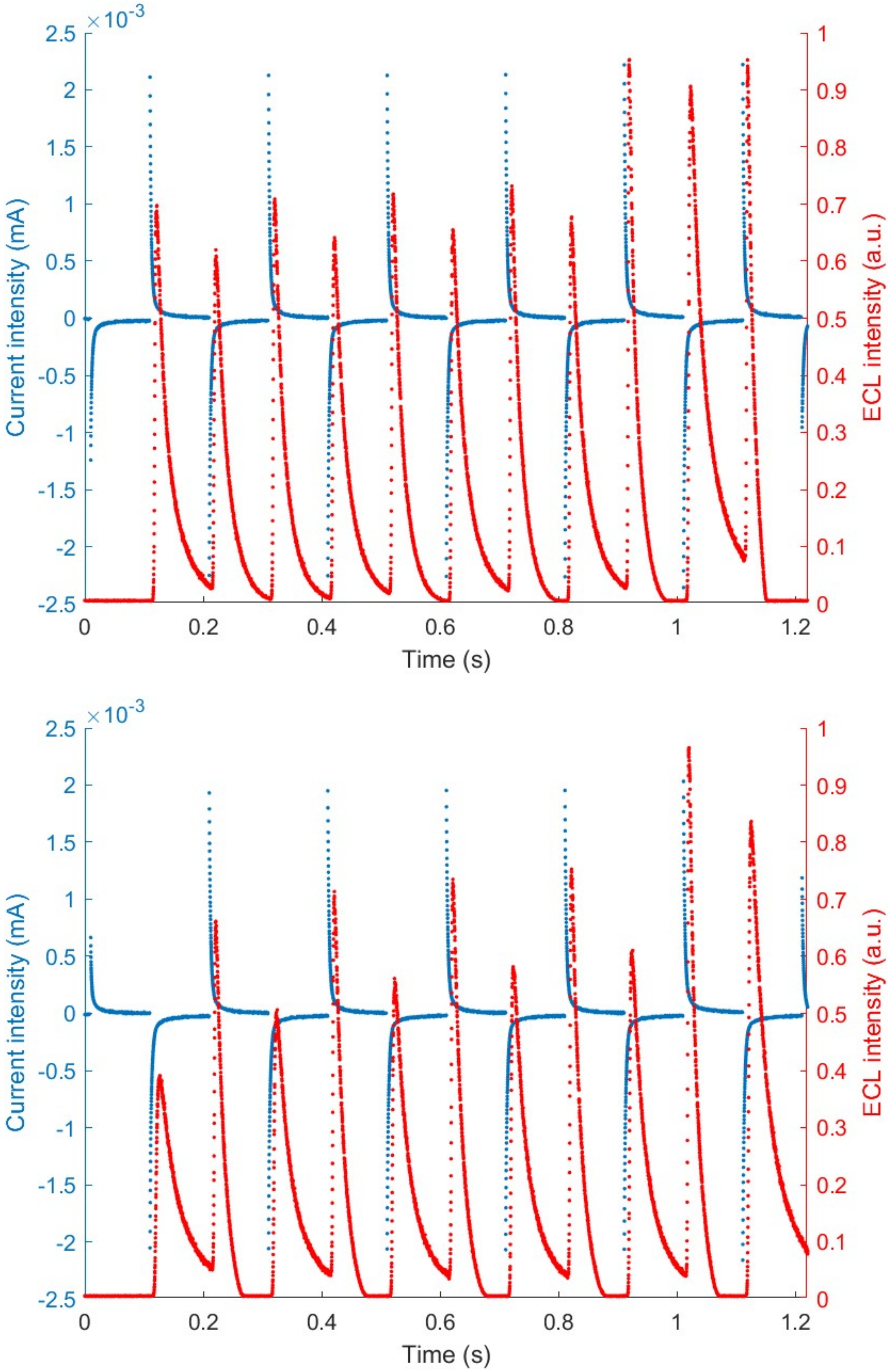

Figure S40: Annihilation ECL-time (red curve) and current-time (blue curve) profiles of 4DpTATPN –1.6 V to +1.0 V (top) +1.0 V to –1.6 V (bottom).

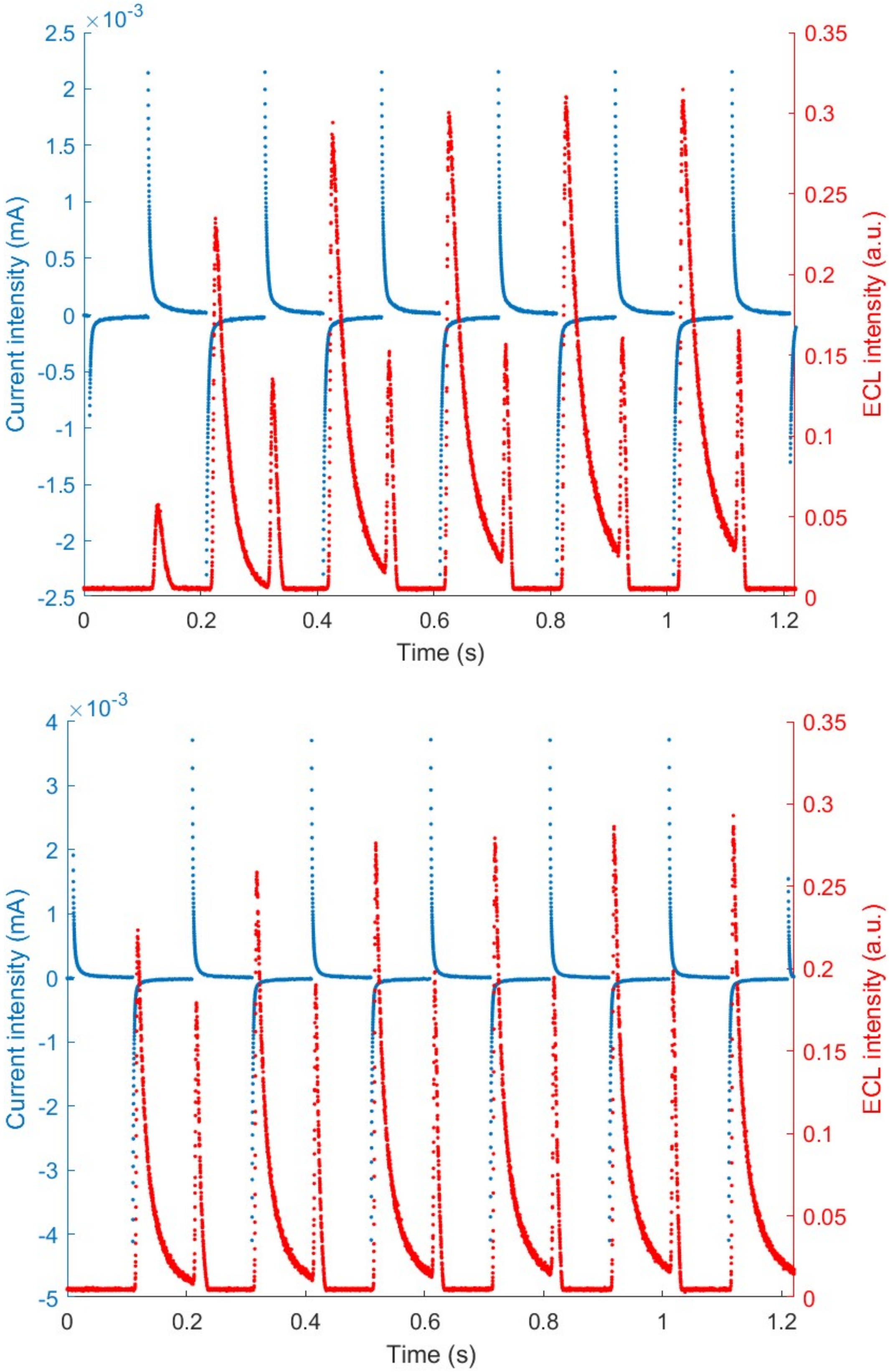

Figure S41: Annihilation ECL-time (red curve) and current-time (blue curve) profiles of 4^{tBu}CzTPN –1.2 V to +1.6 V (top) +1.6 V to –1.2 V (bottom).

6. Single crystal X-ray diffraction analysis

Table S1. Crystallographic data and refinement details.

	**4DpTATPN**	**4DPATPN**	**2DPADPPZ**
CCDC Number	2284117	2284118	2284119
Chemical Formula	$C_{64}H_{56}N_6\cdot{}^{1}/_{7}CHCl_3\cdot{}^{6}/_{7}C_6H_{14}$	$C_{56}H_{40}N_6\cdot 2C_3H_6O$	$C_{42}H_{28}Cl_9N_6\cdot 2CHCl_3\cdot 2C_6H_{14}$
Formula weight	1000.30 g/mol	913.09 g/mol	1147.15 g/mol
Temperature	100(2) K	100(2) K	100(2) K
Wavelength	0.620 Å	0.620 Å	0.620 Å
Crystal system	Monoclinic	Monoclinic	Monoclinic
Space Group	$P\ 2_1/c$	P 21/n	$C2/c$
Unit cell dimensions	a = 12.857(3) Å<br>b = 12.102(2) Å<br>c = 18.856(4) Å<br>α = 90°<br>β = 104.22(3)°<br>γ = 90°	a = 11.156(2) Å<br>b = 12.563(3) Å<br>c = 17.063(3) Å<br>α = 90°<br>β = 96.40(3)°<br>γ = 90°	a = 61.463(12) Å<br>b = 13.878(3) Å<br>c = 39.531(8) Å<br>α = 90°<br>β = 102.04(3)°<br>γ = 90°
Volume	2844.0(11) $Å^3$	2376.5(8) $Å^3$	32977(12) $Å^3$
Z	2	2	32
Density (calculated)	1.168 $g\cdot cm^{-3}$	1.276 $g\cdot cm^{-3}$	1.848 $g\cdot cm^{-3}$
Absorption coefficient	0.066 mm^{-1}	0.060 mm^{-1}	0.454 mm^{-1}
F(000)	1066	964	19072
Theta range for data collection	1.4° to 31.1°	1.8° to 31.1°	0.9° to 30.9°
Index ranges	$-20 \leq h \leq 20$,<br>$-20 \leq k \leq 20$,<br>$-30 \leq l \leq 29$	$-20 \leq h \leq 20$,<br>$-20 \leq k \leq 20$,<br>$-30 \leq l \leq 29$	$-100 \leq h \leq 99$,<br>$-22 \leq k \leq 22$,<br>$-65 \leq l \leq 65$
Resolution	0.60 Å	0.60 Å	0.60 Å
Reflections collected	66828	56097	393457
Independent reflections	12438, 9693 data with I>2σ(I)	10394, 9555 data with I>2σ(I)	72304, 29136 data with I>2σ(I)
Data multiplicity (max resltn)	4.79 (2.84)	4.81 (2.77)	4.96 (2.94)
I/σ(I) (max resltn)	19.58 (6.35)	25.55 (15.67)	5.73 (0.44)
R_{merge} (max resltn)	0.0361 (0.1422)	0.0390 (0.0565)	0.0987 (0.9403)
Data completeness (max resltn)	90.4% (74.0%)	90.4% (74.3%)	86.2% (67.9%)
Refinement method	Full-matrix least-squares on F^2	Full-matrix least-squares on F^2	Full-matrix least-squares on F^2
Data / restraints / parameters	12438 / 12 / 366	10394 / 0 / 318	72304/ 100 / 1891
Goodness-of-fit on F^2	1.061	1.043	1.005
Δ/σ_{max}	0.000	0.001	0.002
Final R indices [I>2□(I)]	R_1 = 0.0749, wR_2 = 0.2106	R_1 = 0.0437, wR_2 = 0.1255	R_1 = 0.0798, wR_2 = 0.2200
R indices (all data)	R_1 = 0.0902, wR_2 = 0.2234	R_1 = 0.0466, wR_2 = 0.1291	R_1 = 0.1763, wR_2 = 0.2767

Largest diff. peak and hole	0.633 and -0.394 $eÅ^{-3}$	0.586 and -0.280 $eÅ^{-3}$	1.378 and -1.013 $eÅ^{-3}$
R.M.S. deviation from mean	0.066 $eÅ^{-3}$	0.064 $eÅ^{-3}$	0.063 $eÅ^{-3}$

$$R_1 = \Sigma \left|\left|Fo\right| - \left|Fc\right|\right| / \Sigma \left|Fo\right|, \; wR_2 = \{\Sigma [w(Fo^2 - Fc^2)^2] / \Sigma [w(Fo^2)^2]\}^{½}$$

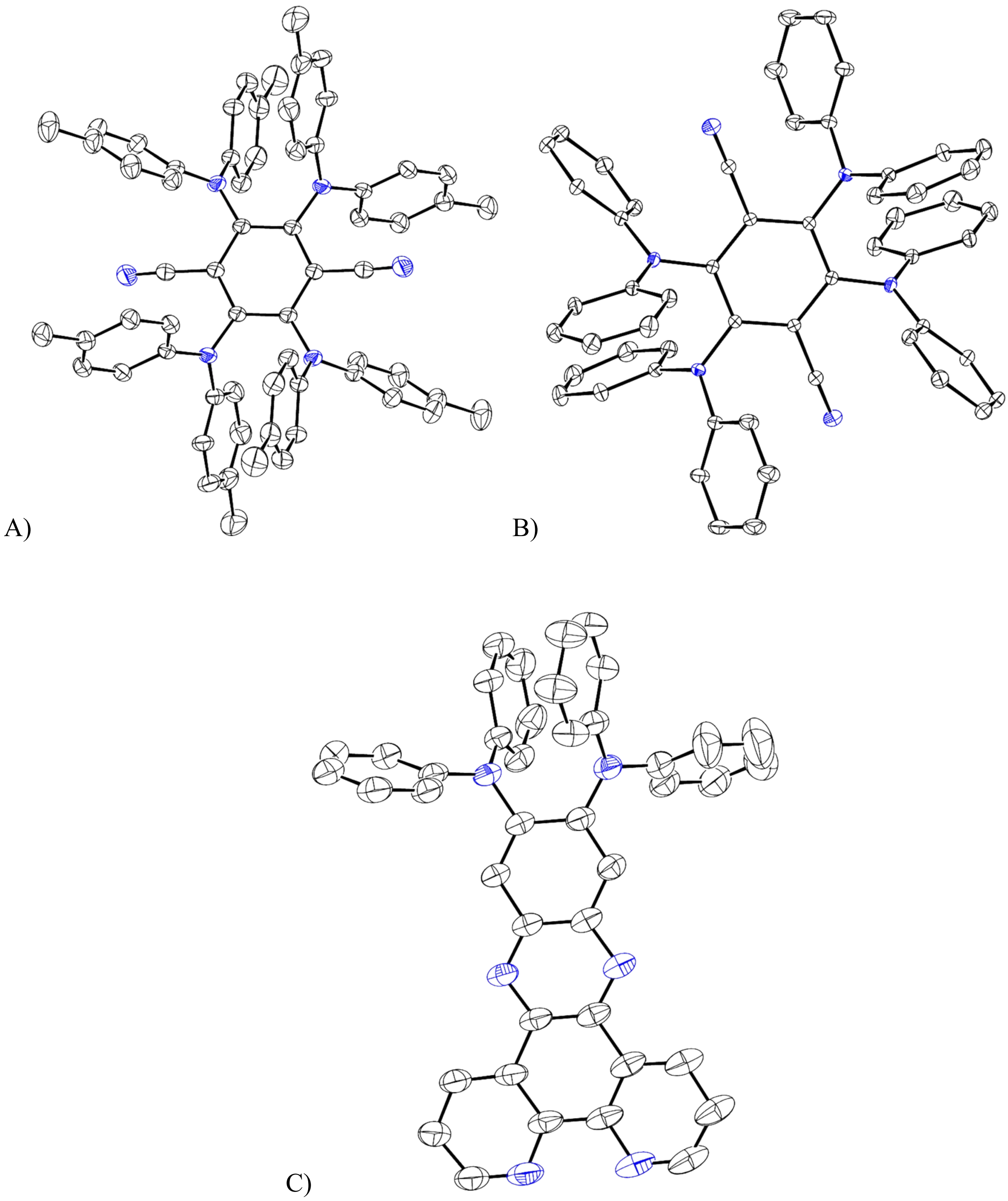

Figure S42: Ellipsoids representation of 4DpTATPN (A), 4DPATPN (B) and 2DPADPPZ (C) asymmetric unit contents (A.S.U. - 50% probability). Hydrogen atoms were omitted for clarity.

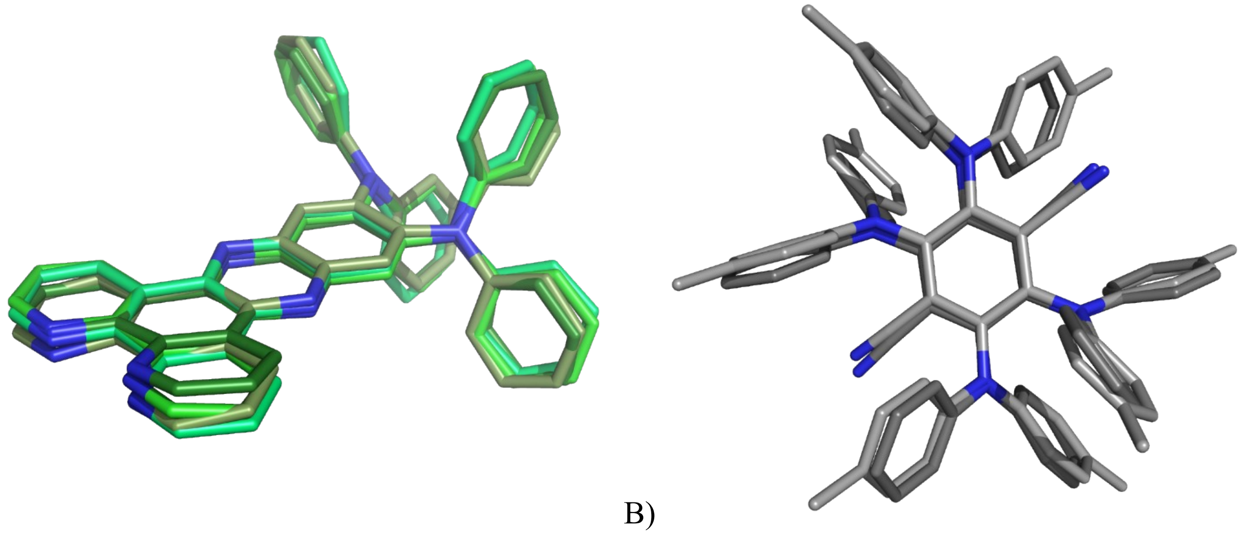

A) B)

Figure S43: Overlap of crystallographically independent molecules of 2DAPDPPZ (A) and 4DpTATPN with 4DPATPN (B) (hydrogens omitted for clarity).

7. Supplementary references

1. Y. Liu, M. Nishiura, Y. Wang and Z. Hou, *J. Am. Chem. Soc.*, 2006, **128**, 5592.
2. S. Roy, E. Colombo, R. Vinck, C. Mari, R. Rubbiani, M. Patra and G. Gasser, *ChemBioChem.*, 2020, **21**, 2966.
3. D. B. G. Williams and M. Lawton, *J. Org. Chem.*, 2010, **75**, 8351.
4. R. K. Harris, E. D. Becker, S. M. Cabral De Menezes, R. Goodfellow and P. Granger, *Pure Appl. Chem.*, 2001, **73**, 1795.
5. J. M. Thomas, *J. Chem. Educ.*, 1999, **76**, 97.
6. M. M. Richter, *Chem. Rev.*, 2004, **104**, 3003.
7. A. Lausi et al. *The European Physical Journal Plus*, 2015, **130**, 1.
8. W. Kabsch *Acta Crystallographica Section D*, 2010, **66**, 125.
9. G. M. Scheldrick, *Acta Crystallographica Section A*, 2015, **71**, 3.
10. G. M. Scheldrick, *Acta Crystallographica Section C*, 2015, **71**, 3.
11. P. Emsley et al., *Acta Crystallographica Section D*, 2010, **66**, 486.
12. A. Spek, *Acta Crystallographica Section C*, 2015, **71**, 9.
13. Farrugia L., *Journal of Applied Crystallography*, 2012, **45**, 849.
14. Schrodinger L., The PyMOL Molecular Graphics System. Schrodinger, LLC, http://www.pymol.org, 2015.
15. N. Noto, A. Yada, T. Yanai and S. Saito, *Angew. Chem. Int. Ed.*, 2023, **62**, e2022191.

8. ^{1}H and ^{13}C{^{1}H} NMR spectra of compounds

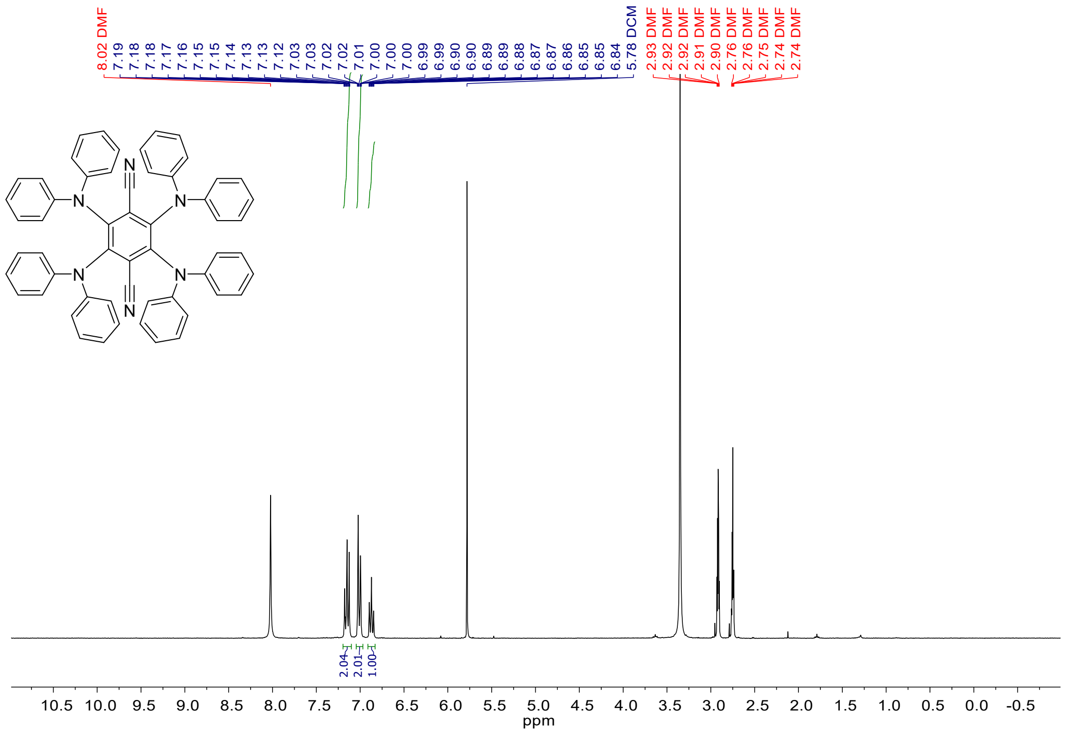

Figure S44: ^{1}H NMR (300 MHz, DMF-d_7) spectrum of 4DPATPN.

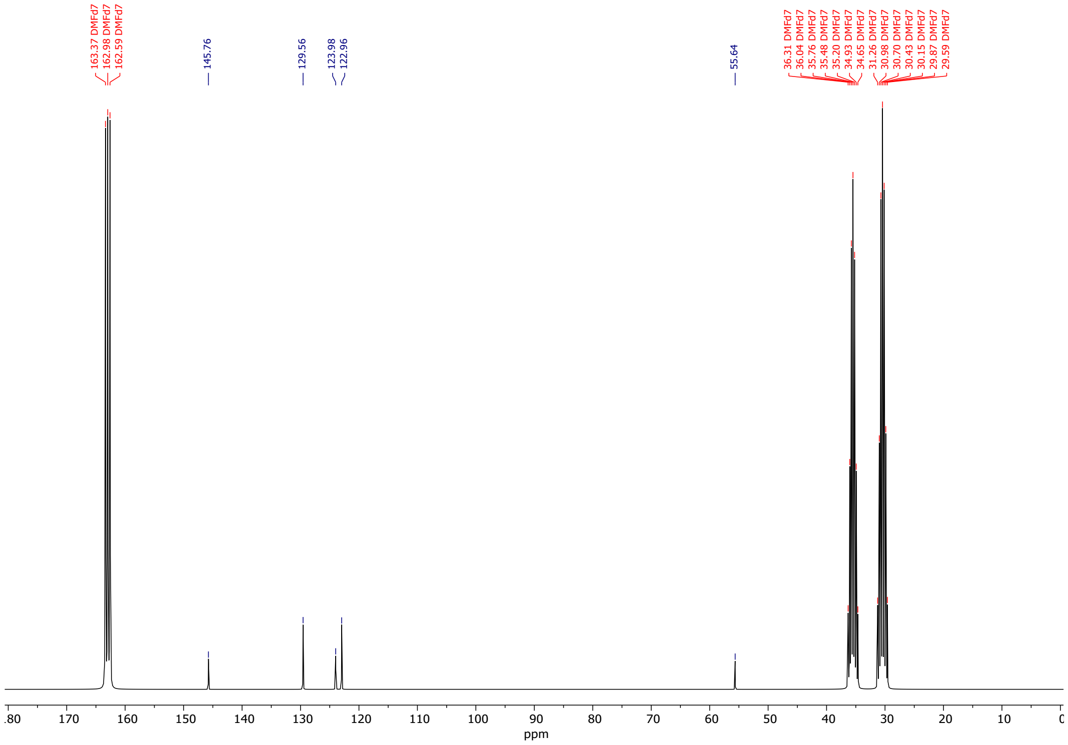

Figure S45: ^{13}C NMR (75 MHz, DMF-d_7) spectrum of 4DPATPN.

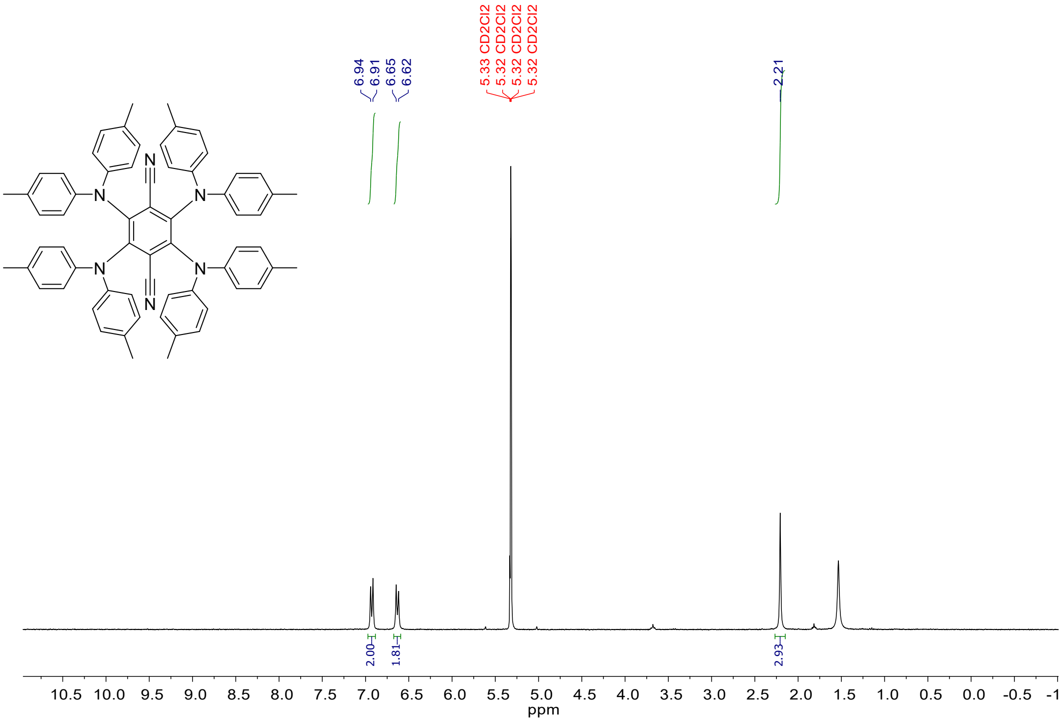

Figure S46: ^{1}H NMR (300 MHz, CD_2Cl_2) spectrum of 4DpTATPN.

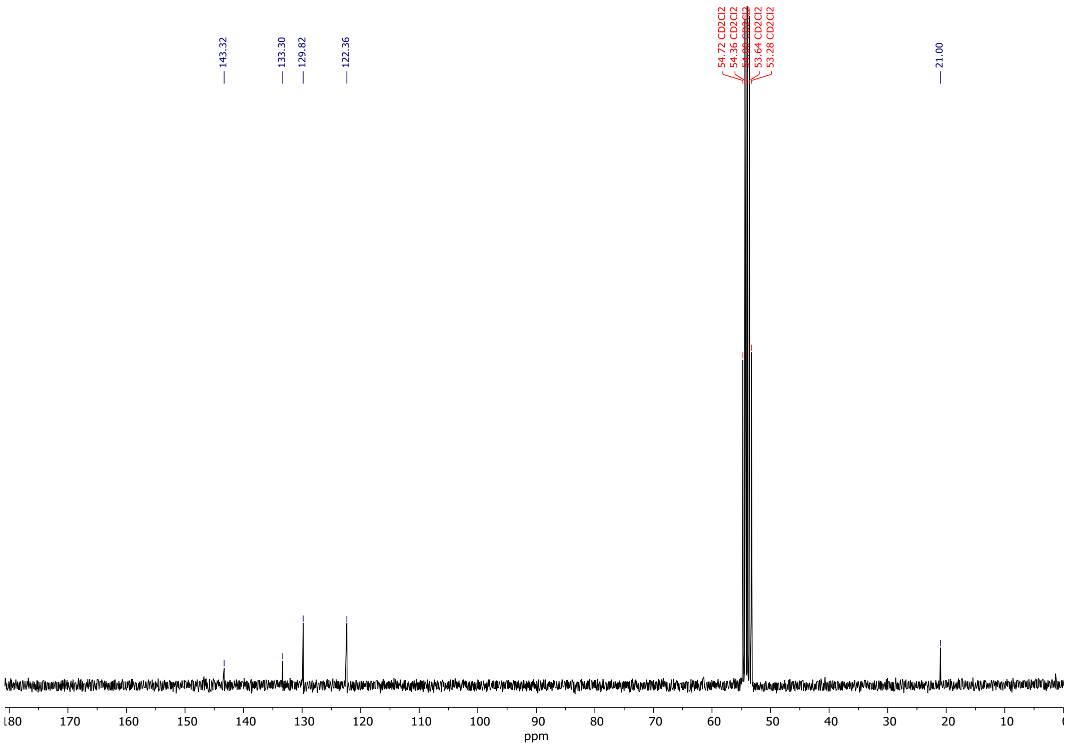

Figure S47: ^{13}C NMR (75 MHz, CD_2Cl_2) spectrum of 4DpTATPN.

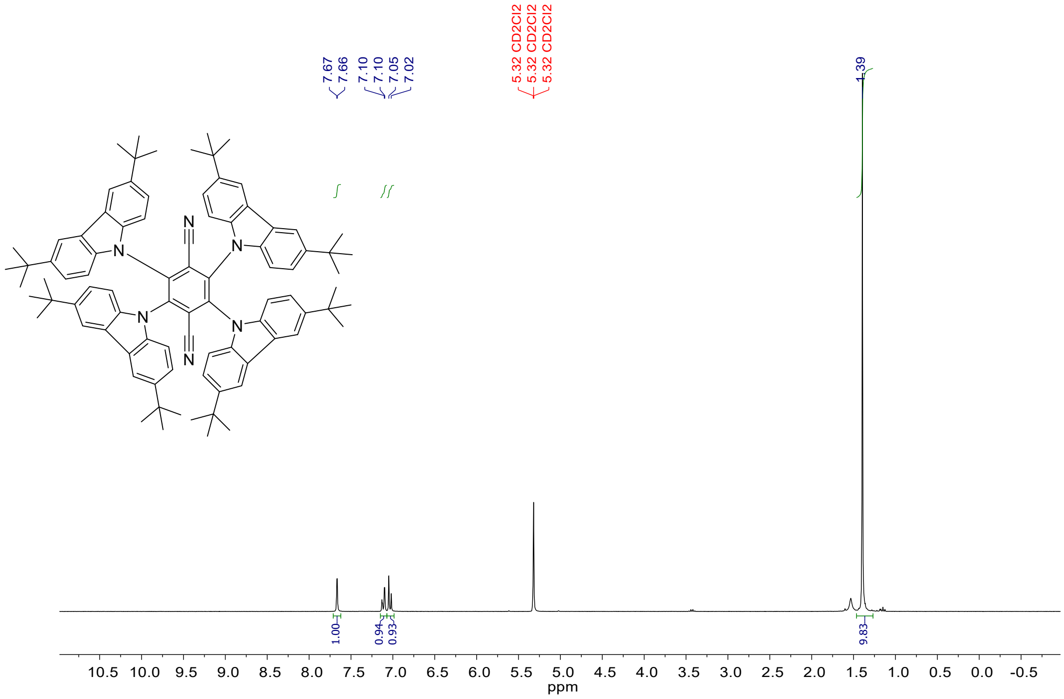

Figure S48: ^{1}H NMR (300 MHz, CD_2Cl_2) spectrum of 4^{tBu}CzTPN.

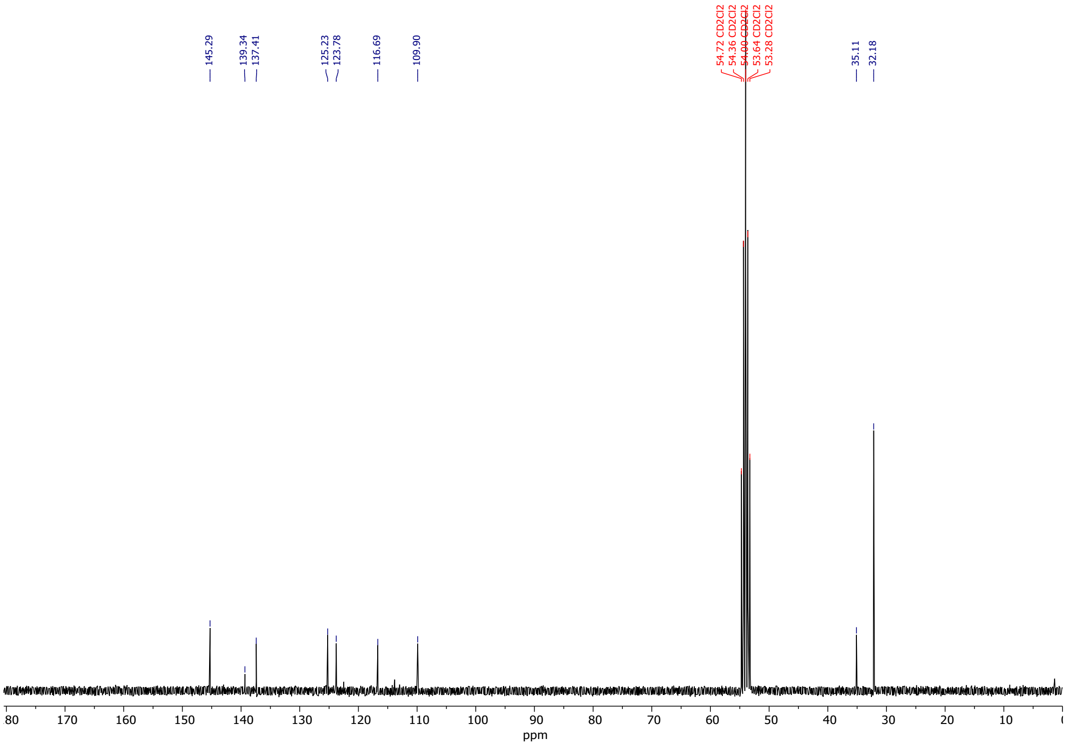

Figure S49: ^{13}C NMR (75 MHz, CD_2Cl_2) spectrum of 4^{tBu}CzTPN.

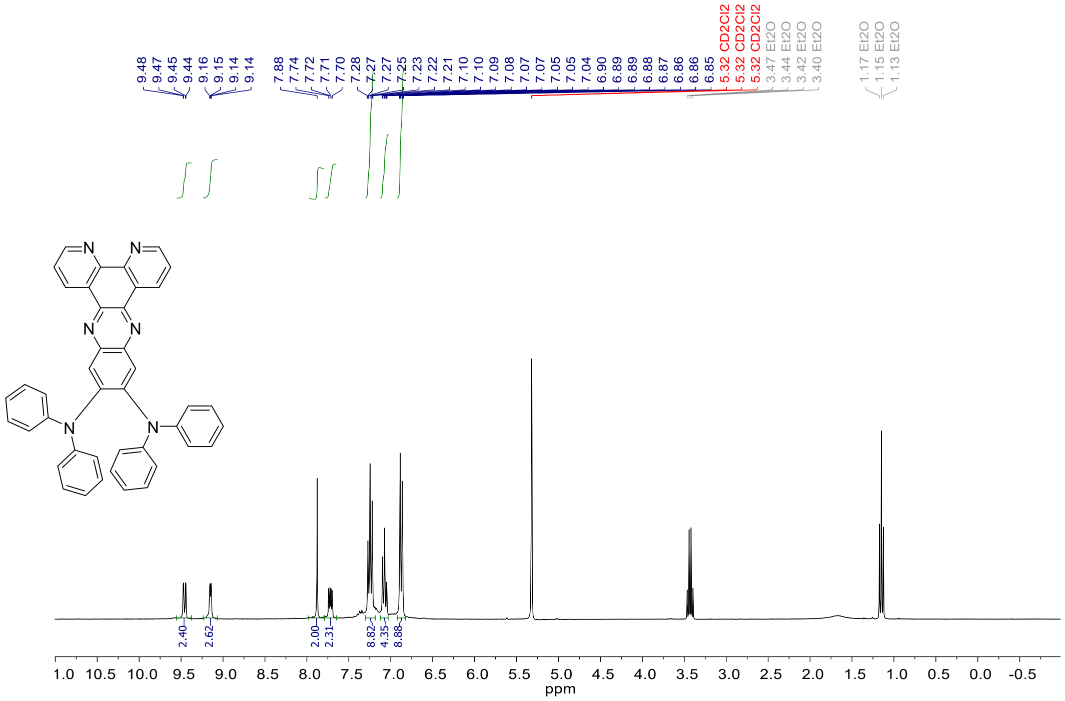

Figure S50: ^{1}H NMR (300 MHz, CD_2Cl_2) spectrum of 2DPADPPZ.

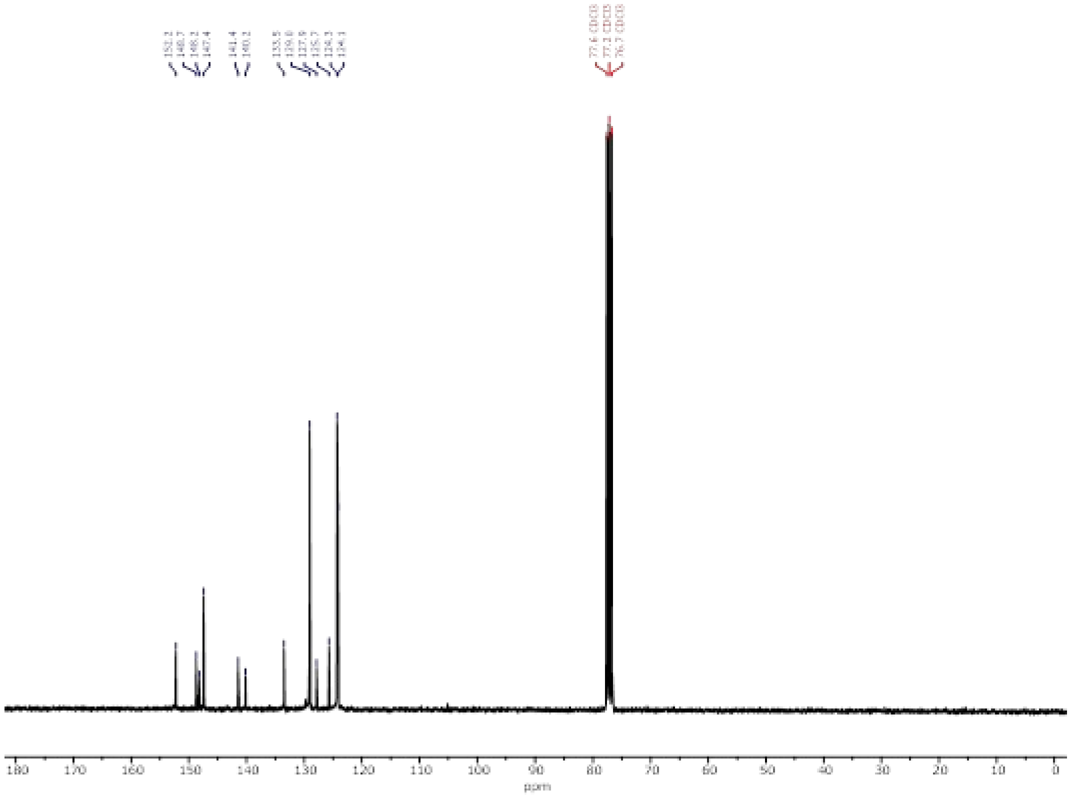

Figure S51: ^{13}C NMR (75 MHz, CD_2Cl_2) spectrum of 2DPADPPZ.

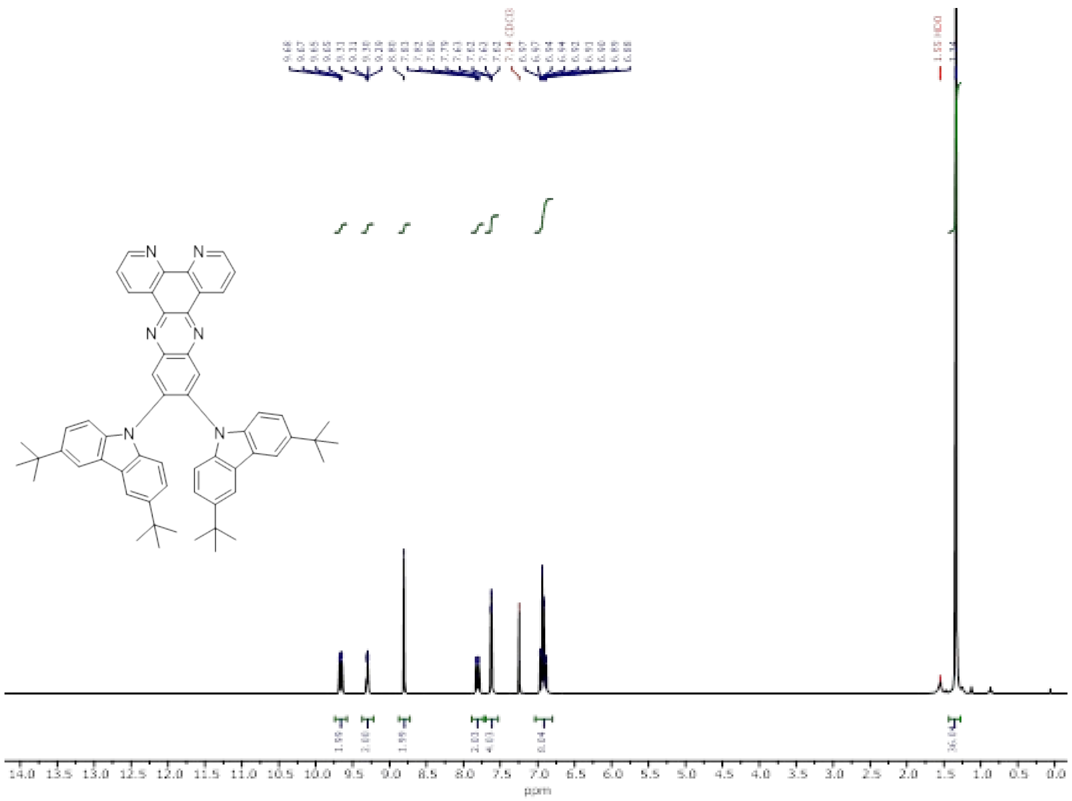

Figure S52: ^{1}H NMR (300 MHz, CD_2Cl_2) spectrum of 2^{tBu}CzDPPZ.

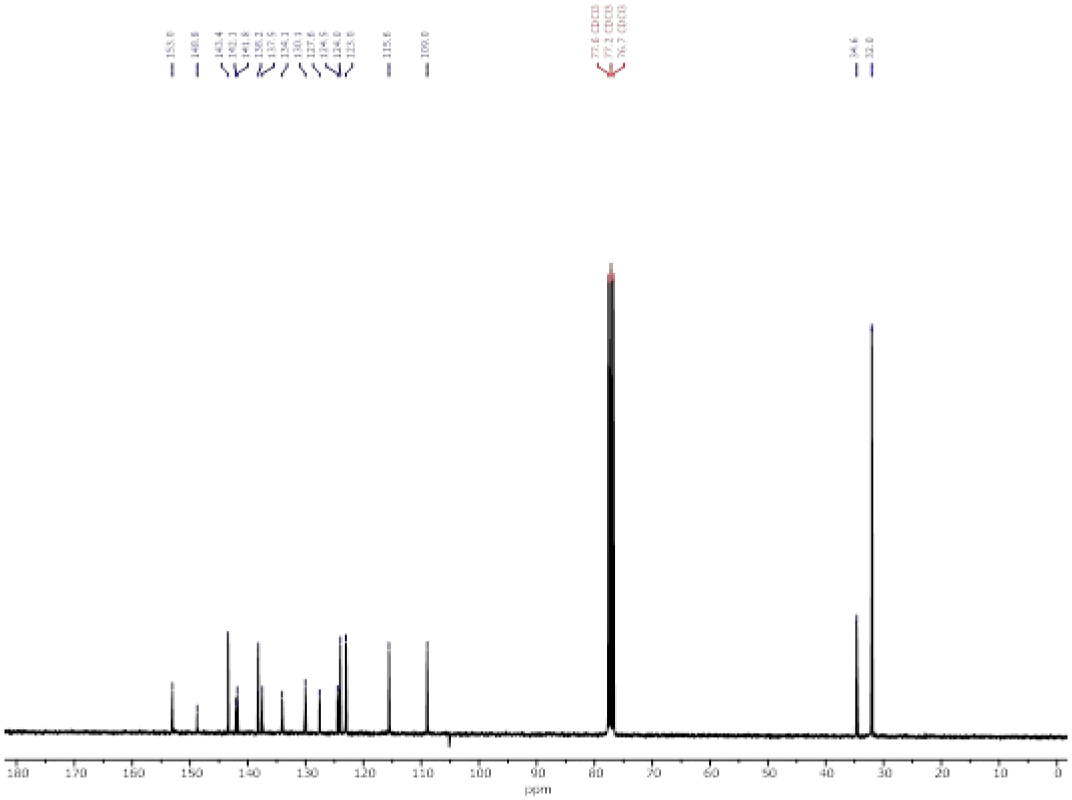

Figure S53: ^{13}C NMR (75 MHz, CD_2Cl_2) spectrum of 2^{tBu}CzDPPZ.

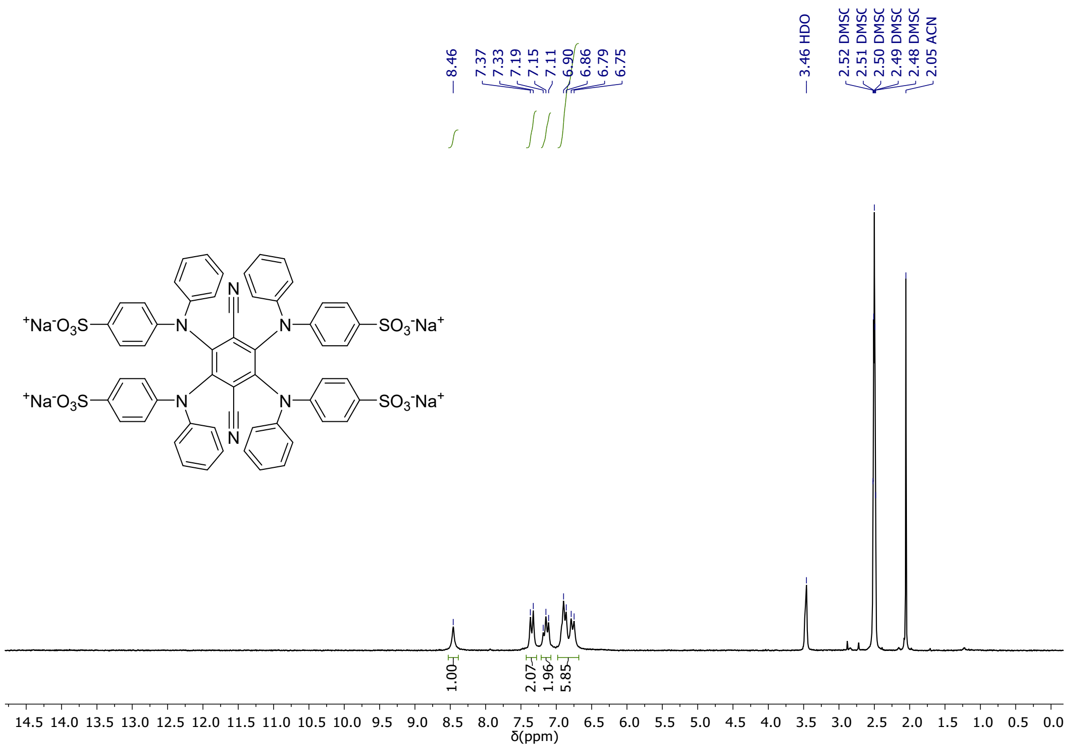

Figure S54: ^{1}H NMR (300 MHz, DMSO-d_6) spectrum of Na_4[4DPASO$_3$TPN].

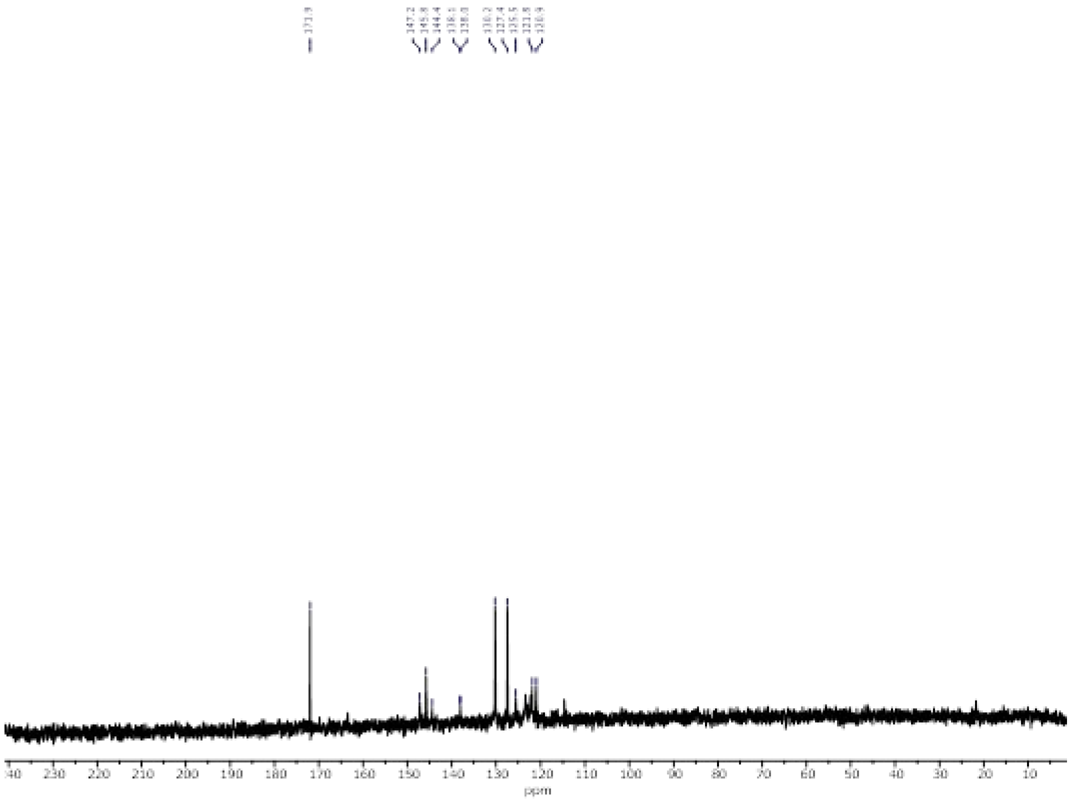

Figure S55: ^{13}C NMR (75 MHz, D_2O) spectrum of Na_4[4DPASO$_3$TPN].
